# Delivery-Graded Programmable Micelles Achieve Enhanced Tumor Starvation through Combined Glutamine Deprivation and Angiogenesis Inhibition

**DOI:** 10.34133/research.0858

**Published:** 2025-09-05

**Authors:** Xuan Wei, Jiamin Cheng, Meijuan Geng, Siyu Chen, Liyang Gong, Siyu Meng, Keying Chen, Ziyan Wang, Zhang Yuan, Kaiyong Cai, Liangliang Dai

**Affiliations:** ^1^ Institute of Medical Research, Northwestern Polytechnical University, Xi’an, China.; ^2^ Key Laboratory of Biorheological Science and Technology, Ministry of Education, College of Bioengineering, Chongqing University, Chongqing, China.

## Abstract

The inhibition of dependent glutamine metabolism is an effective treatment for triple-negative breast cancer (TNBC) starvation, but it is limited by compensatory glycolysis and inadequate delivery efficiency. Herein, we construct a pH-responsive size/charge-reprogrammed micelle with hierarchical delivery characteristics for TNBC suppression with glutamine depletion and vessel blockade. It consists of a positively charged prodrug micelle chemically grafted with the glutamine transport inhibitor V9302 as the inner core layer, the neovascular disruptor CA4P adsorbed in the middle layer, and a pH-responsive peelable polymer as the outer shell. The nanosystem PPD/PPQV@C could effectively reduce size and reverse charge in response to the tumor acidic microenvironment by removing the outer polymer PPD, as accompanying the release of CA4P. Furthermore, the remaining PPQV could responsively release V9302 in the cytoplasm of tumor cells, improving the bioavailability of cargoes and overcoming permeability barrier through precise hierarchical release strategy. Importantly, V9302 and CA4P localized in the tumor intracellular and extracellular matrix could effectively block TNBC-dependent glutamine metabolism and inhibit compensatory nutrient by blocking angiogenesis, achieving the desired tumor suppression with prolonged survival time. This work exhibits a smart nanoplatform for efficient TNBC treatment via dual blockade of the dependent glutamine metabolism and angiogenesis.

## Introduction

Glutamine metabolism was highly dependent on breast cancer cells compared to normal cells [1–3]. As an unnatural amino acid, glutamine was not only an important precursor for cellular energy production, biomass synthesis, and cell signal transduction [4,5] but also a leader in maintaining cellular homeostasis and reducing oxidative damage through metabolic conversion to glutathione (GSH) [6–9]. Therefore, starvation therapy represented by inhibition of glutamine metabolism was an effective treatment strategy for breast cancer [10,11]. However, glutamine blockade alone showed the limited therapeutic effects for breast cancer, resulting from the compensatory glycolysis, oxidative phosphorylation (OXPHOS), and fatty acid metabolisms [12,13]. Glucose and free fatty acids delivered via vascular transport were key upstream drivers of these compensatory metabolisms [14,15]. Consequently, disrupting the tumor vasculature alone was insufficient to eradicate entire tumors, because residual tumor cells that were far away from blood vessels could get essential nutrients (e.g., glutamine) from other cells in the tumor microenvironment [16–19]. Therefore, the combination of directly anti-angiogenic therapy with glutamine metabolism inhibition strategy possessed a great potential for improving breast cancer killing efficiency.

Typically, V9302 was a well-known glutamine metabolism inhibitor, which could block glutamine uptake and metabolism of tumor cells by inhibiting the activity of alanine–serine–cysteine transporter 2 (ASCT2), a sodium-dependent solute carrier protein responsible for the primary transporter of glutamine in cancer cells [20–22]. The suppression of glutamine metabolism induced by V9302 exhibited the modern antitumor effect by interfering cell growth and increasing oxidative damage in previous studies [21,23]; CA4P, as a structural analog of colchicine [24], was frequently investigated in vascular disrupting studies, which could disrupt endothelial cell–cell junctions by inhibiting the polymerization of the tubulin cytoskeleton, leading to endothelial cell mitotic arrest, endothelial cell morphological changes, vascular leakage, and subsequent cell necrosis by selectively blocking endothelial cell-specific linkers and vascular endothelial cadherin [25–29]. However, the different sites of action of the above 2 therapeutic drugs are as follows: the main work site of CA4P was in the extracellular matrix (ECM), which was used to block the neovascularization of tumor tissues, while V9302 normally binds to the intracellular transmembrane protein ASCT2 to block glutamine [30,31]. The constraints of the biological barrier represented by the dense ECM and inefficient tumor cell uptake pose serious challenges to the effective treatment of breast cancer [32]. Therefore, developing a smart nanoplatform to achieve selective fractional delivery of 2 drugs to the tumor extracellular and intracellular matrix was an attractive solution to address these issues [33]. This strategy achieved precise tumor therapy with high efficiency through concurrent blockade of glutamine-dependent metabolism and direct disruption of tumor vasculature, depriving tumors of essential nutrients and energy substrates.

**Fig. 1.**
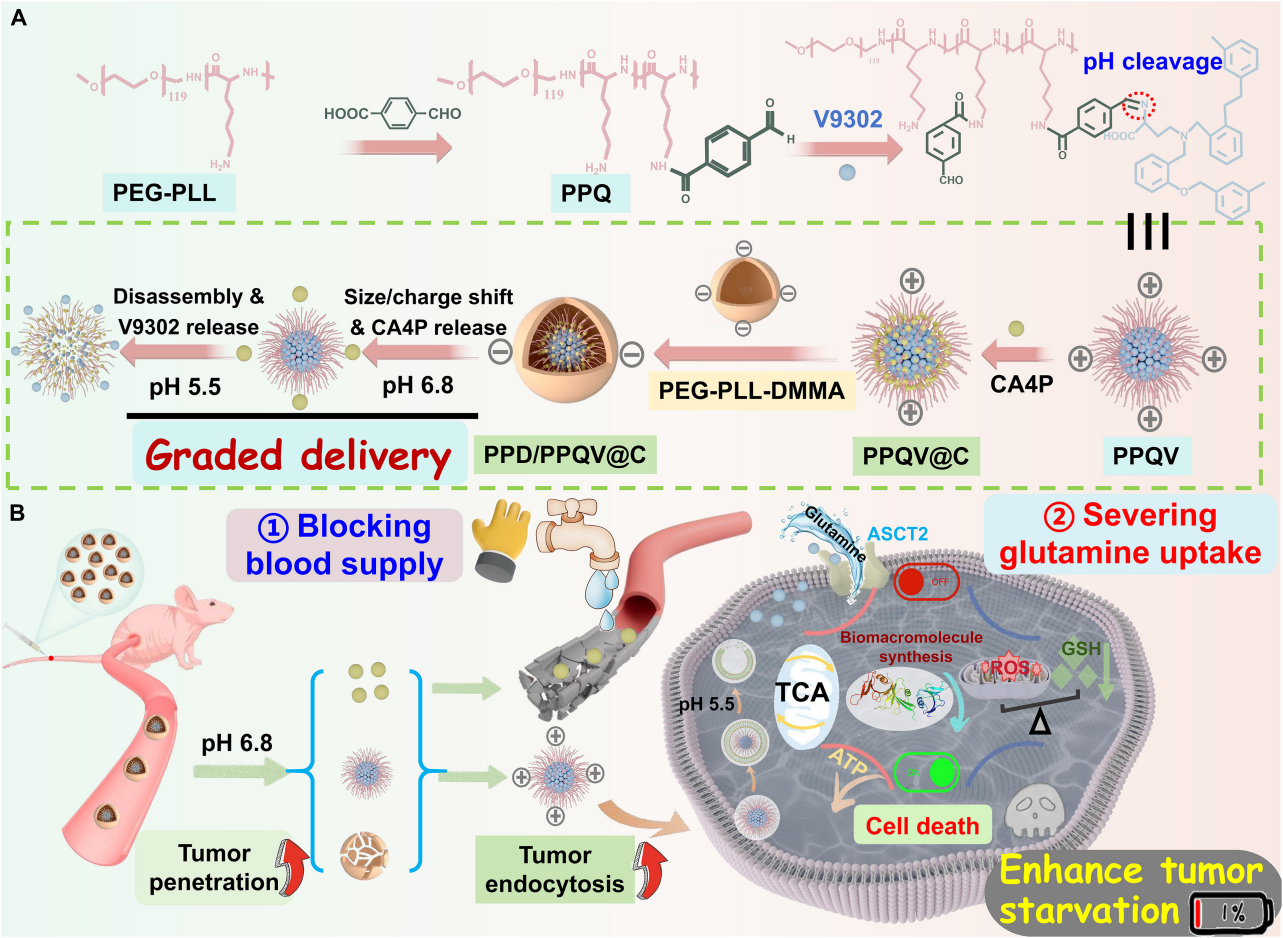
Construction of the PPD/PPQV@C micelle nanosystem and their antitumor therapeutic capabilities. (A) Synthetic route and (B) schematic illustration of the PPD/PPQV@C nanosystem for enhanced TNBC starvation with blockade of glutamine metabolism and angiogenesis.

Herein, a size/charge programmable micellar nanosystem with staged delivery feature was developed for triple-negative breast cancer (TNBC) therapy with glutamine depletion and vessel blocking (Fig. 1). Briefly, the hydrophilic segment poly (ethylene glycol)-b-poly (l-lysine) (PEG-PLL) polymer was first synthesized and the multiple V9302 molecules were covalently conjugated on this backbone via the linker-4-formylbenzoic acid, forming the acid-sensitive amphiphilic prodrug micelle (denoted PPQV) with positive change trait. The negatively charged CA4P was then adsorbed on the surface of PPQV (named as PPQV@C), followed by capping with the weakly acidic stimulus (pH 6.8)-response outer polymer poly (ethylene glycol)-b-poly (l-lysine)-dimethylmaleic anhydride (PEG-PLL-DMMA, abbreviated as PPD) through electrostatic adsorption, ultimately yielding the PPD/PPQV@C nanosystem (Fig. 1A). After intravenous injection, PPD/PPQV@C preferentially accumulated in tumors through the enhanced permeability and retention effect [34] and electrostatic rejection. The latter could prevent nonspecific uptake by negatively charged healthy tissues/cells, leading to the prolonger blood circulation time [35]. Furthermore, DMMA on the outer PPD polymer ionized in response to the low-acid tumor microenvironment and detached from the nanosystem through charge repulsion, resulting in the size reduction of the nanosystem and the release of CA4P in the ECM. Importantly, the resultant PPQV with small size and positive charge could effectively penetrate the deep sites of tumors and be endocytosed by tumor cells, which was then disintegrated in response to low pH in the lysosomes and released V9302 in the cytoplasm, achieving the staged delivery. Notably, CA4P and V9302 could effectively block angiogenesis and inhibit its dependent glutamine metabolism in TNBC, achieving the desired antitumor effect (Fig. 1B). This study provided a pH-responsive smart nanoplatform with size reduction and charge conversion and staged drug delivery capabilities for the enhanced TNBC therapy with blockade of glutamine metabolism and suppression of angiogenesis.

## Results and Discussion

### Synthesis and characterization of PPD/PPQV@C nanosystem

In order to prepare the pH-responsive prodrug micellar nanosystem PPD/PPQV@C, the previously synthesized PEG-PLL [36] was firstly modified with 4-formylbenzoic acid (denoted as PPQ), which was then covalently grafted with multiple V9302 hydrophilic via the pH-sensitive (pH 5.5) Schiff base linkage [37], yielding a pH-responsive amphiphilic prodrug micelle PPQV. Meanwhile, the pH-responsive (pH 6.8) negatively charged PPD copolymer was prepared according to our previous work [36]. The CA4P molecule and PPD were then stepwise absorbed or capped on the surface of PPQV via electrostatic adsorption, consequently fabricating the prodrug micellar nanosystem PPD/PPQV@C. The chemical shift and integration values of the characteristic peaks in the ^1^H nuclear magnetic resonance (NMR) spectrum (Fig. S1) confirmed the successful grafting of the target compound, as outlined in the experimental protocol. Gel permeation chromatography (GPC) analysis (Fig. S2) revealed that the molecular weights of various copolymers were consistent with its theoretical values, further supporting the successful synthesis of the PPD/PPQV@C. Additionally, Fourier transform infrared (FTIR) spectroscopy further confirmed the formation of the desired product through typical absorption bands of functional groups (Fig. S3), confirming again the formation of the desired products.

**Fig. 2.**
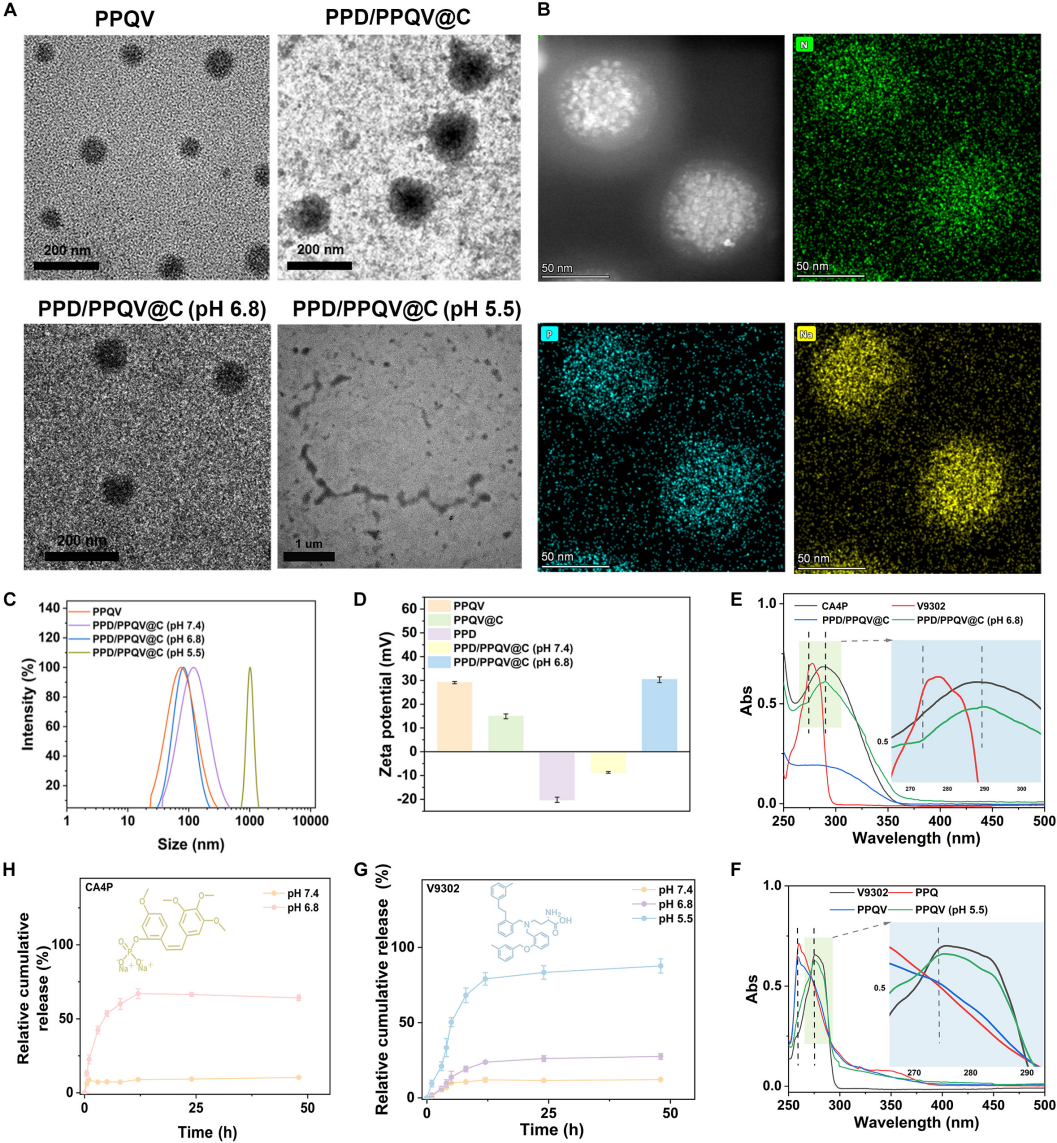
Characterizations of the PPD/PPQV@C micelle nanosystem. (A) TEM images of PPQV and PPD/PPQV@C after treatment with pH 7.4, 6.8, and 5.5. (B) EDS diagram of PPD/PPQV@C. (C) DLS and (D) zeta potential plots of PPD/PPQV@C after different treatments. (E and F) UV–vis spectra of CA4P, V9302, and their loaded/conjugated micelle nanosystem after different treatments. (G and H) Cumulative amount of V9302 and CA4P released from PPD/PPQV@C at different pH. Error bars represent mean ± SD (*n* = 3 independent samples).

Subsequently, the physicochemical properties of the micelle formed by above copolymers, including morphology, structure, size, surface charge, and drug loading, were characterized by transmission electron microscopy (TEM), dynamic light scattering (DLS), zeta potential measurement and energy-dispersive spectrometer (EDS). As displayed in Fig. 2A, both PPQV and PPD/PPQV@C possessed a relatively uniform and dispersed spherical structure, suggesting the formation of micelles. Furthermore, the characterized elements of P and Na were clearly observed on the PPD/PPQV@C micelle (Fig. 2B and Fig. S4), suggesting the successfully adsorption of CA4P and the construction of PPD/PPQV@C. More importantly, after coating with PPD outer layer, a distinct shell appeared on the surface of PPD/PPQV@C, and its diameter increased dramatically from about 55 nm to 120 nm, which was consistent with DLS analysis (Fig. 2C), proving the successful introduction of PPD and synthesis of the PPD/PPQV@C nanosystem. Last but not least, zeta potential data suggested that after the negatively charged CA4P and PPD were adsorbed to the surface of PPQV, the charges of PPQV@C and PPD/PPQV@C decreased significantly as expected (Fig. 2D), which reconfirmed the successful construction of the PPD/PPQV@C nanosystem. Notably, the characteristic peaks of V9302 and CA4P at 274 and 290 nm, respectively, were clearly observed on the absorption spectrum of PPD/PPQV@C (Fig. 2E) with the weak intensity, due to the complete conjugation of V9302 in the hydrophilic core of micelle nanosystem and adsorption of CA4P and actually shielding effect of PPD outer shell, indicating the successful encapsulation of above drugs and fabrication of PPD/PPQV@C. The loading content and loading efficiency of V9302 and CA4P were calculated as 13.22% and 40.35%, and 8% and 40.17%, respectively, according to the related standard curves (Fig. S5). Besides, the critical micelle concentration (CMC) of the nanosystem was measured as 35.28 μg/ml using the pyrene fluorescent probe method (Fig. S6). This low CMC value was helpful for resisting the dilution effect in blood circulation, indicating the good biostability.

After co-incubation with pH 6.8 phosphate-buffered saline (PBS) (simulation of the weakly acidic tumor microenvironment) for 12 h, the previous outer shell on PPD/PPQV@C was peeled off and reduced in size to about that of PPQV (Fig. 2A), which was consistent with the DLS data (Fig. 2C), and was accompanied by a shift in surface charge from negative to positive charge (Fig. 2D), directly confirming the pH-responsive size reduction and charge reversal capability of the nanosystem. Further, the original spherical morphology of PPD/PPQV@C underwent significant disintegration in response to a lower pH of 5.5 (mimicking lysosomal condition; Fig. 2A and C), implying the pH-sensitive prodrug release and micelle disassembly. To further quantitatively study drug release behavior, the real-time drug leakage dose was measured using the ultraviolet–visible (UV–vis) absorption spectroscopy. As shown in Fig. 2E and F, CA4P and V9302 were significantly released after 6 h of co-incubation with PBS at pH 6.8 and 5.5, manifested by a drastic increase in the intensity of characteristic absorption peaks, which was explained by the removal of PPD triggered by pH 6.8-activated electrostatic repulsion between PPD and PPQV and the breaking of the Schiff base linkage between PPQ and V9302. The real-time drug release curve demonstrated that the cumulative release rate of the 2 drugs (around 10%) was negligible after 24 h of incubation at pH 7.4, indicating the good drug encapsulation and stability of PPD/PPQV@C under physiological condition. The cumulative release rate of V9302 reached 40% and 80% at pH 6.8 and pH 5.5 (Fig. 2G), and CA4P nearly 70% at pH 6.8 (Fig. 2H), suggesting the nearly completed release of CA4P and V9302 in the tumor microenvironment and intracellular tumors (Fig. S7), which directly suggested the fractional release features. Besides, the PPD/PPQV@C nanosystem exhibited a good serum stability at pH 7.4 and pH 6.8 upon 10% fetal bovine serum (FBS) incubation over 6 d, as revealed by the negligible size change (Fig. S8). It was attributed to the effective shielding effect of PPD shell on the surface of the PPD/PPQV@C nanosystem. These results demonstrated that the PPD/PPQV@C nanosystem with good biostability possessed weakly acidic tumor microenvironment-responsive charge conversion and size reduction transformation and graded drug release features, which were beneficial for the achievement of the smooth arrival of the nanosystem at the tumor site, cargo release in the cytoplasm of tumor cells and ECM, and the full exertion of antitumor effects.

### Tumor penetration, uptake, and lysosomal escape of PPD/PPQV@C nanosystem

**Fig. 3.**
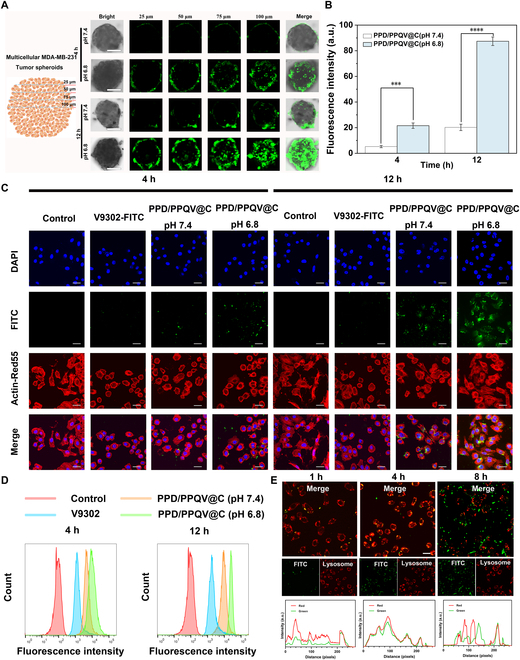
The evaluation of the PPD/PPQV@C micelle nanosystem on tumor penetration, endocytosis, and lysosomal escape effects. (A) CLSM and (B) fluorescence quantitative analysis of FITC-labeled PPD/PPQV@C micelles in MDA-MB-231 MCSs after culture at pH 7.4 and 6.8 for 4 and 12 h, respectively. (C) Endocytosis and (D) fluorescence quantitative analysis of FITC-labeled PPD/PPQV@C micelles in MDA-MB-231 cells after different treatments. (E) Lysosome escape behavior of FITC-labeled PPD/PPQV@C micelles in MDA-MB-231 cells after 1, 4, and 8 h of incubation. Scale bars, 50 μm (A) and 15 μm (C and E). Error bars represent mean ± SD (*n* = 4 biologically independent samples). The *P* values were determined by one-way ANOVA. ***P < 0.001.

The efficient delivery was the key factor for the ideal delivery nanosystem. In vivo, drug delivery to tumor cells by the nanosystem generally needs to cross the bio-barriers of tumor tissue, cell uptake, and lysosomal escape so that they could be released in situ and exert good tumor killing effects. The procedural overcoming of the above barriers caused by PPD/PPQV@C should be examined in detail. Three-dimensional multicellular tumor spheroids (MCSs) based on MDA-MB-231 cells were fabricated to evaluate the tumor penetration ability of the PPD/PPQV@C nanosystem firstly. As shown in Fig. 3A, the fluorescein isothiocyanate (FITC)-labeled PPD/PPQV@C was mainly dispersed on the outer layer of MCSs after 4 h of co-incubation at pH 7.4. After 12 h of incubation, only a small fraction of the PPD/PPQV@C had entered the MCSs, due to the relatively large size limitation of PPD/PPQV@C [38]. It was worth mentioning that PPD/PPQV@C could be obviously distributed inside the spheroids when the pH was adjusted to 6.8 under the same conditions regardless of incubation time, as manifested by a more extensive, bright, and clear distribution of green fluorescence throughout the MCSs. It was consistent with the corresponding fluorescence quantification analysis (Fig. 3B). The reason could be illustrated that the ionization of DMMA in the PPD shell induced the shedding of the PPD outer shell from the micelles and size reduction of the micelles; in response to the slightly acidic tumor microenvironment, the resultant PPQV with about 50 nm could preferentially penetrate deep into the MCSs and significantly improve the delivery efficiency of cargoes. These results directly confirmed that the PPD/PPQV@C nanosystem had good tumor penetration advantage.

Subsequently, the uptake levels of PPD/PPQV@C micelle in MDA-MB-231 cells were observed separately by confocal laser scanning microscopy (CLSM) and flow cytometry (FCM). As shown in Fig. 3C, the green fluorescence intensity of intracellular PPD/PPQV@C was significantly higher than that of the free V9302 despite incubation time, indicating the improved uptake efficiency of the nanosystem. Regardless of whether the incubation time was 4 or 12 h, the higher uptake amount of PPD/PPQV@C was displayed on the pH 6.8 treatment group than on the pH 7.4 treatment group (Fig. S9), as reflected in the brighter green fluorescence, which was further confirmed by the quantitative FCM statistics (Fig. 3D). At the same time, high-performance liquid chromatography was also used to detect the relative content of intracellular V9302 accumulation delivered by the nanosystem (Fig. S10), confirming again the enhanced nanosystem uptake by tumor cells. The enhanced uptake could be attributed to both the charge inversion and size reduction transformation of the PPD/PPQV@C nanosystem in response to the slightly acidic stimulation of tumor microenvironment, which had been confirmed by previous studies [39–41]. These results confirmed that PPD/PPQV@C micelles possessed pH-charge reversal property, which could significantly improve the effectiveness of drug delivery and bioavailability of cargoes.

Lysosomal escape effectiveness at the subcellular level was further examined by CLSM, which was directly associated with the exertion of V9302 bioavailability. As shown in Fig. 3E, after 1 h of co-incubation, a small amount of FITC-labeled PPD/PPQV@C was located at the edge of the cell membrane, which was a natural indicator of endocytosis; when the incubation time was extended to 4 h, most of the PPD/PPQV@C was co-localized with the lysosomes and exhibited the abundant yellow fluorescence; when extended to 8 h, PPD/PPQV@C labeled with green fluorescence had significantly transferred from the red fluorescently labeled lysosomes to the cytoplasm, indicating the successful lysosome escape. This escape was facilitated by the intrinsic protonation properties of PLL in PPQV@C in response to the acidic lysosomal environment. The above tendency was further confirmed by the related colocalization curve (Fig. 3E). These results confirmed that PPD/PPQV@C micelles were successfully functionalized with pH-responsive size reduction and charge reversal and protonation capabilities, which could effectively overcome the bio-barriers of tumor tissue, cellular uptake, and lysosomal escape, consequently leading to the potential enhancement of antitumor efficacy.

### In vitro cytotoxicity, cell apoptosis, and mitochondrial damage evaluation

**Fig. 4.**
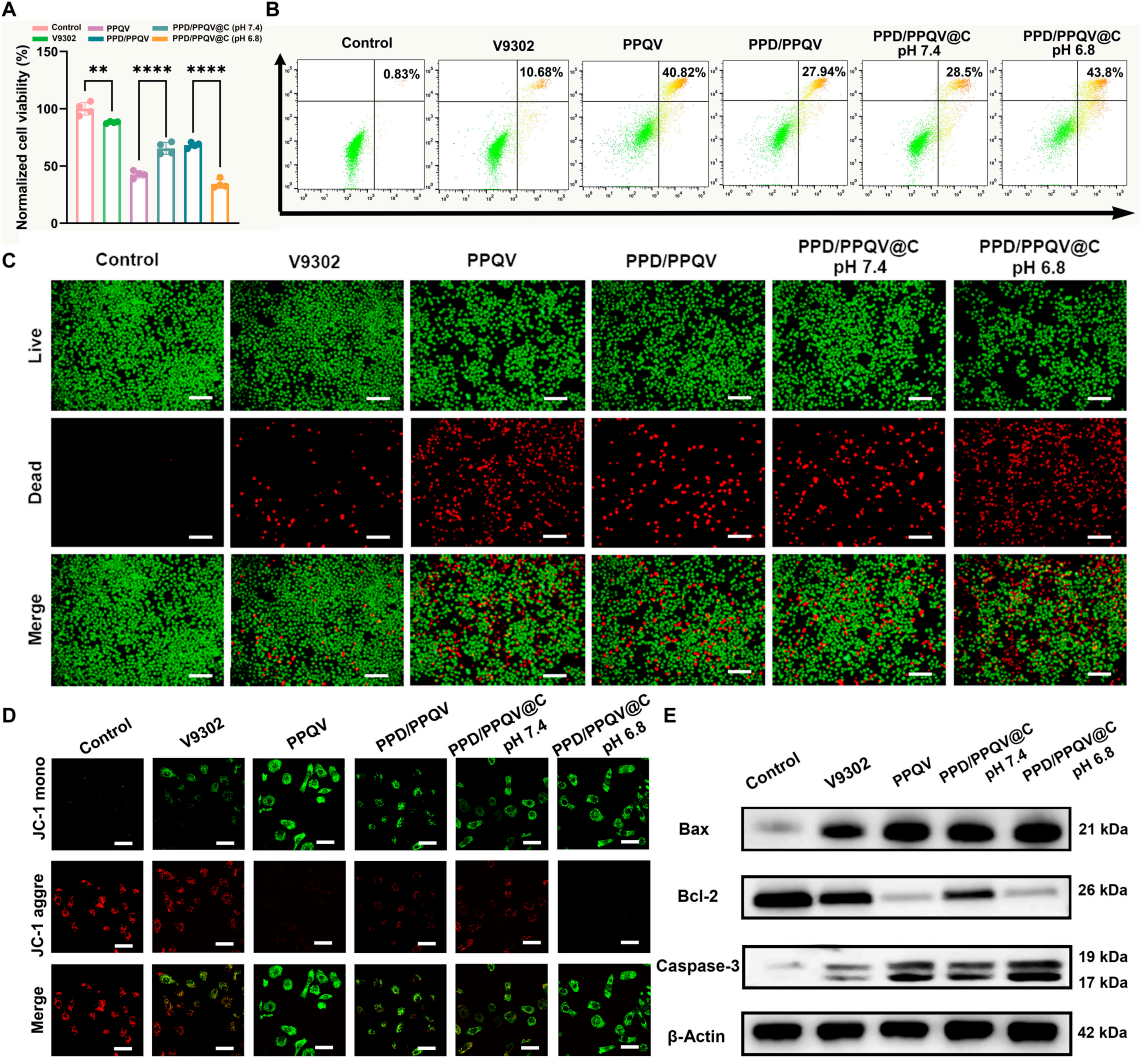
In vitro cytotoxicity, cell apoptosis, and mitochondrial damage evaluation. (A) Cytotoxicity and (B) apoptosis analysis of MDA-MB-231 cells after administration with different treatment groups for 24 h. (C) Live/dead cell staining of MDA-MB-231 cells under different treatments for 12 h. The green signal indicated the live cells, while the red indicated the dead cells. (D) Relative fluorescence analysis of JC-1 probes in mitochondrial of MDA-MB-231 cells after 12 h of administration. (E) Expression of apoptosis-related proteins in MDA-MB-231 cells after different treatments for 24 h, detected by Western blotting. Scale bars, 200 μm (C) and 15 μm (D). Error bars represent mean ± SD (*n* = 4 biologically independent samples). The *P* values were determined by one-way ANOVA. **P < 0.01, ***P < 0.001.

The in vitro antitumor effect of PPD/PPQV@C was then comprehensively evaluated. Firstly, the toxicity of the nanosystem to cells was investigated using the Cell Counting Kit-8 (CCK-8). As shown in Fig. S11, free V9302 exhibited a dose-dependent toxicity to MDA-MB-231 cells after 24 h of incubation, due to its natural cytotoxicity by blocking glutamine metabolism [22]. We subsequently investigated the cytotoxicity among the treatment groups (Fig. 4A), showing an order trend of V9302 < PPD/PPQV or PPD/PPQV@C (pH 7.4) < PPQV or PPD/PPQV@C (pH 6.8). Combining the positive charge property and the strategy of nanocarrier, both the prodrug micelle PPQV and PPD/PPQV@C under pH 6.8 incubation condition with the superior delivery efficiency induced stronger cytotoxicity than V9302 alone. Meanwhile, the cell viability of the PPD/PPQV and PPD/PPQV@C (pH 7.4) treatment groups was higher than that of the noncoated PPQV and PPD-removed PPD/PPQV@C (pH 6.8), due to the charge masking effect. Notably, the loading of CA4P or its absence had no significant effect on the activity of tumor cells in the absence of endothelial cell involvement, since its main role was to block angiogenesis. Further, the trend of cytotoxicity caused by the different treatment groups described above was similarly presented in the apoptosis analysis of MDA-MB-231 cells, using Annexin V-FITC/propidium iodide (PI) staining (Fig. 4B and Fig. S12) and live/dead staining assays (Fig. 4C), and both the prodrug micelle PPQV and resultant PPD/PPQV@C upon pH 6.8 (simulating the tumor microenvironment) exhibited the highest apoptosis rate of tumor cells (~43%; Fig. S12), after 24 h of incubation without endothelial cells, confirming again the tumor microenvironment-activated good in vitro antitumor effect of the PPD/PPQV@C nanosystem.

Mitochondrial damage or depolarization is a common manifestation of apoptosis [42]. The JC-1 fluorescent probe was subsequently used to measure the damage level of mitochondrial damage or depolarization of the PPD/PPQV@C nanosystem. In general, JC-1 aggregates in normal mitochondria and emits red fluorescence, while dispersing in the cytoplasm and displaying green fluorescence upon mitochondrial damage. As expected, V9302 generated the weakly detectable green fluorescence, indicating slight mitochondrial damage (Fig. 4D and Fig. S13), while the strongest green fluorescence and weakest red fluorescence were displayed on the PPD/PPQV@C nanosystem upon pH 6.8 (simulating tumor weakly acidic microenvironment), similar to PPQV micelles alone, suggesting the effective induction of mitochondrial damage. Furthermore, after 24 h of incubation, the expression of these typical apoptotic factors located downstream of mitochondrial damage was most highly up-regulated on the PPD/PPQV@C upon pH 6.8 and PPQV treatment groups, including the ratio of Bax/Bcl-2 and caspase-3 (Fig. 4E and Fig. S14), indicating the activation of apoptosis pathways based on the mitochondrial damage.

### Nanosystem interferes with ASCT2 channels, depriving glutamine to induce starvation of tumor cells

**Fig. 5.**
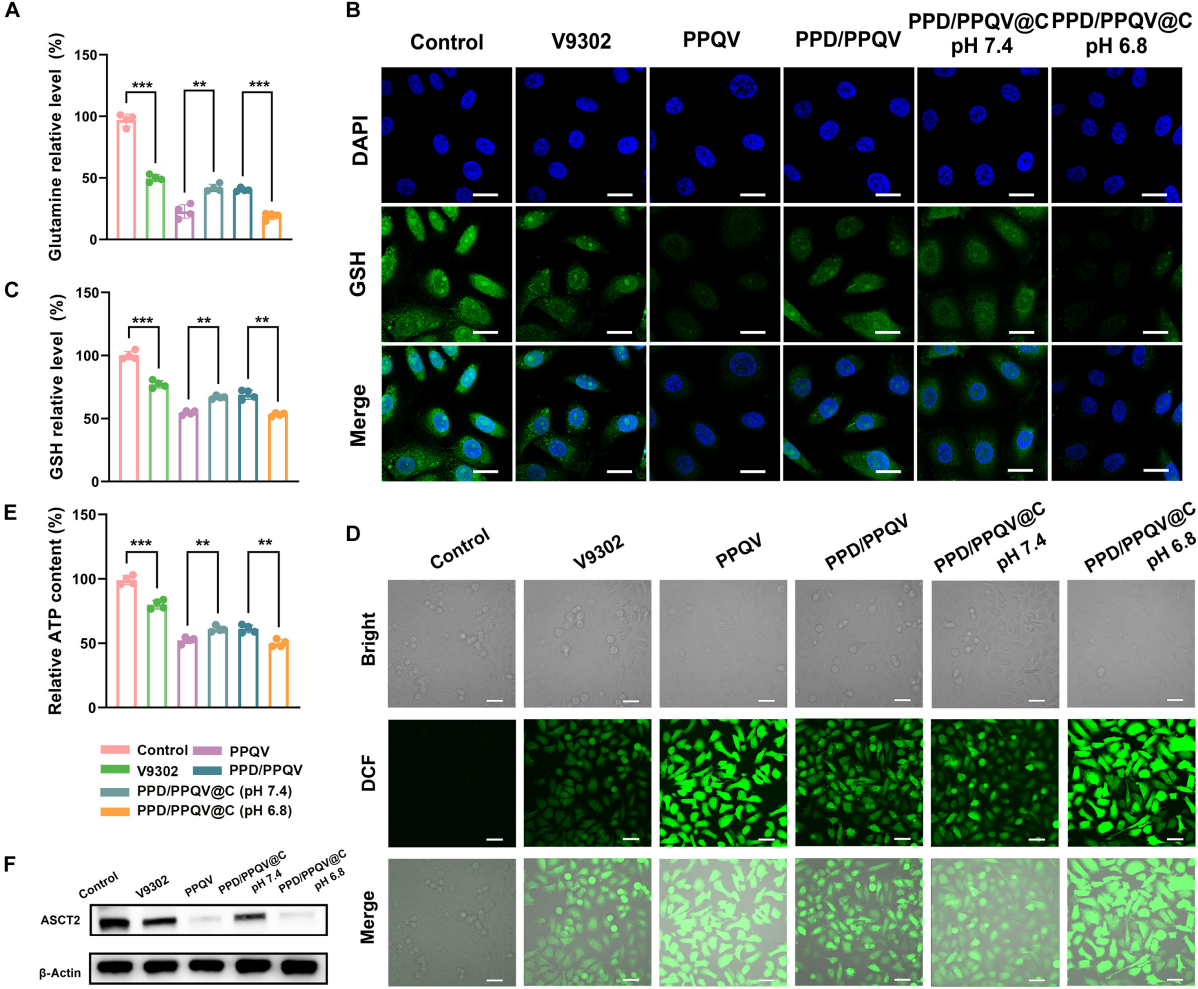
Tumor killing mechanism evaluation in vitro of the nanosystem. (A) Determination of intracellular glutamine content. (B) CLSM imaging of intracellular GSH under different treatments. (C) Intracellular GSH content after different treatments for 24 h. (D) CLSM images of intracellular ROS in MDA-MB-231 cells after different treatments for 12 h. (E) Intracellular ATP levels in MDA-MB-231 cells after 24 h of co-incubation with different treatment groups. (F) Expression of ASCT2 proteins in MDA-MB-231 cells after different treatments for 24 h, detected by Western blotting. Scale bars, 20 μm (B) and 40 μm (D). Error bars represent mean ± SD (*n* = 4 biologically independent samples). The *P* values were determined by one-way ANOVA. **P < 0.01, ***P < 0.001.

Subsequently, we investigated the mechanism of apoptosis induced by the nanosystem. As shown in Fig. 5A, intracellular glutamine content was measured by a glutamine kit, due to the fact that V9302 can inhibit ASCT2 and thus make it difficult for glutamine to enter the cell, showing an order trend of V9302 < PPD/PPQV or PPD/PPQV@C (pH 7.4) < PPQV or PPD/PPQV@C (pH 6.8). This is due to the excellent delivery efficiency of the drug delivery system. Then, GSH depletion and oxidative stress were investigated, considering that the inhibition of glutamine metabolism mediated by the V9302 in the nanosystem directly interferes with the synthesis of GSH [43], which could induce tumor apoptosis/death by amplifying oxidative stress in tumor cells. Compared to control, free V9302 generated the moderated reduction of intracellular GSH content, shown by the decreased fluorescence intensity of GSH probe and GSH kit (Fig. 5B and C), which was directly attributed to the entry blockade of glutamine into cells. Further, the reduction of GSH by the nanosystem exhibited a time-dependent behavior (Fig. S15). With the help of nanocarriers based on prodrug micelles, PPQV, PPD/PPQV, and PPD/PPQV@C all induced weaker GSH synthesis than free V9302. Notably, the PPD/PPQV@C nanosystem exhibited the lowest levels of GSH similar to PPQV micelles alone when exposed to pH 6.8 (simulating tumor weakly acidic microenvironment), due to the removal of the PPD shell and exposure of PPQV micelles with smaller size and positive charge. Glutamine, an important raw material for GSH synthesis, was effectively inhibited by intracellular transport to the greatest extent (Fig. 5C). Further, the intracellular reactive oxygen species (ROS) levels rose sharply with the massive depletion of GSH, as manifested by the brightly green fluorescence and the related quantitative statistics using the 2,7-dichlorodihydrofluorescein diacetate (DCFH-DA) probe (Fig. 5D and Fig. S16), consequently leading to the highest oxidative stress in the tumor cells.

In addition, the above PPD/PPQV@C nanosystem could also significantly suppress adenosine triphosphate (ATP) generation (Fig. 5E), due to TNBC-dependent OXPHOS and the tricarboxylic acid (TCA) cycle being blocked at lower intracellular glutamine conditions, which was achieved by the inhibition of intracellular glutamine transport via ASCT2 (Fig. 5F). As a result, glutamine was difficult to metabolize into α-ketoglutarate (α-KG), so it could not enter the TCA cycle and produce ATP [44]. The decrease in ATP was due to the reduction of glutamine uptake caused by V9302 and V9302-loaded groups [PPD/PPQV and PDD/PPQV@C (pH 6.8 and pH 7.4)]. Notably, since no blood vessels were involved, the ATP reduction of MDA-MB-231 cells in both PPQV and PDD/PPQV@C (pH 6.8) groups was mainly due to V9302 rather than CA4P, because CA4P targeting endothelial cells within tumor tissues selectively disrupts tumor vasculature, effectively inhibiting oxygen and nutrient transfer, and ultimately suppressing tumor energy supply [45]. Thus, the above 2 groups exhibited a similar inhibitory effect on ATP. The excellent tumor killing/apoptosis induced by the PPD/PPQV@C nanosystem in the absence of endothelial cell involvement could be explained as follows: due to the inhibition of the most potent glutamine transport, as evidenced by the highest inhibitory expression of ASCT2 transporter and the lowest level of intracellular glutamine content, resulting in a dramatic reinforcement in intracellular oxidative stress through GSH depletion and increased ROS, ultimately activating the apoptosis/death pathway by inducing mitochondrial damage and generating the good tumor killing effect in vivo*.*

### Cellular metabolomics analysis

**Fig. 6.**
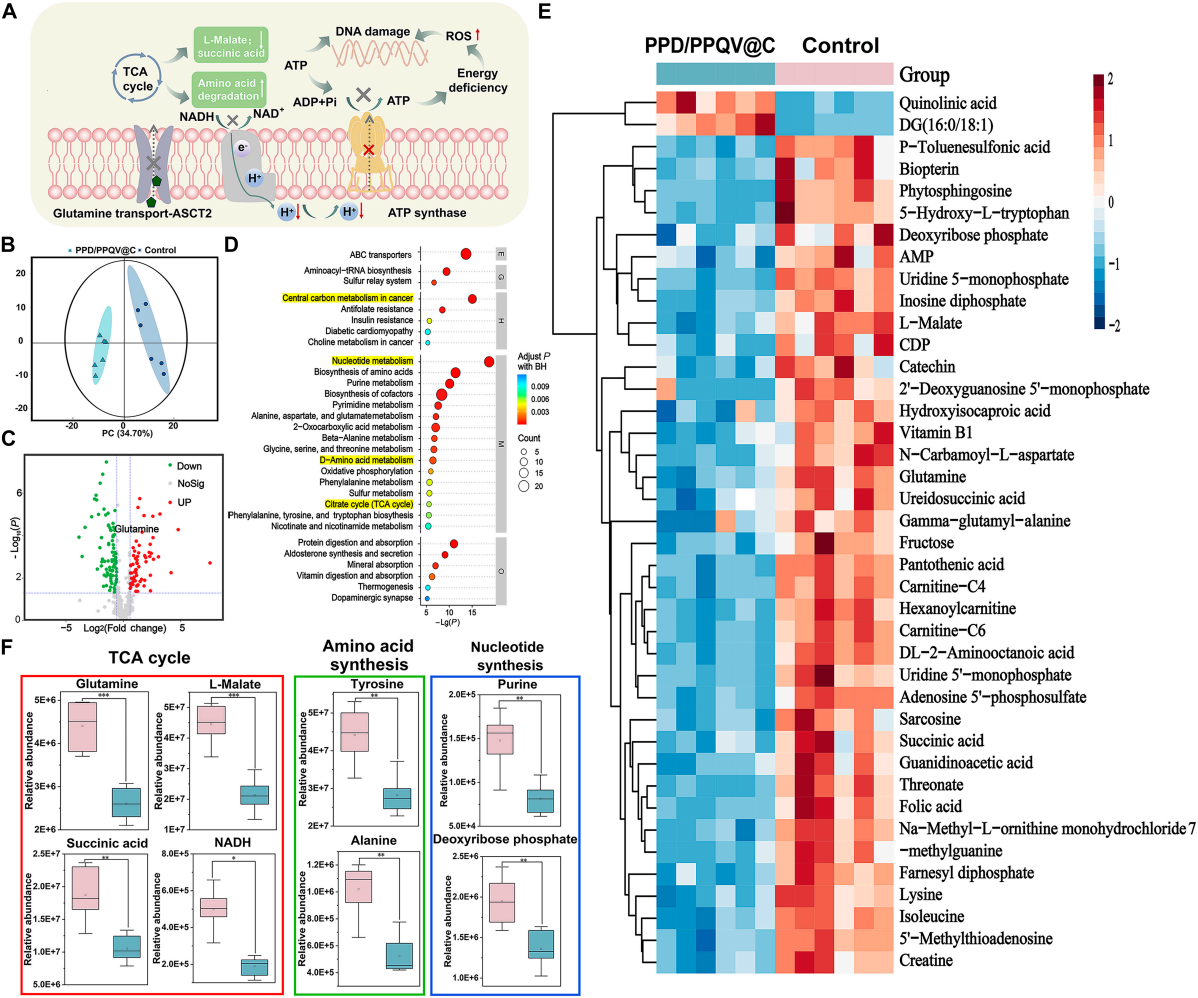
Metabolomics analysis of MDA-MB-231 cells upon nanosystem treatment. (A) Schematic diagram of the energy metabolism mechanism of tumor cells. (B) PCA of MDA-MB-231 cells. (C) Expression of differential genes in MDA-MB-231 cells after 24 h of incubation with the nanosystem. (D) KEGG pathway enrichment analysis. (E) Heatmap of differential gene expression in MDA-MB-231 cells treated with PBS and PPD/PPQV@C. (F) Quantitative analysis of intracellular metabolite changes in the TCA cycle, biomacromolecules, and nucleotide synthesis pathways in MDA-MB-231 cells after the above treatments. Error bars represent mean ± SD (*n* = 6 biologically independent samples). The *P* values were determined by one-way ANOVA. *P < 0.05, **P < 0.01, ***P < 0.001.

In order to further clarify the inhibitory effect of the PPD/PPQV@C nanosystem on breast cancer-dependent glutamine metabolism and the related other metabolisms, the targeted metabolomics study was performed after incubation of the PPD/PPQV@C nanosystem with MDA-MB-231 cells for 24 h. Figure 6A illustrates a schematic diagram of the mechanism of disruption of energy metabolism in MDA-MB-231 cells by interfering with glutamine intake. As demonstrated by principal components analysis (PCA; Fig. 6B), the clustering characteristics of PPD/PPQV@C were different from control, and the clustering dispersion of the 2 cell samples was relatively small, indicating that the metabolomics analysis had good stability and validity. Simultaneously, the metabolites of the cancer cells treated by the PPD/PPQV@C group were significantly different compared to control (Fig. 6C), implying the exact occurrence of metabolic regulation. Further, the Kyoto Encyclopedia of Genes and Genomes (KEGG) enrichment analysis highlighted that the differential metabolites caused by PPD/PPQV@C were mainly involved in the cancer cell-dependent amino acid biosynthesis, central carbon metabolism, and the TCA cycle process (Fig. 6D), suggesting that PPD/PPQV@C could actually starve tumor cells. Moreover, the heatmap analysis strongly supported that PPD/PPQV@C treatment could obviously interfere with glutamine, the related amino acid metabolism, nucleotide metabolism, and TCA cycle, indicating the effective suppression of glutamine metabolism and its related other substance or energy metabolisms (Fig. 6E).

Specifically, breast cells mainly uptake extracellular glutamine via the ASCT2/SLC1A5 transporter, a process critical for synthesizing the nucleotides and amino acids required for rapid growth [46]. Notably, glutamine could also convert to α-KG by aspartate aminotransferases (TAs) or glutamate dehydrogenase (GDH) or alanine and participate in TCA cycle to provide energy to tumor cells [47,48]. As shown by the quantitative analysis (Fig. 6F), the content of glutamine in the PPD/PPQV@C group-treated MDA-MB-231 was significantly reduced, suggesting again the inhibition of glutamine metabolism. The glutamine content of MDA-MB-231 treated with the PPD/PPQV@C treatment group was significantly reduced, again indicating that glutamine metabolism was inhibited. In addition, the concentrations of purine deoxyribose and deoxyribose phosphate, which are known to be involved in nucleotide synthesis and metabolism, were also significantly reduced [49] and the nonessential amino acids alanine and tyrosine were also reduced, indicating that the nutrients required for the metabolism of important substances such as nucleotides and proteins were significantly reduced. Moreover, the content of the typical products in TCA cycle, e.g., succinic acid, l-malate, and NADH [reduced form of nicotinamide adenine dinucleotide (oxidized form)], was significantly reduced in PPD/PPQV@C administration, which further confirmed the inhibition occurrence of glutamine metabolism and the energy loss. These results collectively confirmed that PPD/PPQV@C could effectively block glutamine metabolism and inhibit the dependent nucleotide synthesis, amino acid metabolism, and energy supply of tumor cells, which was helpful for the enhancement of TNBC therapy.

### The biosafety and anti-angiogenesis in vitro of nanosystem on endothelial cells

**Fig. 7.**
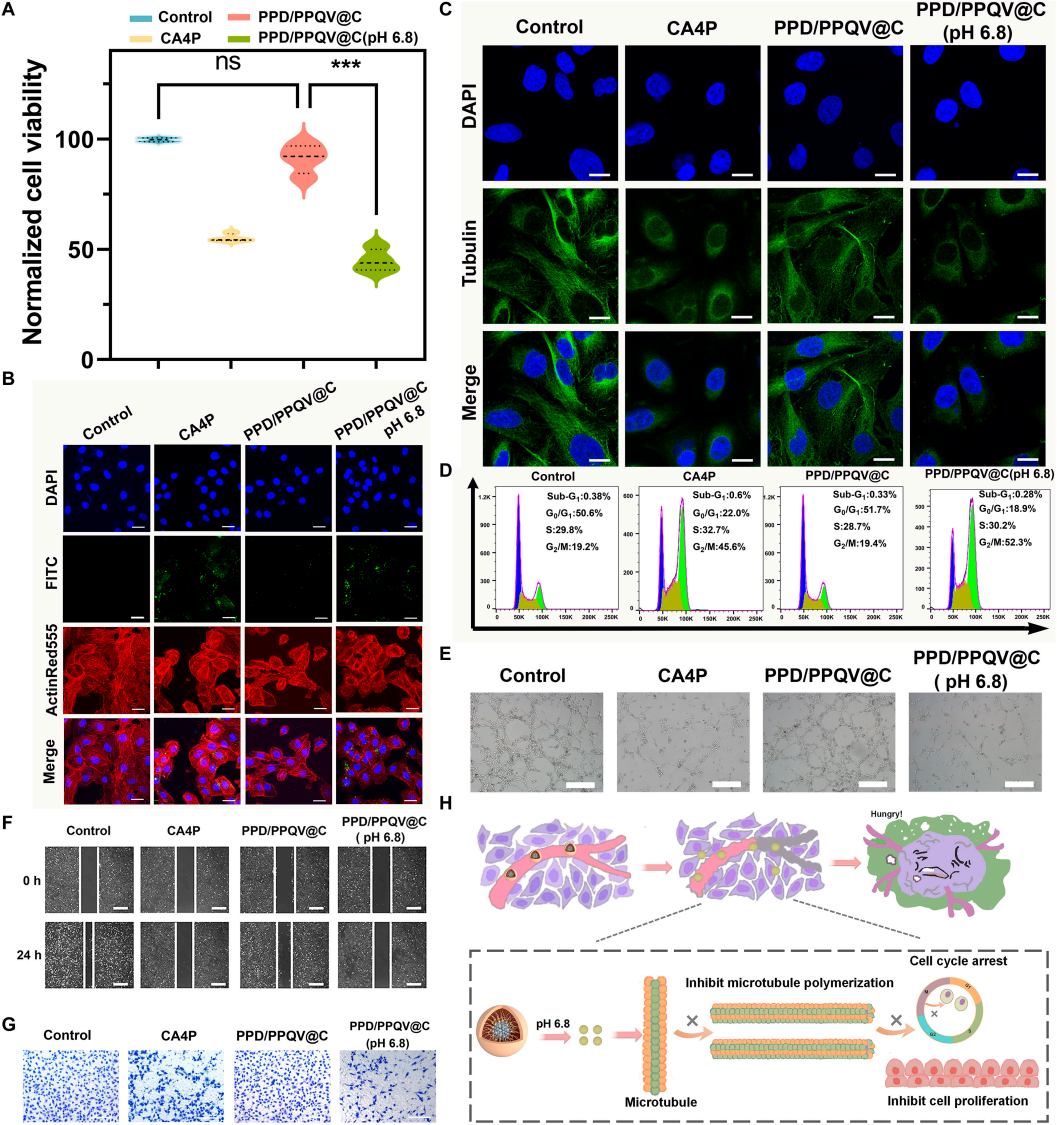
In vitro anti-angiogenesis studies of the nanosystem. (A) Cytotoxicity of HUVECs after 24 h of incubation with different treatment groups. (B) CLSM analysis of HUVECs after incubation with FITC-labeled CA4P or PPD/PPQV@C micelles at pH 7.4 and 6.8 for 8 h, respectively. (C) CLSM analysis of microtubule formation in HUVECs after different treatments. (D) Cell cycle of HUVECs after different administration. (E) Microscopic images of HUVEC vascularization, (F) scratches, and (G) invasion after above treatments. (H) Schematic illustration of the anti-vascular mechanism of the PPD/PPQV@C nanosystem. Scale bars, 15 μm (B and C), 450 μm (E), 300 μm (F), and 150 μm (G). Error bars represent mean ± SD (*n* = 4 biologically independent samples). The *P* values were determined by one-way ANOVA. ***P < 0.001.

The V9302-grafted PPD/PPQV@C nanosystem has been shown to be effective in killing tumors in vitro and revealing the relevant mechanism by directly blocking glutamine uptake and starvation tumors, and the role of loaded CA4P in regulating angiogenesis should be studied at the same time. The biosafety of the nanosystem was first assessed on the model cells of human umbilical cord vascular endothelial cells (HUVECs). The obvious proliferation suppression observed in the CA4P group (Fig. 7A) was attributed to its natural vascular toxicity [50]. Benefiting from the charge shielding effect imparted by the PPD outer polymer, PPD/PPQV@C was virtually nontoxic to HUVECs. It was further confirmed by the negligible uptake of PPD/PPQV@C labeled with FITC (Fig. 7B) after 8 h of incubation, suggesting good biosafety. When exposed to pH 6.8 (simulation tumor microenvironment), the uptake amount of CA4P labeled with FITC was significantly improved (Fig. S17), which was attributed to the rapid release of CA4P from the PPD/PPQV@C nanosystem. Notably, the cytoskeleton of the CA4P and CA4P-loaded PPD/PPQV@C under pH 7.4 showed significant disruption compared with control, which was probably attributed to the natural inhibitory effect of CA4P on tubulin [51]. These results suggested that the PPD/PPQV@C nanosystem functionalized with tumor microenvironment-sensitive peel-off PPD outer polymer and size/charge transformation could effectively avoid the nonspecific uptake by normal cells and enhance endocytosis of tumor cells, exhibiting good biosafety with improved tumor treatment effect.

In order to further clarify the actual work of loaded CA4P in regulating angiogenesis, the changes of microtubules stage and morphology in HUVECs were subsequently visualized with CLSM, after treatment with CA4P and CA4P-loaded administrations, considering its natural proliferation inhibition of endothelial cells by interfering tubulin and mitosis. Compared to control, CA4P could obviously disrupt tubulin aggregation of HUVECs (Fig. 7C), while almost no tubulin aggregation was presented in the PPD/PPQV@C treatment group, attributing to the weak uptake of HUVECs. When exposed to pH 6.8, significant microtubule aggregation occurred, as similar to CA4P alone, due to the responsive release of CA4P. This result directly demonstrated that the PPD/PPQV@C nanosystem could significantly release CA4P and activate the transformation of size/charge in response to the weakly acidic tumor microenvironment, and the released CA4P could effectively inhibit microtubule aggregation and induce morphological deformation of HUVECs, leading to the resultant disruption of the vascular network of solid tumors and anti-angiogenesis in vivo.

Further, the effect of CA4P loading groups on cell cycle of HUVECs was studied to elucidate its anti-angiogenesis mechanism. As displayed in Fig. 7D, both CA4P and PPD/PPQV@C (pH 6.8) generated obvious cell cycle arrest, which was due to the fact that CA4P could induce mitotic arrest at the G_2_/M phase by blocking tubulin and inhibiting spindle formation [52]. On the contrary, limited by negligible CA4P leakage and little nonspecific uptake of the nanosystem under normal physiological conditions (pH 7.4), the PPD/PPQV@C group had almost no effect on cell cycle arrest of HUVECs (Fig. S18), confirming again the good biosafety and inhibition of cell cycle of the nanosystem on endothelial cells.

Next, the tube formation, the movement of HUVECs related to wound healing, and invasion studies were run to visually illustrate in vitro anti-angiogenesis of the CA4P-loaded nanosystem. As shown in Fig. 7E and Fig. S19, both CA4P and CA4P-loaded PPD/PPQV@C upon pH 6.8 could significantly suppress the tube formation based on HUVECs, compared to control. The PPD/PPQV@C administration upon pH 7.4 had little significant effect on tube formation in HUVECs, attributing to the slight uptake. Moreover, the above suppression tendency of tube formation was displayed on the cell mobility assays, which was manifested in the significant inhibition of cell coverage at scratch (Fig. 7F and Fig. S20) and the obstruction of invasion to the lower chamber of the transwell in the free CA4P and CA4P-loaded groups at pH 6.8 (Fig. 7G and Fig. S21). Meanwhile, the inhibition intensity of CA4P-loaded groups at pH 6.8 was higher than that of free CA4P, which might be due to the low acid stimulation in the tumor microenvironment causing the removal of PPD outer polymer and the exposure of PPQV with positive charge characteristics, and the PPQV grafted with V9302 could be appropriately taken up by HUVECs and cause inhibition of movement and proliferation through the natural toxicity of V9302 (Fig. 7E and F and Figs. S19 and S20) [21]. Briefly, the good in vitro anti-angiogenic mechanism of the PPD/PPQV@C nanosystem was illustrated as follows (Fig. 7H): the outer polymer PPD was removed and the CA4P in the middle layer was fully released in response to the slightly acidic stimulation in the tumor microenvironment, and the released CA4P could effectively inhibit the microtubule polymerization stability of tumor-related vascular endothelial cells and deform their morphology, resulting in cell cycle arrest and inhibition of proliferation, thereby blocking tumor angiogenesis, which in turn would significantly block the supply of essential nutrients such as glucose and oxygen to tumors, further inhibiting compensatory metabolism and enhancing the injury caused by tumor starvation fundamentally. These experiments clearly demonstrated that the core function of CA4P in the nanosystem is to specifically inhibit angiogenesis by interfering with tubulin formation and cell cycle arrest of endothelial cells, which was selectively released in the ECM from the nanosystem with good biosafety.

### In vivo antitumor analysis of PPD/PPQV@C nanosystem

**Fig. 8.**
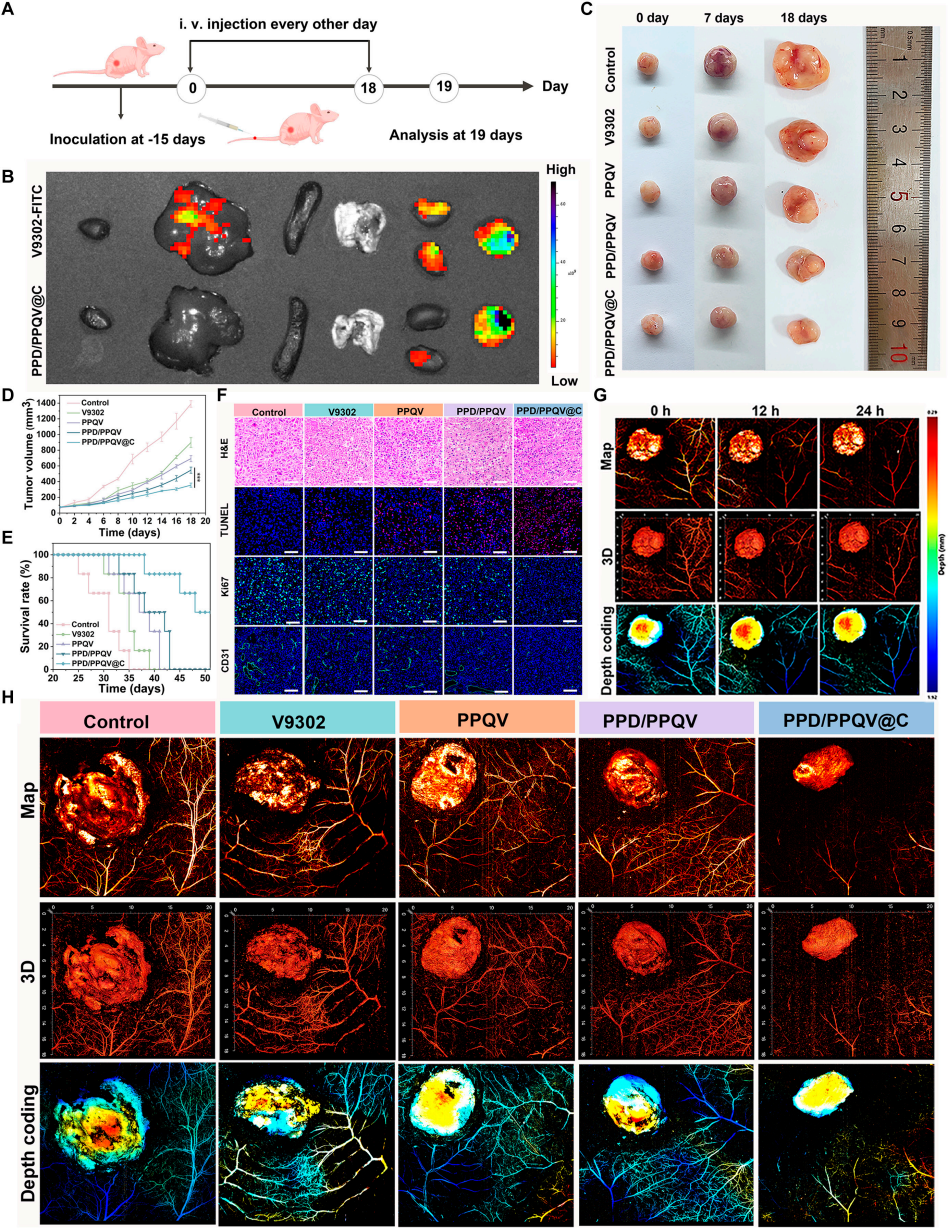
In vivo antitumor efficacy of PPD/PPQV@C. (A) Schematic diagram of in vivo administration. (B) Biodistribution of PPD/PPQV@C labeled with FITC in vivo in major tissues after 6 h of injection. (C) Representative images of tumors at 0, 7, and 18 d of treatments. (D) Mean tumor growth curves and (E) survival time of mice in different groups. (F) Immunofluorescence images of H&E, TUNEL, Ki67, and CD31 for tumors. (G) Vascular photographs upon 24 h after dosing with the PPD/PPQV@C nanosystem for tumors and its surrounding blood vessels. (H) Photographs of vessel density and abundance for tumor tissue and its surrounding blood vessels in different administration after 7 d of treatment. Error bars represent mean ± SD (*n* = 6 biologically independent samples). The *P* values were determined by one-way ANOVA. ***P < 0.001.

The in vivo antitumor efficacy of the PPD/PPQV@C nanosystem was explored in an MDA-MB-231 cell-bearing BALB/c nude mouse model. The treatment regimen was presented in Fig. 8A. At first, the biosafety of the PPD/PPQV@C nanosystem was evaluated. Compared with the control group (saline), the PPD/PPQV@C administration had no effect on blood biochemical levels (Fig. S22) and liver and kidney function indexes of tumor-bearing mice after 7 d of treatment, indicating good biocompatibility in vivo. Meanwhile, the PPD/PPQV@C group also did not significantly cause weight loss and damage to major organs during the treatment period relative to the control group (Figs. S23 and S24), confirming again the good biosafety. Additionally, after 6 h of injection, the biodistribution of the nanosystem study further demonstrated that the PPD/PPQV@C labeled with FITC mainly gathered at the tumor lesions, and it also significantly reduced the accumulation of drug in the liver and kidney (Fig. 8B and Fig. S25), compared with the free V9302 group, revealing good tumor targeting in vivo with reduced side effect. The reason could be understood that due to the PPD shell-mediated charge masking effect and pH-responsive programmed size/charge transition strategies, the PPD/PPQV@C nanosystem could effectively avoid the uptake of the reticuloendothelial system (RES) and efficiently accumulate in tumor lesions by overcoming the tumor penetration and cellular uptake barriers [53], thus exhibiting the good biosafety and tumor accumulation in vivo, which are the prerequisites for effective tumor killing.

Subsequently, the size and real-time change of tumor volume were measured to directly illustrate the tumor growth suppression efficiency of the PPD/PPQV@C nanosystem. As exhibited in Fig. 8C, the PPQV group exhibited stronger tumor growth inhibition than the free V9302, due to the better delivery efficiency of prodrug micelle and the higher antitumor efficacy displayed by PPD/PPQV than PPQV, attributed to the reduced nonspecific endocytosis mediated by the introduction of PPD outer polymer and enhanced uptake by tumors via the pH-activated size reduction and charge reversion; benefiting from the introduction of the anti-vascular drug CA4P, PPD/PPQV@C showed the strongest antitumor effect via the blockade of glutamine metabolism and angiogenesis suppression. The above suppression tendency of tumor growth was further confirmed by the real-time change of tumor volume (Fig. 8D and Fig. S26). Furthermore, the PPD/PPQV@C nanosystem combined the hierarchical delivery and tumor suppression with blockade of glutamine metabolism and angiogenesis suppression could significantly extend the survival time of tumor-bearing mice to 51 d with the highest level of survival rate among all administrations (Fig. 8E), revealing the superior antitumor effect in vivo of the PPD/PPQV@C nanosystem. In addition, the results from the tissue level of hematoxylin and eosin (H&E) staining, targeting Ki67 and terminal deoxynucleotidyl transferase-mediated deoxyuridine triphosphate nick end labeling (TUNEL) immunofluorescence staining of tumor sections in the above treatment groups, showed that the PPD/PPQV@C group induced the most severe tumor tissue destruction, which was manifested in the obvious tissue structure breakage and nuclear consolidation, a large number of pink fluorescent dots representing DNA damage colocated on the nucleus, and a significant reduction in the green fluorescent labeling of Ki67 cell proliferation markers (Fig. 8F and Fig. S27), further demonstrating the advanced antitumor effect of PPD/PPQV@C.

Last but not least, the anti-vascular performance in vivo was investigated to further reveal the overall antitumor effect of the PPD/PPQV@C nanosystem, as it is directly related to the exact blockade of compensatory metabolism fundamentally and the achievement of tumor suppression. Briefly, the density and intensity of blood vessels around the tumor in mice were observed using photoacoustic multimode small animal in vivo imaging system. The CA4P-loaded group PPD/PPQV@C significantly reduced the number and intensity of intratumoral and peritumoral blood vessels after 7 d of treatment (Fig. S28), indicating effective angiogenesis inhibition and nutrient supply blockade, which was helpful for accelerating tumor starvation effect. Furthermore, within 24 h of administration, a clearer time-dependent trend of decreasing tumor neovascularization density and intensity was presented in PPD/PPQV@C groups (Fig. 8G), confirming again the excellent antitumor angiogenesis performance. More importantly, different treatment groups were observed. In the control group, a large number of neovascularization were scattered around and within the tumor tissue, which were manifested by the clear vascular structure and the strong fluorescence intensity of the marked blood vessels (Fig. 8H and Fig. S29), which was required for its indefinite reproduction. Meanwhile, the tumor volume of treatment groups decreased to varying degrees compared with the control after 7 d of treatment, and the tumor size in the PPD/PPQV@C group was the smallest, which was consistent with the results of previous real-time tumor volume curve. It was suggested that the angiogenesis suppression could further enhance the glutamine metabolism blockade tumor therapy effect by directly cutting off the delivery of nutrients to tumors. In sum, the good antitumor effect of the nanosystem in vivo could be interpreted as follows: (a) the pH-response charge reversal and size reduction function of the PPD/PPQV@C nanosystem (Fig. 2) significantly enhanced its uptake efficiency by tumor cells (Fig. 3); (b) V9302 and CA4P were respectively delivered in the tumor intracellular and extracellular matrix through the graded release strategy of the nanosystem (Figs. 1 and 2G and H and Fig. S7), further improving delivery efficacy and bioavailability of above drugs; (c) V9302 accumulated at the cytoplasm of tumor cells could significantly limit glutamine uptake and disrupt downstream ATP, GSH generation, and ROS accumulation by blocking the ASCT2 transporter (Fig. 5), which also induced the severe mitochondrial dysfunction and activation of apoptotic pathways (Fig. 4); importantly, metabolomics further confirmed the suppression of glutamine-derived TCA cycle intermediates (e.g., α-KG and succinate) and nucleotide precursors, starving tumors of energy and biosynthetic substrates (Fig. 6); (d) CA4P released in the tumor microenvironment indeed disrupted angiogenesis by inhibiting tubulin polymerization of endothelial cells in vitro (Fig. 7), leading to vascular collapse and further nutrient deprivation in vivo (Fig. 8G and H); (e) lastly, this dual blockade of intracellular glutamine uptake and extracellular vascular angiogenesis exhibited the effectively enhanced tumor suppression with prolonged survival outcomes (Fig. 8).

## Conclusion

In conclusion, we designed a size/charge programmable micellar nanosystem endowed with staged delivery capability for TNBC starvation therapy with blockade of glutamine metabolism and angiogenesis. The fabricated PPD/PPQV@C nanosystem could reduce size and reverse charge to overcome biobarries of tumor penetration and cell uptake in response to the tumor acidic microenvironment, and rapidly release vascular blocker CA4P in the ECM. The resultant PPQV endocytosed by tumor cells could sensitively release the glutamine transportation inhibitor V9302 in situ, achieving the precise graded delivery with high bioavailability. CA4P and V9302, located at their respective sites of action, could effectively inhibit angiogenesis, block the nutrient supply for tumor cells, and cut off the uptake and metabolism of glutamine, leading to the superior starvation effect on TNBC. All in vitro and in vivo experiments collectively demonstrated that the PPD/PPQV@C nanosystem not only significantly improved the bioavailability of CA4P and V9302 via hierarchical delivery and pH-responsive size/charge transformation strategies, but also inhibited tumor growth with prolonged survival time through the dual blockade combined with the glutamine deprivation and angiogenesis inhibition, presenting a smart nanoplatform for the efficient TNBC therapy.

## Materials and Methods

### Materials

Hydroxy succinimide (NHS) was purchased from Sigma-Aldrich (USA). Chloroacetic acid, methoxy poly(ethylene glycol) amine (mPEG-NH_2_, molecular weight 5,000), d-mannose, and trifluoroacetic acid (TFA) were obtained from J&K Scientific Ltd. 2,3-Dimethylmaleic anhydride (DMMA) and polyethyleneimine were bought from Aladdin Industrial Co. Ltd. FBS was obtained from Biological Industries (USA). *N*,*N*-dimethylformamide (DMF) was obtained from Solarbio. The water utilized in all experiments was purified through a Millipore system. All the chemicals were employed in their received form and without the need for further purification.

### Synthesis of PEG-PLL

Typically, the synthesis of PEG-PLL was referred to our previous work [36]. ^1^H NMR (500 MHz, D_2_O) of Fig. S1A: 4.21 (s, 31H, -COCHNH-), 3.59 (s, 476H, -OCH2CH2O-), 3.32 (s, 3H, -OCH3), 2.90 (s, 62H, -NHCH2CH2-), 1.35 to 1.58 (d, 186H, -CHCH2CH2CH2CH2-). The GPC and FTIR spectra of PEG-PLL were displayed in Figs. S2 and S3, respectively.

### Synthesis of PPD copolymer

Briefly, the pH of PEG-PLL (100 mg) aqueous solution was adjusted to about 8.5 with sodium hydroxide (1 M). After that, DMMA (87 mg) dissolved in DMF (1 ml) was added dropwise to the above mixture and the value of pH was constantly kept at 8 to 9. The reaction was continued at room temperature for 12 to 24 h, followed by dialysis with double-distilled water for 72 h [molecular weight cutoff (MWCO) 3,500 Da]. The outer polymer was collected with a freeze drier and denoted as PPD. ^1^H NMR (500 MHz, D_2_O) of Fig. S1B: 4.23 (s, 31H, -COCHNH-), 3.63 (s, 475H, -OCH_2_CH_2_O-), 3.31 (s, 3H, -OCH3), 3.09 (s, 42H, -NHCH_2_CH_2_-), 2.90 (s, 22H, NH_2_CH_2_CH_2_-), 1.70 to 1.85 (m, 185H, -CHCH_2_CH_2_CH_2_CH_2_-). The GPC and FTIR spectra of PPD were displayed in Figs. S2 and S3, respectively.

### Synthesis of PPQ

Briefly, *p*-aldehyde benzoic acid (78 mg) was dissolved in a vacuum-dried round-bottom flask with anhydrous DMF (2 ml), and 2 ml of anhydrous DMF dissolved in NHS (71.35 mg) and *N*,*N*′-dicyclohexylcarbodiimide (DCC; 127.92 mg) was added to the above reaction vessel and stirred for another 2 h. After that, PEG-PLL (100 mg) was added dropwise upon stirring for 24 h. Next, the above mixture was precipitated with ether, centrifuged, and vacuum dried for 24 h to obtain PPQ powder. ^1^H NMR (500 MHz, CDCl_3_) of Fig. S1C: 10.24 (s, 24H, -CHO-), 3.64 (s, 468H, -OCH_2_CH_2_-), 3.34 (s, 3H, -OCH_3_), 2.96 (s, 48H, NH_2_CH_2_CH-), 2.70 (s, 12H, -NHCH_2_CH-), 1.59 (d, 186H, -CHCH_2_CH_2_CH_2_CH_2_-), 8.03 (s, 51H, -ArH-), 8.30 (s, 45H, -ArH-).10.24 (s, 24H, -ArCHO). The GPC and FTIR spectra of PPQ were displayed in Figs. S2 and S3, respectively.

### Synthesis of PPQV

Typically, PPQ (45 mg) and V9302 (18 mg) were dissolved in a nitrogen atmosphere of DMF (5 ml), and the mixture was stirred vigorously for 12 h. Next, the reaction liquid was pretreated with anhydrous DMF and centrifuged at 4 °C (8,000 rpm, 10 min). The resultant PPQV powder was obtained via vacuum drying. ^1^H NMR (500 MHz, CDCl_3_) of Fig. S1D. The GPC and FTIR spectra of PPQV were displayed in Figs. S2 and S3, respectively.

### Synthesis of PPQV@C

The conjugation CA4P on the surface of PPQV was achieved through electrostatic adsorption [54]. Briefly, PPQV and CA4P were dissolved in PBS under stirring overnight. The mixture was transfer to a dialysis bag (MWCO, 3,500 Da) and dialyzed with double-distilled water for 48 h, which was then freeze-dried, and the product was harvested, which was denoted as PPQV@C.

### Preparation of PPD/PPQV@C nanosystem

The core polymer PPQV@C (20 mg) and shell polymer PPD (40 mg) were dissolved in PBS (4 ml) at a 1:2 ratio under stirring overnight. Next, the mixture was dialyzed using the above method. The final micelles system was collected via lyophilization and named as PPD/PPQV@C.

### Materials characterization

Briefly, NMR hydrogen spectroscopy, UV spectrophotometry (U-3900, HITACHI, Japan), and gel permeation chromatography (Agilent 1260 Infinity II, USA) were used to verify the functionalized modifications in the preparation of PPD/PPQV@C micelles. The morphology and particle size of the core prodrug micelle PPQV and final PPD/PPQV@C micelle were characterized by TEM (Talos F200X, FEl, USA). The elemental content of the compound is measured by x-ray photoelectron spectroscopy (Thermo Scientific K-Alpha). The molecular structure of the compound was observed by H Nuclear Magnetic Resonance Spectra (ADVANCE NEO 500, GER). Structural changes in compounds are monitored by Fourier Transform Infrared Spectrometer (Nicolet iS50, USA). The hydration diameter and zeta potential of PPQV and PPD/PPQV@C were measured by DLS (90 Plus PALS, Brookhaven, USA) equipped with potential analyzer.

### Biostability testing of micelles

PPD/PPQV@C was respectively dissolved in pH 7.4 and 6.8 containing 10% FBS and incubated at 37 °C for 7 d. Samples are taken daily, the particle size of the nanosystem is measured using DLS, and the corresponding size change is mapped.

### Drug release behaviors

Briefly, PPD/PPQV@C (3 mg) polymer micelles were dissolved into 600 μl of PBS at different pH, and the mixture was transferred to a dialysis tubing (MWCO, 3,500 Da) after full dissolution. Then, the dialysis tubing was submerged in 6 ml of PBS with the corresponding pH described above. At specific intervals (0.5, 1, 2, 3, 5, 10, 12, 24, and 48 h), 300 μl of solution outside the dialysis tubing was taken for testing, and the corresponding amount of solution was supplemented. The absorbance of CA4P and V9302 was respectively measured using NanoDrop at 290 and 274 nm, and the drug concentration and release curve were calculated or plotted according to its standard curve.

### Cell culture

Human MDA-MB-231 and HUVECs were cultured in Dulbecco’s modified Eagle’s medium (DMEM) containing 10% FBS (37 °C, 95% air/5% carbon dioxide) plus 1% penicillin–streptomycin.

### Cell viability and cytotoxicity assays

MDA-MB-231 cells seeded in 48-well plates were cocultured with PBS, V9302 (4.8 μM), PPQV, PPD/PPQV, and PPD/PPQV@C (20 μg/ml, the same dosage of V9302) with pH 7.4 or pH 6.8 for 24 h. HUVECs were incubated with PBS, CA4P (3 μM), and PPD/PPQV@C (20 μg/ml, the same dosage of CA4P) for 24 h. At the end of the incubation, the above cell samples were treated with CCK-8 kit (Sevenbio) and cell viability was calculated according to the instructions. The untreated group of normal cells was used as the control group.

### Apoptosis assay

MDA-MB-231 cells seeded in 6-well plates were treated with the above administrations for 24 h. Subsequently, the cell samples were collected and incubated with Annexin V-FITC/PI kit according to the instructions. Finally, the apoptosis degree was detected by the flow cytometer (FCM, BD FACSCelesta).

### Mitochondrial membrane potential assay

The typical JC-1 probe was used to measure the mitochondrial membrane potential damage. Generally, MDA-MB-231 cells were seeded in confocal dishes cocultured with above treatments for 12 h. The medium was then discarded, and the cells were washed with PBS. Next, JC-1 staining solution (Beyotime Biotechnology) was added to each dish and incubated at 37 °C for 20 min. Finally, the damage of mitochondria was observed using a confocal laser scanning microscope (OLYMPUS FV3000).

### Cellular uptake evaluation by CLSM and FCM

MDA-MB-231 cells seeded in confocal dishes were treated with V9302 and PPD/PPQV@C (pH 7.4 and 6.8) both labeled with FITC for 4 and 12 h, respectively. Next, the above cell samples were cultured with 4% paraformaldehyde, 0.2% Triton, ActinRed 555 (Thermo Fisher), and DAPI (4′,6-diamidino-2-phenylindole). Finally, the level of cell uptake of micelles was observed and photographed with CLSM. At the same time, MDA-MB-231 cells seeded in 6-well plates were administrated with the above treatment groups for 4 and 12 h. Subsequently, the cell samples were harvested and measured by FCM.

### MDA-MB-231 cell-based MCS construction and infiltration assay

Firstly, MDA-MB-231 cell-based MCSs was constructed according to our previous literature [39]. Next, V9302 and PPD/PPQV@C (pH 7.4 and 6.8) both labeled with FITC were cocultured with the above MCSs for 4 and 12 h. After treatment, MCSs were transferred to a clean dish and observed using CLSM.

### Lysosomal escape evaluation

MDA-MB-231 cells seeded in confocal dishes were treated with PPD/PPQV@C (pH 7.4 and 6.8) labeled with FITC for 1, 4, and 12 h, respectively. Next, the above cells were cultured with Lysotracker Red at 37 °C for 0.5 h, followed by PBS washing, and finally imaged by CLSM.

### Intracellular ROS detection

MDA-MB-231 cells placed in confocal dishes were pretreated with PBS, V9302, PPQV, PPD/PPQV, and PPD/PPQV@C (pH 7.4 and 6.8) for 12 h. Subsequently, serum-free medium containing 10 μM DCFH-DA (1 ml) was added to the above mixture and incubated at 37 °C for another 20 min. Finally, the fluorescence signal of the DCF was observed under a confocal microscope with an excitation wavelength of 488 nm and an emission wavelength of 525 nm.

### Detection of reducing GSH

MDA-MB-231 cells seeded in 6-well plates were firstly incubated with PBS, V9302, PPQV, PPD/PPQV, and PPD/PPQV@C (pH 7.4 and 6.8) for 12 h. The cell pellet was obtained by trypsinization, and the intracellular GSH content was determined using the reductive GSH assay kit (Beijing Boxbio Science & Technology Co. Ltd.) after cell counting, according to the instruction. Confocal images of intracellular detection of GSH were processed according to ThiolTrace Violet 500 kit instructions (AAT Bioquest) and photographed using CLSM.

### Intracellular ATP detection

After the above administrations, MDA-MB-231 cells were lysed on ice for 0.5 h and the supernatant was obtained by centrifugation (12,000*g*, 10 min). Then, the ATP content in the cells was determined by the ATP detection kit (Beyotime Biotechnology).

### Intracellular glutamine measurement

MDA-MB-231 cells seeded in 6-well plates were incubated with PBS, V9302, PPQV, PPD/PPQV, and PPD/PPQV@C (pH 7.4 and 6.8) for 24 h. Cell pellets were obtained by trypsinization, and intracellular glutamine content was determined by the Glutamine Content Assay Kit (Geruisi-bio) after cell counting.

### Metabolomic analysis

Typically, MDA-MB-231 cells seeded in 150-mm dishes were treated with PBS and PPD/PPQV@C for 24 h, respectively. The cell samples were rinsed and collected on ice, followed by freezing with liquid nitrogen. Finally, the sample was used for the metabolomics study with the assistance of the Bioprofile Technology Company Ltd. (Shanghai, China).

### Western blot assay

Firstly, MDA-MB-231 cells were treated with above administrations for 24 h. Secondly, the cells were lysed and centrifuged, the cellular proteins were extracted, and protein quantification was performed using the BCA (bicinchoninic acid) protein assay kit. Next, equal amounts of protein were analyzed by electrophoresis on a 10% sodium dodecyl sulfate polyacrylamide gel after boiling for 10 min and mixing with 5× loading buffer. The expression levels of caspase-3, Bcl-2, Bax, and ASCT2 were visualized by chemiluminescence imaging system (VILBER FUSION Solo6S EDGE) and quantitatively analyzed by ImageJ. The expression of actin was chosen as an internal reference.

### Cell scratch assay

Generally, logarithmic grown HUVECs were placed in a 6-well plate and cultured overnight until the cells were 100% confluent. A vertical scratch was generated using a tip of a p200 pipette plate covered by the above cells, and the initial scratch area was imaged by a microscope (Olympus). Subsequently, the above cells were cultured with PBS, CA4P, and PPD/PPQV@C (pH 7.4 and 6.8) for 24 h, and the scratch area was further imaged by a microscope (Olympus).

### Cellular vascularization

Typically, the Matrigel (Corning) was added to the 24-well plate with a density of 30 μl per well and left overnight in a 4 °C freezer. After that, the plate was transferred to a 37 °C incubator and stayed for 0.5 h. The HUVECs were plated on the above 24-well plate containing Matrigel with a density of 10^5^ cells per well. Next, PBS, CA4P, and PPD/PPQV@C (pH 7.4 and 6.8) were added and cocultured for 8 h. Finally, angiogenesis was photographed using a microscope (Olympus).

### Cell invasion assay

Firstly, the Matrigel was diluted to 1 mg/ml with serum-free medium and added to the upper chamber of transwell, which was then left overnight in the 4 °C freezer. Next, the plate was transferred to the 37 °C incubator and incubated for 0.5 h, and serum-free medium (100 μl) was then added to each well and hydrated for 2 h. After that, the HUVECs were seeded in the upper chamber at 10^5^ cells per well and the complete medium (500 μl) was added in the bottom chamber. After treatment with PBS, CA4P, and PPD/PPQV@C (pH 7.4 and 6.8) for 24 h, the noninvaded cells were removed with cotton swab scrubbing, and the invaded cells were cultured with paraformaldehyde and stained with crystal violet. The degree of invasion was imaged by an inverted fluorescence microscope.

### Cell cycle arrest assay

Typically, HUVECs seeded on 6-well plates were treated with the administration described above for 24 h. Subsequently, cells were washed and stained according to the protocols of the Cell Cycle Assay Kit (Elabscience Biotechnology Co. Ltd.) and detected by FCM measurement.

### Establishment of tumor-bearing models and in vivo anticancer therapy

Briefly, 100 μl of MDA-MB-231 cells (1 × 10^6^) was injected subcutaneously into the groin of BALB/c nude mice (female mice, 5 to 6 weeks old). When the tumor size reached about 70 to 80 mm^3^, mice were randomly divided into 5 groups (*n* = 6) and injected intravenously with saline, V9302, PPQV, PPD/PPQV, and PPD/PPQV@C (equivalent of 70 mg/kg of V9302). The above treatments were given every other day for a total of 18 d. Meanwhile, body weight and tumor volume are measured every 2 d. Tumor volume is calculated by the following formula: tumor volume = *ab*^2^/2 (*a* and *b* are the maximum and minimum diameter of the tumor, respectively). After the end of 18-d dosing, the survival of mice was continually observed for another 33 d.

### Biodistribution of micelle nanosystem

MDA-MB-231-bearing BALB/c nude mice were injected with FITC-labeled V9302 (70 mg/kg) or PPD/PPQV@C. The mice were euthanized after 6 h of injection, the tumor and major organs were harvested, and the fluorescence distribution of the major organs was imaged by In vivo Imaging System (PerkinElmer IVIS Lumina LT Series III).

### Evaluation of blood safety

Briefly, mice were given intravenous injection of PPD/PPQV@C and saline, blood samples were then excreted out, and the plasma was separated by centrifugation (900*g*, 10 min). Next, hematological parameters and liver/kidney function indicators were measured for the evaluation of blood safety, according to the manufacturer’s instructions.

### H&E, TUNEL, and immunofluorescence assays

After the end treatment of 18 d, the main major organs (heart, liver, spleen, lung, and kidney) and tumor tissue of the tumor-bearing mice were collected, sliced, and examined by H&E, TUNEL, Ki67, and CD31 assays separately. Firstly, the above sections were stained with hematoxylin and eosin and the morphological analysis was visualized by a light microscope (OLYMPUS IX73). Secondly, tumor sections were stained with an orthotopic cell death assay kit (Beyotime) and imaged by CLSM for the TUNEL assay. Finally, tumor sections were incubated with Ki67/CD31 primary antibodies and secondary antibodies labeled with FITC, and imaged by CLSM for immunofluorescence analysis.

### Anti-angiogenesis evaluation in vivo

Typically, 100 μl (1 × 10^6^) of MDA-MB-231 cells was injected subcutaneously into the back of mice. When tumor size reached approximately 70 to 80 mm^3^, mice were randomly divided into 6 groups and injected intravenously with saline, V9302, CA4P, PPQV, PPD/PPQV, and PPD/PPQV@C, with V9302 at a dose of 70 mg/kg (*n* = 6) and a frequency of one time per day. After 7 d of treatment, the density and intensity of blood vessels around the tumor in mice were observed using Photoacoustic Multimode Small Animal In Vivo Imaging System (Guangzhou G-Cell Technology Co. Ltd.).

### Statistical analysis

All statistical analysis was performed using the Origin 2022. The values were expressed as means ± SD for data that were normally distributed. The confidence levels of 95% and 99% were regarded as a significant difference. For multi-group comparison, *P* values were derived from one-way analysis of variance (ANOVA). For all comparisons, *P* < 0.05 test was considered statistically significant (*P < 0.05, **P < 0.01, ***P < 0.001).

## Data Availability

The data used to support the findings of this work are available from the corresponding authors upon request.
