# Supplementary material for: Delivery-Graded Programmable Micelles Achieve Enhanced Tumor Starvation through Combined Glutamine Deprivation and Angiogenesis Inhibition
Source: Research (Wash D C). 2025 Sep 5;5:0858. doi: 10.34133/research.0858 (PMC12411305; doi:10.34133/research.0858)
Supplement: Supplementary 1 — Figs. S1 to S29 [file research.0858.f1.docx]

**Supplemental Materials**

Delivery-Graded Programmable Micelles Achieve Enhanced Tumor Starvation through Combined Glutamine Deprivation and Angiogenesis Inhibition

*Xuan Wei, Jiamin Cheng, Meijuan Geng, Siyu Chen, Liyang Gong, Siyu Meng, Keying Chen, Ziyan Wang, Zhang Yuan, Kaiyong Cai and Liangliang Dai****

X. Wei, J. Cheng, M. Geng, S. Chen, L. Gong, S. Meng, K. Chen, Z. Wang, Z. Yuan and L. Dai

Institute of Medical Research, Northwestern Polytechnical University, Xi’an 710072, P. R. China, E-mail: [liangliangdai@nwpu.edu.cn](mailto:liangliangdai@nwpu.edu.cn)

K. Cai

Key Laboratory of Biorheological Science and Technology, Ministry of Education, College of Bioengineering, Chongqing University, Chongqing 400044, P. R. China

***List of Contents***

[**Figure S1**](#_heading=h.rl60kanl87ld)[^1^H NMR of PEG-PLL, PPQ, PPQV and PPD polymers. 5](#_heading=h.rl60kanl87ld)

[**Figure S2** The GPC traces of of PEG-PLL, PPQ, PPQV and PPD polymers. 6](#_heading=h.g2abvt5gx70)

[**Figure S3** FTIR spectra of various polymers..................................................................7](#_heading=h.dl1fozita678)

[**Figure S4** XPS plot of PPD/PPQV@C micelle. 8](#_heading=h.lmwe42e531uo)

[**Figure S5** UV absorption standard curve for V9302 and CA4P. 9](#_heading=h.50bltldgjouz)

[**Figure S6** CMC of PPD/PPQV@C micelle. 10](#_heading=h.wg76xiv3mamk)

[**Figure S7** The cumulative amount of V9302 and CA4P released from nanosystem…..11](#_heading=h.5mrc1zgus821)

[**Figur**e **S8** Particle sizes of PPD/PPQV@C nanosysytem in 10% BSA. 12](#_heading=h.7tdkb3nct25o)

[**Figure S9** Quantitative fluorescence intensity of the MDA-MB-231 uptake. 13](#_heading=h.v6giihtujgu4)

[**Figure S10** The content of intracellular V9302 in HPLC. 14](#_heading=h.k0361f7ljxpw)

[**Figure S11** Cytotoxicity of V9302 to MDA-MB-231 cells. 15](#_heading=h.jm9ndfokiqfp)

[**Figure S12** Quantitative analysis of apoptosis in MDA-MB-231 cells. 16](#_heading=h.tzxgljn776xm)

[**Figure S13** Related fluorescence quantitative statistics for JC-1. 17](#_heading=h.vdbfn42qk1jn)

[**Figure S14** Quantitative statistics of proteins expressions in MDA-MB-231. 18](#_heading=h.tog5f6d9o0bn)

[**Figure S15** Intracellular glutathione content at different administration times. 19](#_heading=h.dgw0ulw0hvf2)

[**Figure S16** Quantitative statistics of ROS in MDA-MB-231. 20](#_heading=h.ll7q9ysne3w)

[**Figure S17** Quantitative fluorescence intensity of the HUVECs uptake. 21](#_heading=h.e6ykgzqgrygg)

[**Figure S18** Quantitative cell cycle analysis of HUVECs cells 22](#_heading=h.x4wwh324zunb)

[**Figure S19** Quantitative analysis of angiogenesis of HUVECs. 23](#_heading=h.gjf8zfay12cn)

[**Figure S20** Correlated quantification analysis of wound healing ratio. 24](#_heading=h.d1xjckec138)

[**Figure S21** Quantitative statistics of invasion rate. 25](#_heading=h.1a313r55t3zu)

[**Figure S22** Heat map of nude mice blood routine. 26](#_heading=h.uzwvkposhkvk)

[**Figure S23** Weight change curves. 27](#_heading=h.szf68s2xbnea)

[**Figure S24** H&E staining analysis of major tissues. 28](#_heading=h.mfx0ykmejunt)

[**Figure S25** Quantitative fluorescence analysis of biodistribution. 29](#_heading=h.h12wlfk91h9i)

[**Figure S26** Tumor volume change curves of the mice. 30](#_heading=h.ru0ov781y3rh)

[**Figure S27** Quantitative statistics of Tunel, Ki67 and CD31. 31](#_heading=h.jz4n6jjq30lg)

[**Figure S28** Images of blood vessels in mice after free CA4P administration. 32](#_heading=h.ww77c7z6r6re)

[**Figure S29** Quantitative statistics of tumor vascular density. 33](#_heading=h.j5dt9qas2syv)

**A**

**
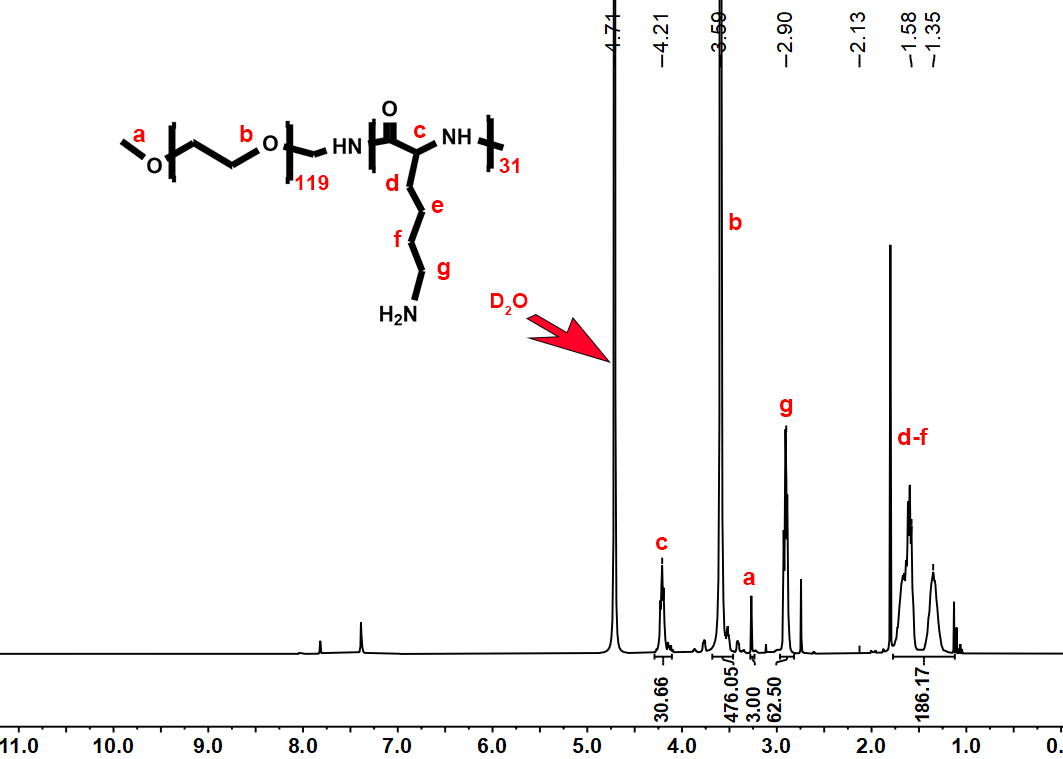
**

**B**


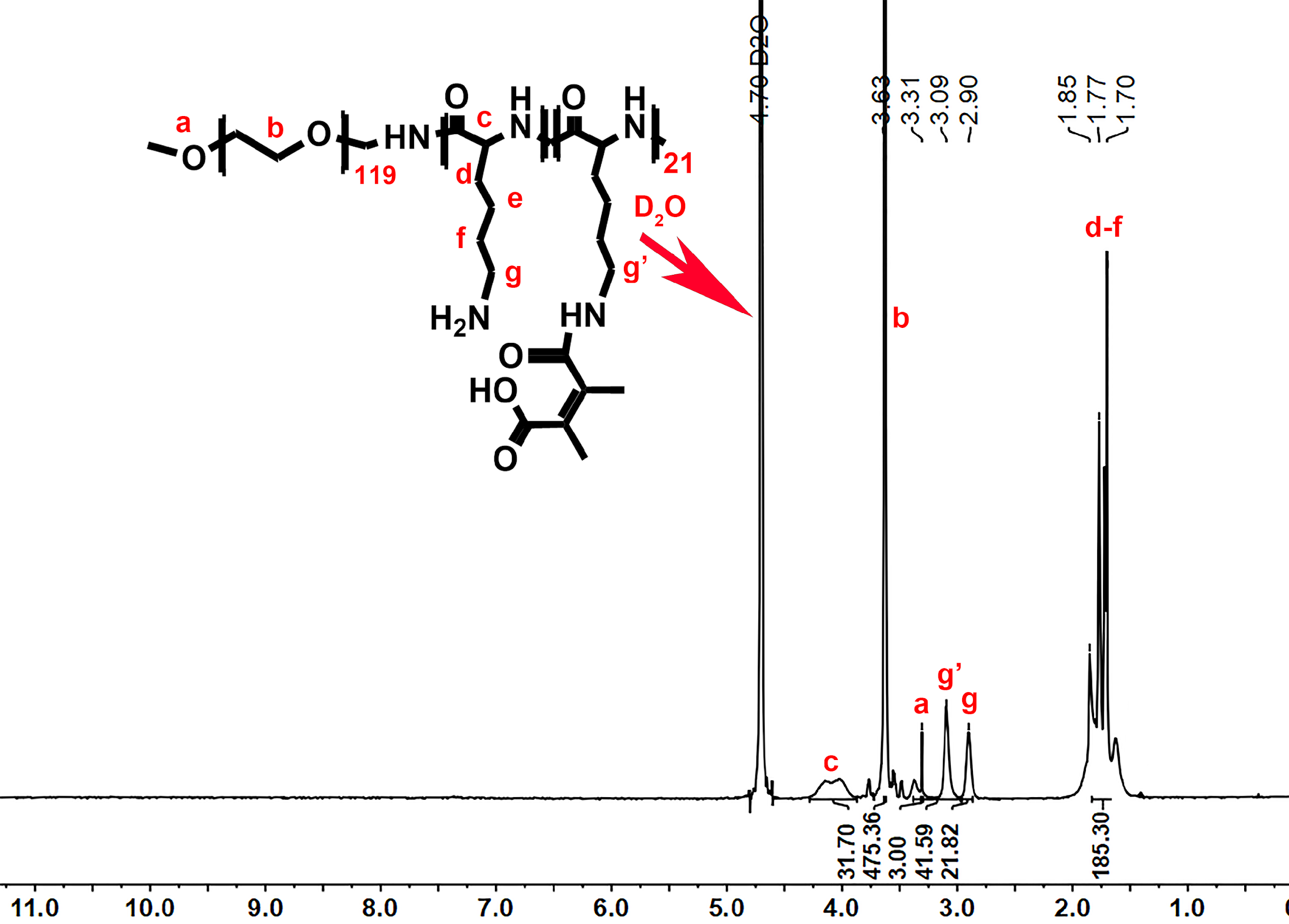


**C**

**
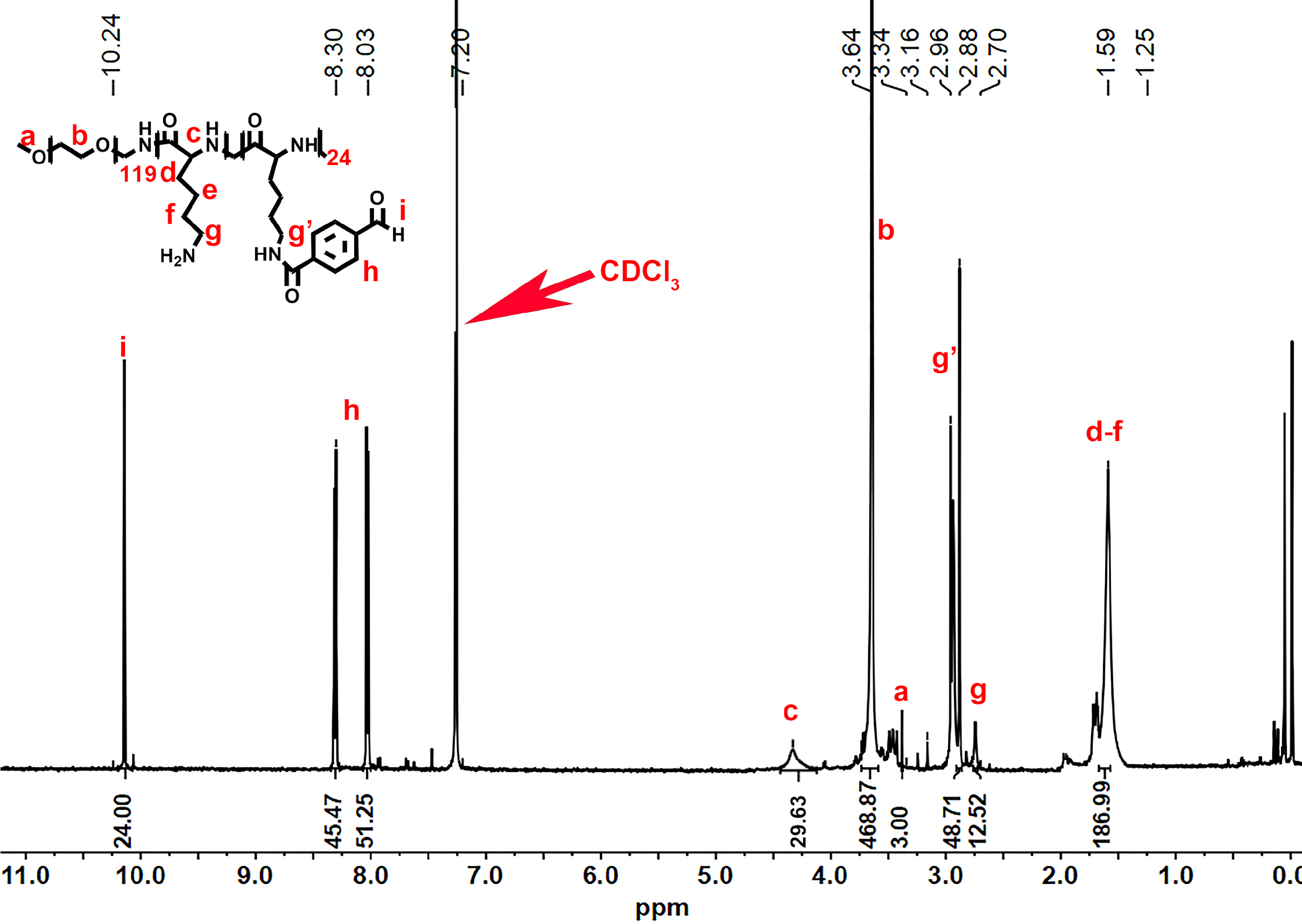
**

**D**


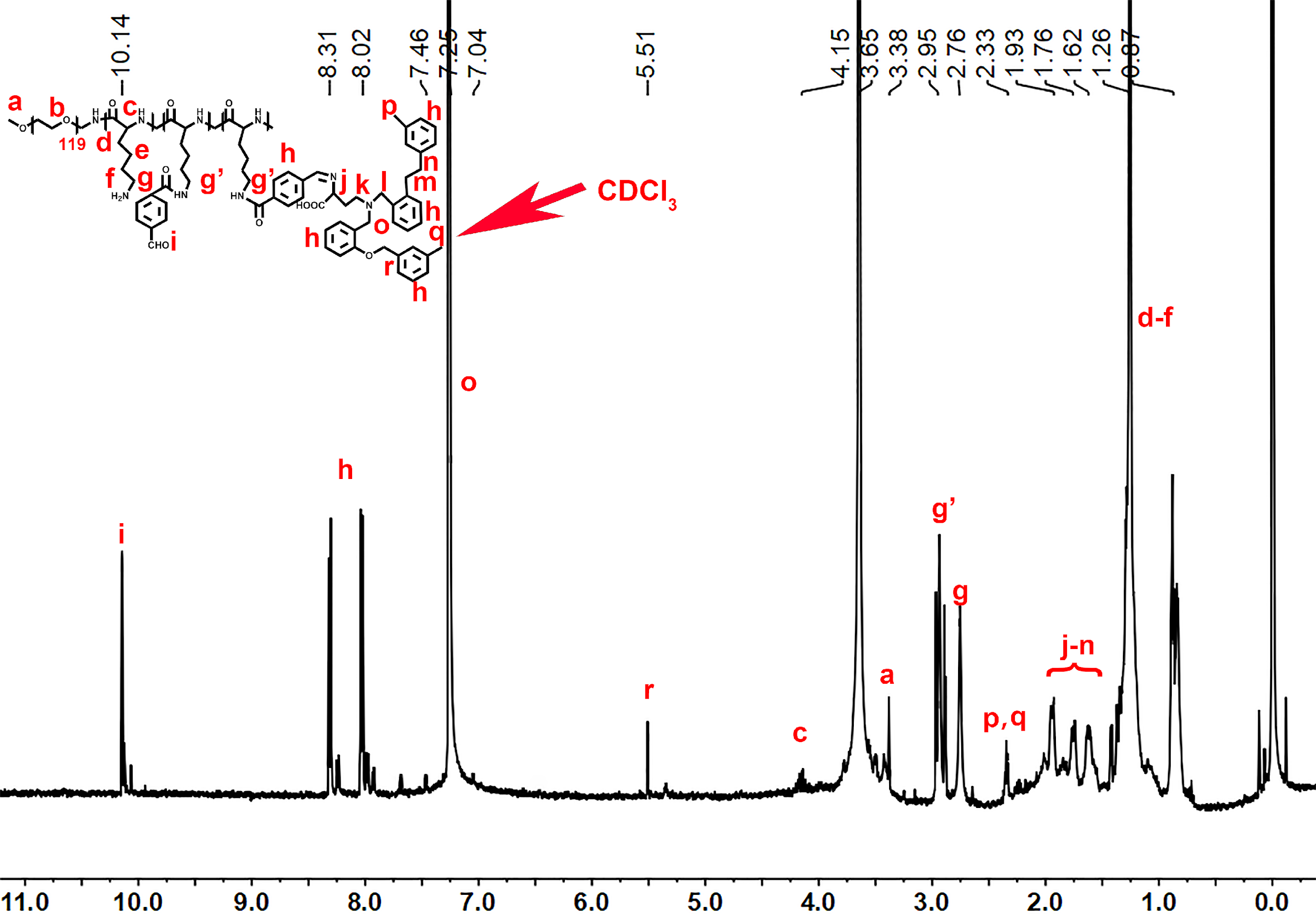


**Figure S1** ^1^H NMR spectra (500 MHz, 298 K) of (a) PEG-PLL (D_2_O as a solvent), (b) PPD (D_2_O as a solvent), (c) PPQ (CDCl_3_ as a solvent), (d) PPQV (CDCl_3_ as a solvent).


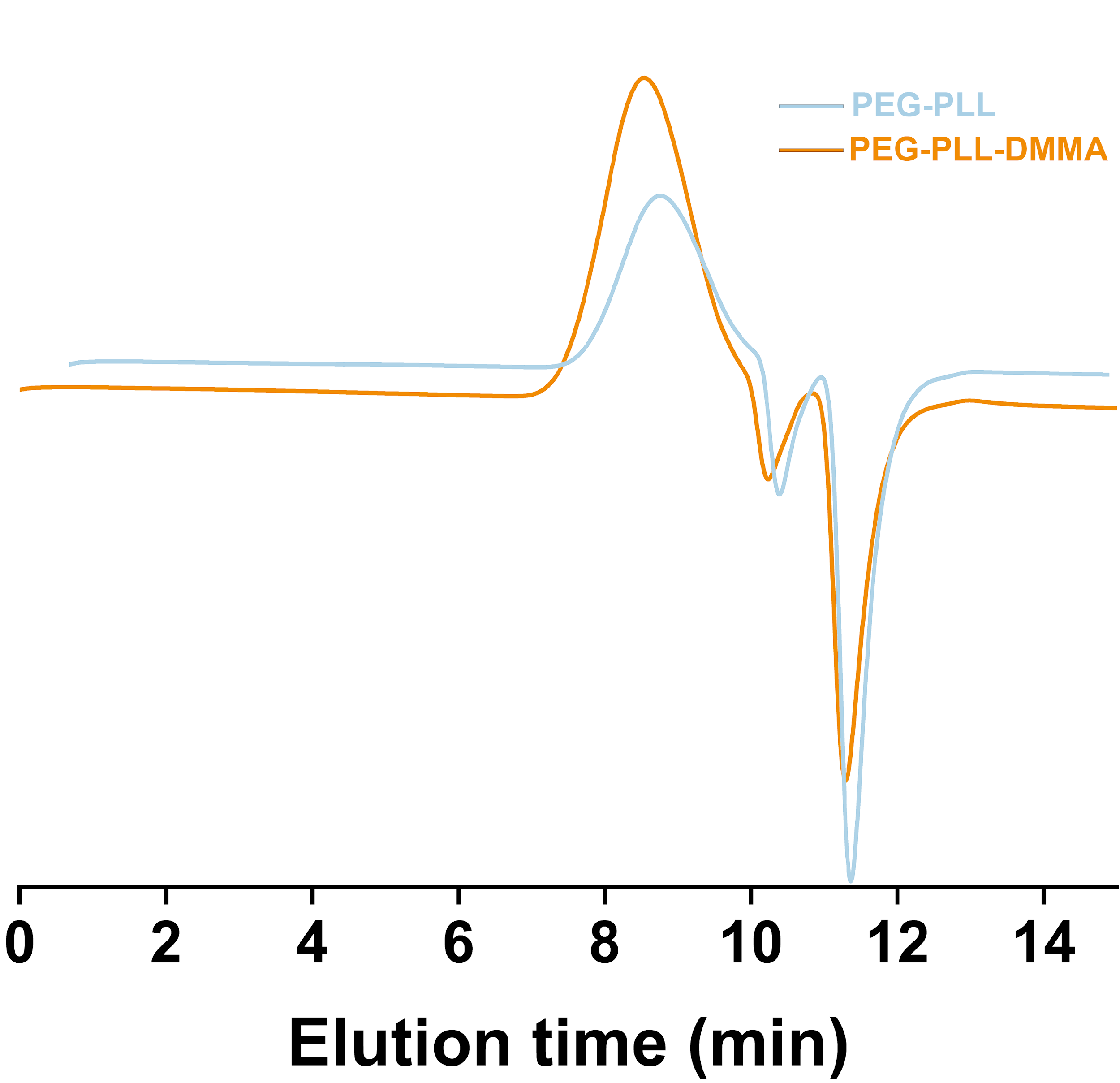


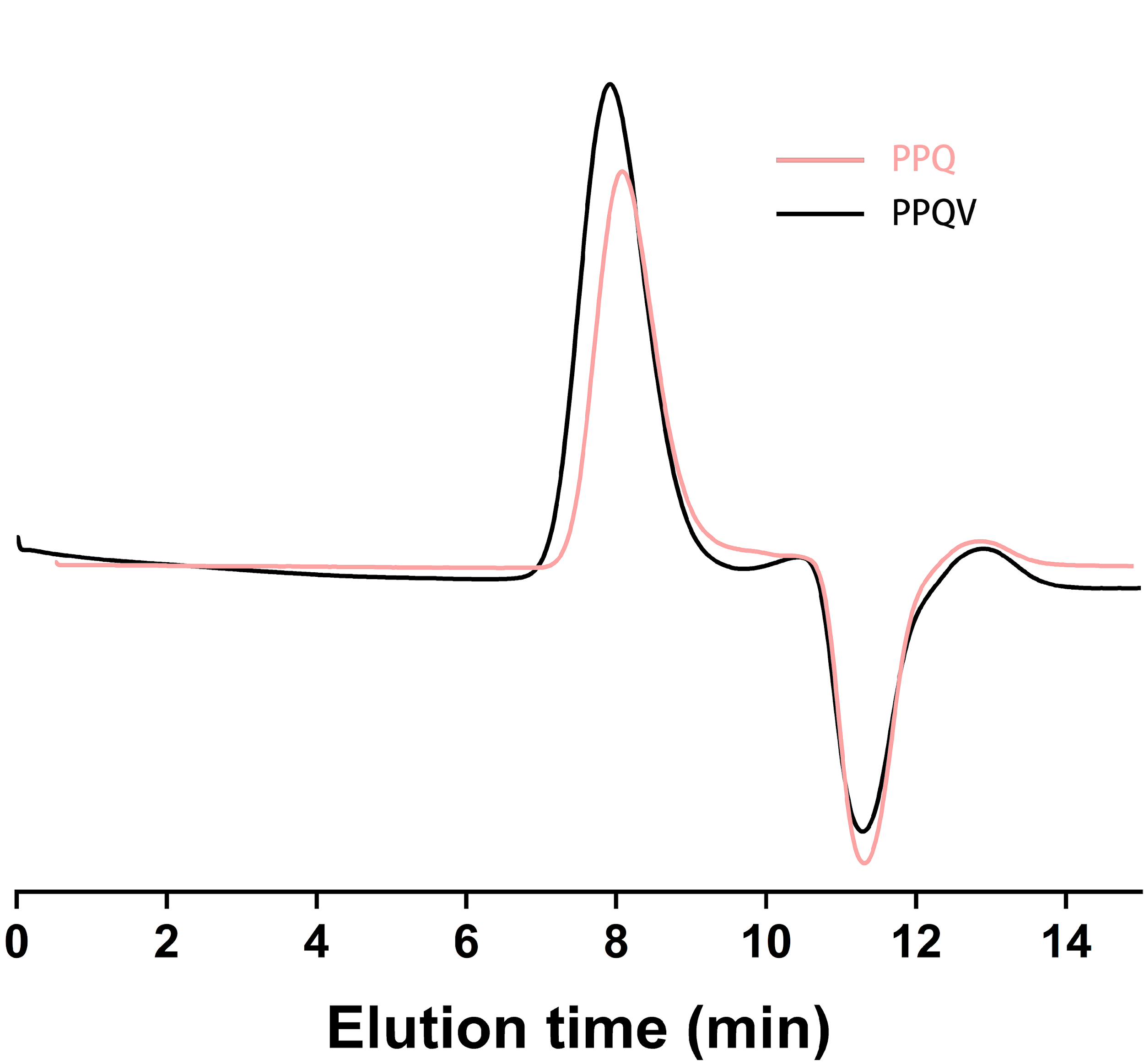


**Figure S2** The GPC traces of PEG-PLL, PEG-PLL-DMMA polymers (D_2_O as a solvent, Agilent 1260 InfinityII), PPQ and PPQV polymers (CDCl_3_ as a solvent, Waters 1525).


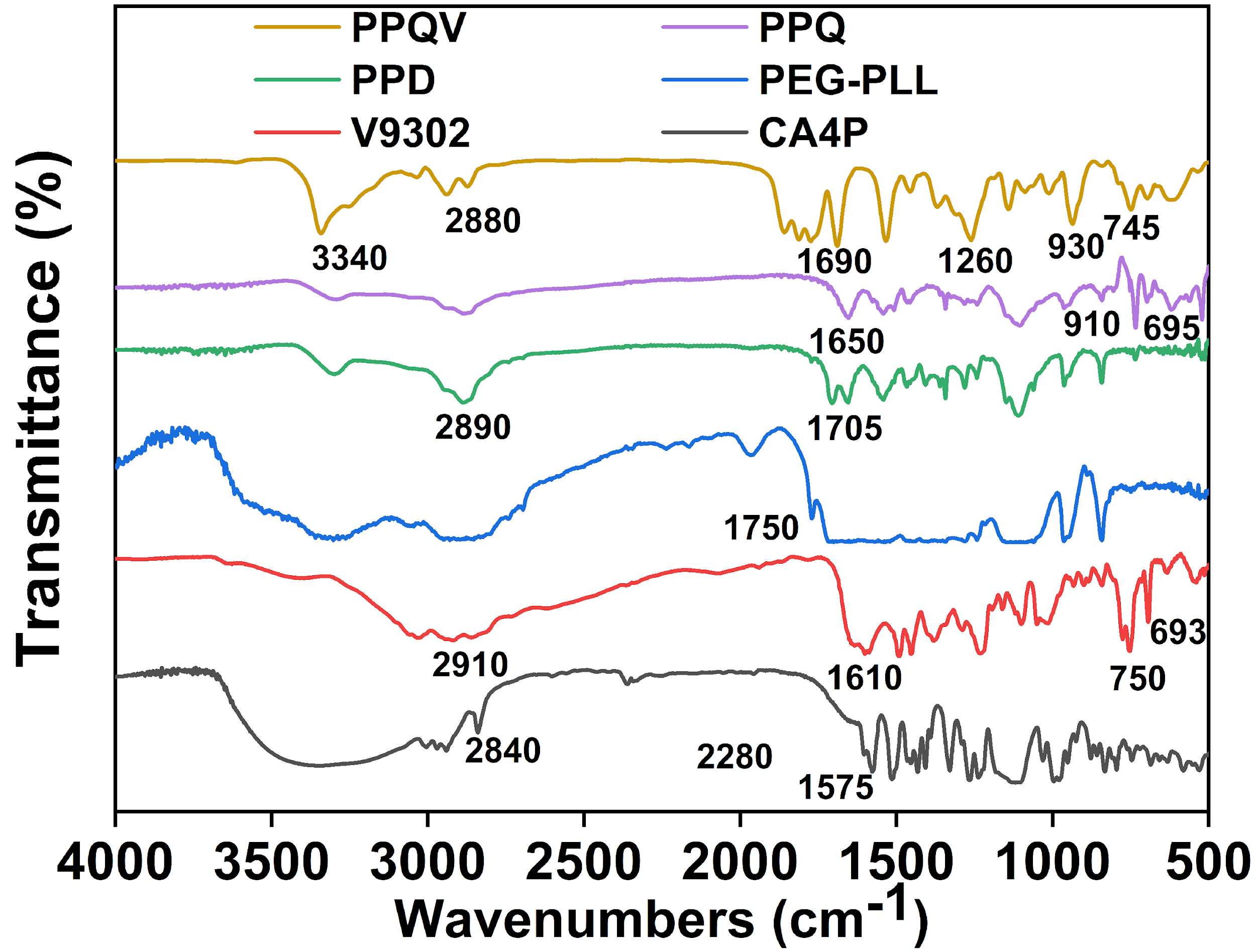


**Figure S3** FTIR spectra of V9302, CA4P, PEG-PLL, PPD, PPQ and PPQV polymers, respectively.

As shown in Fig. S3, FTIR spectroscopy confirmed the successful stepwise syntheses: PEG-PLL exhibited a characteristic amide absorption at 1750 cm⁻¹; subsequent introduction of DMMA to PEG-PLL generated new carboxyl peaks at 2890 cm⁻¹ (O-H stretching) and 1705 cm⁻¹ (C=O stretching), verifying DMMA modification [37]; grafting of 4-formylbenzoic acid onto PEG-PLL produced distinct aromatic C-H out-of-plane bending vibrations at 910 cm⁻¹ and 695 cm⁻¹, characteristic of benzene rings, these signals were attributed to the newly incorporated 4-formylbenzoic acid moiety; PPQV spectrum displayed additional characteristic absorption bands: a C=O stretching vibration at 1690 cm⁻¹, C-H stretching vibrations at 2880 cm⁻¹, and a C-O stretching vibration at 1250 cm⁻¹, these new peaks are consistent with the expected functional groups introduced by V9302 [23], thereby confirming the successful synthesis of PPQV.


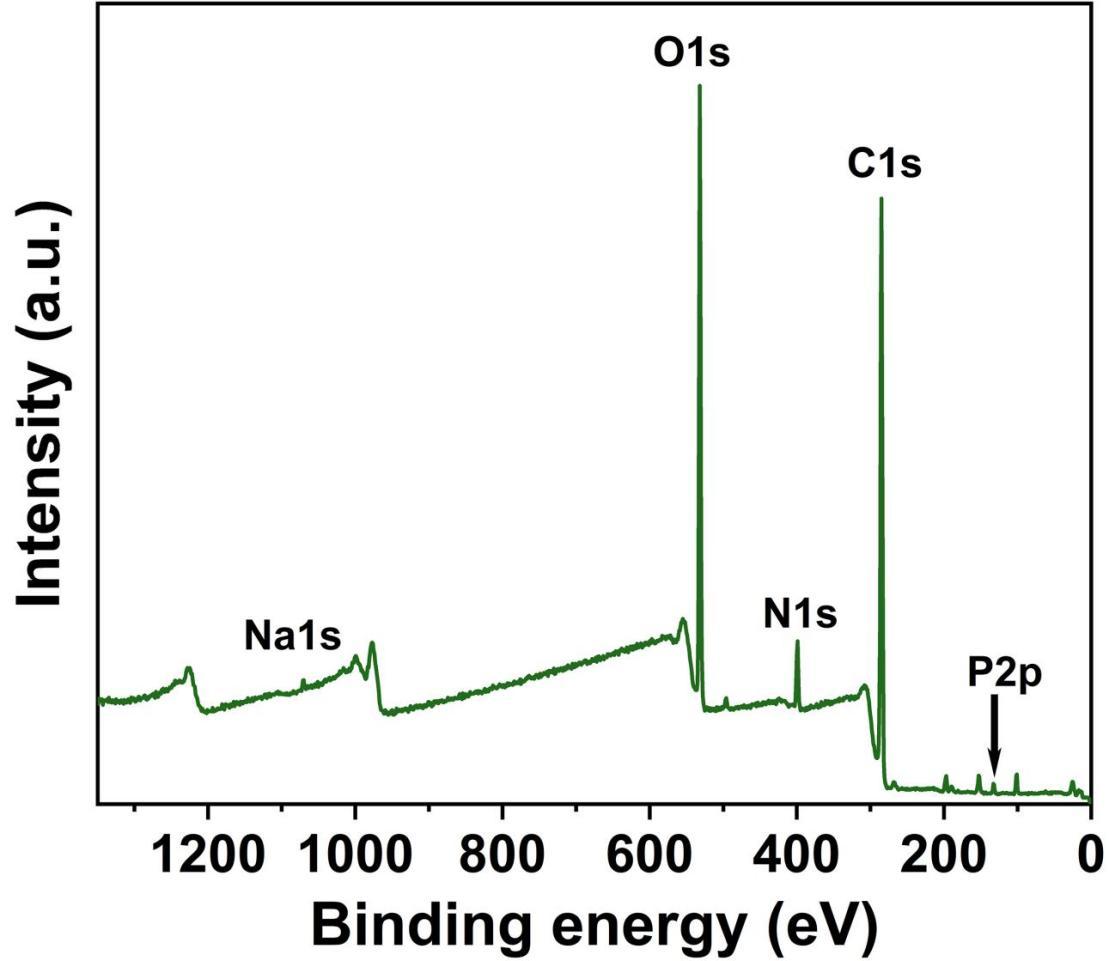


**Figure S4** XPS plot of PPD/PPQV@C micelle.

**A**


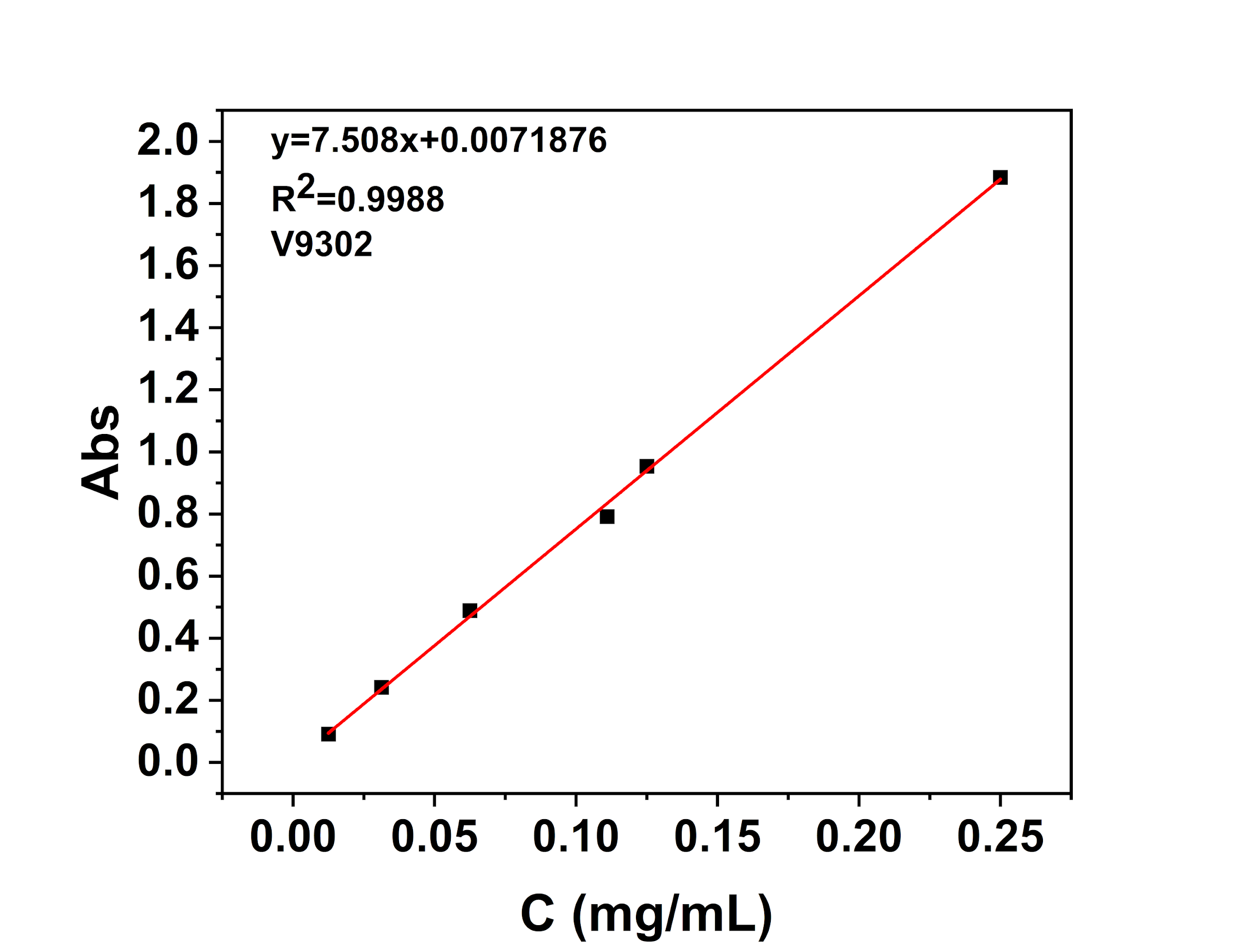


**B**

**
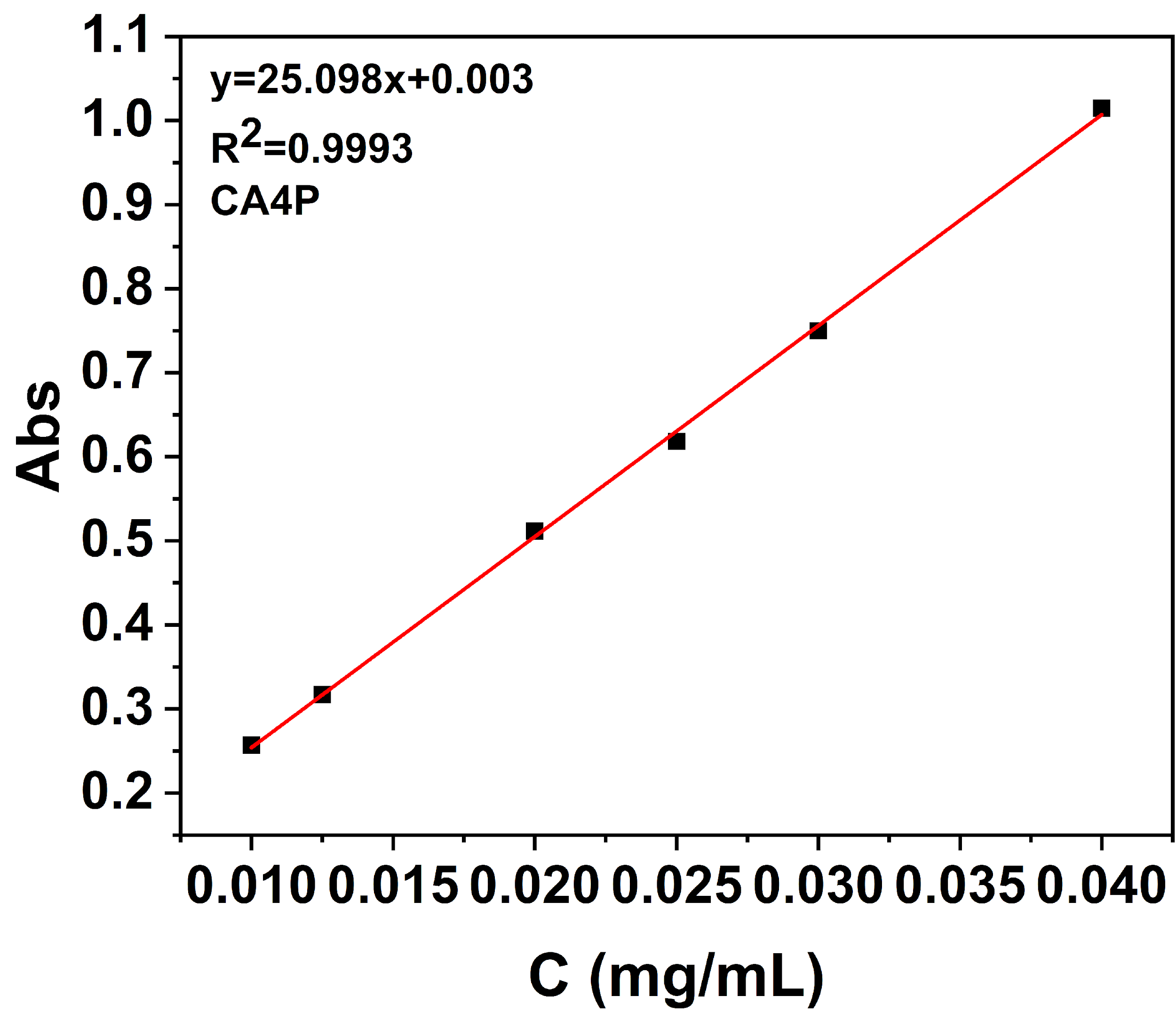
**

**Figure S5** UV absorption standard curves for V9302 and CA4P at 274 nm and 290 nm, respectively.


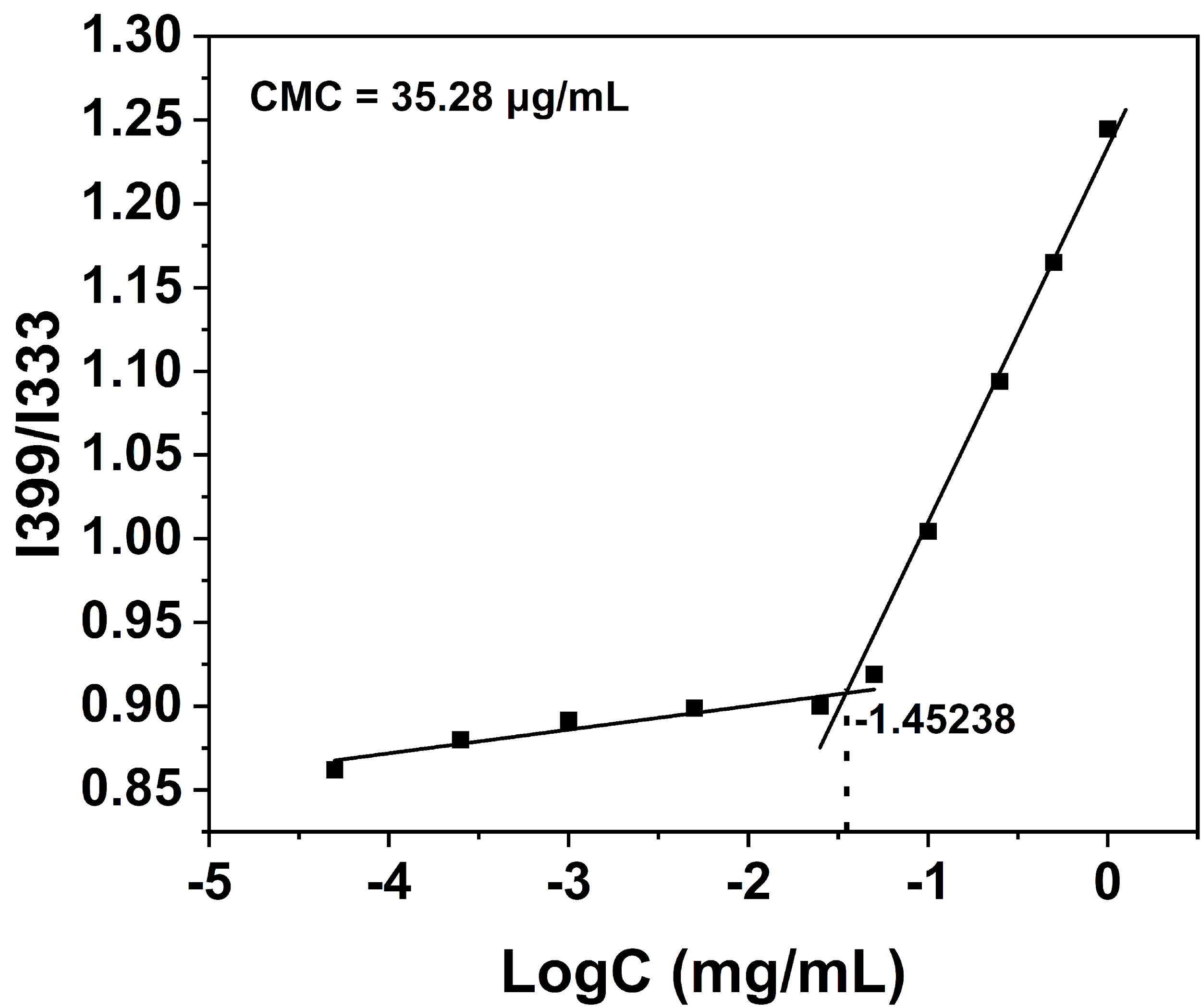


**Figure S6** Critical micelle concentration (CMC) of PPD/PPQV@C micelle.


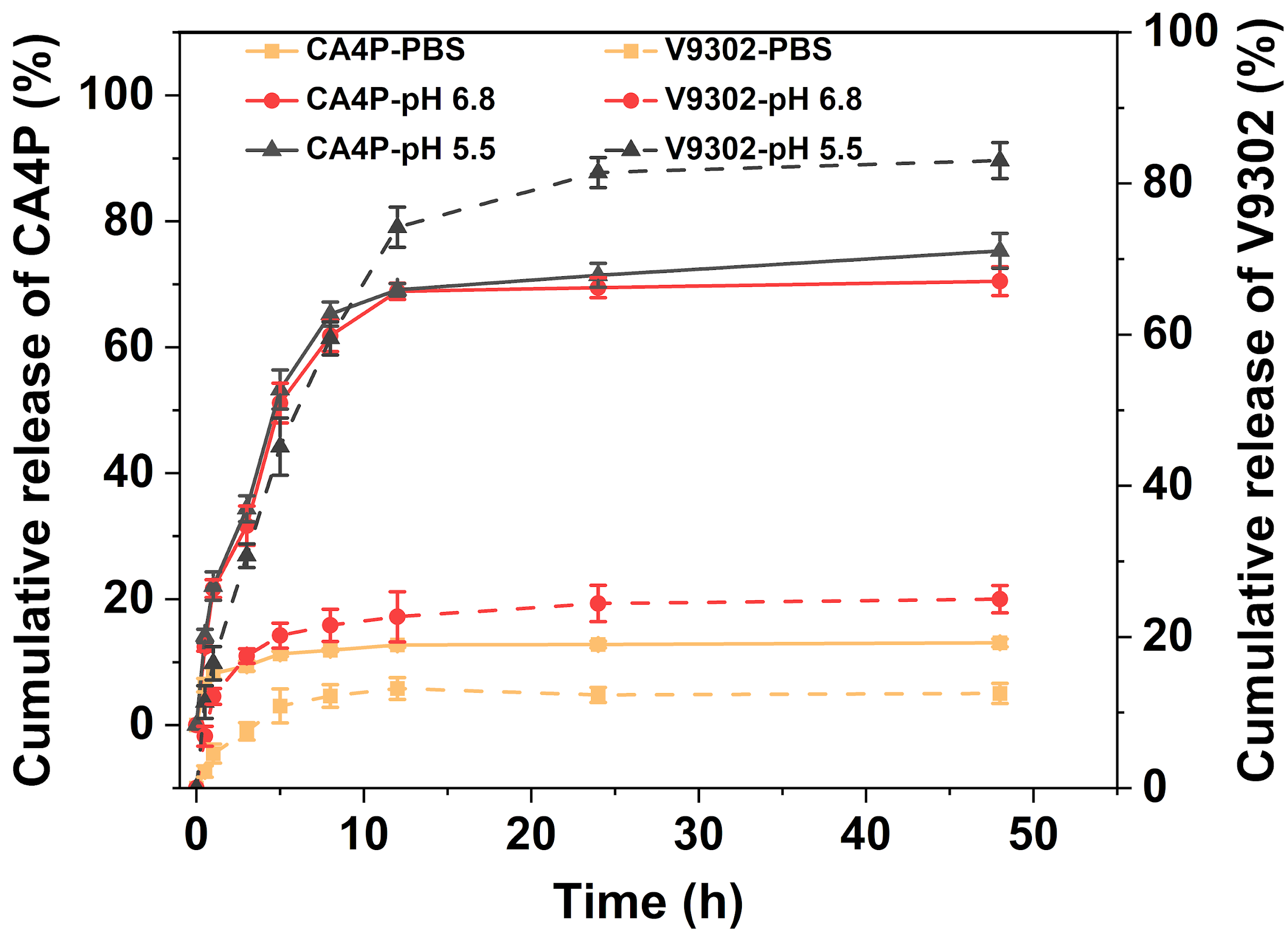


**Figure S7** The cumulative amount of V9302 and CA4P released from PPD/PPQV@C under the same conditions. Error bars represent mean ± standard deviation (n = 3 independent samples).


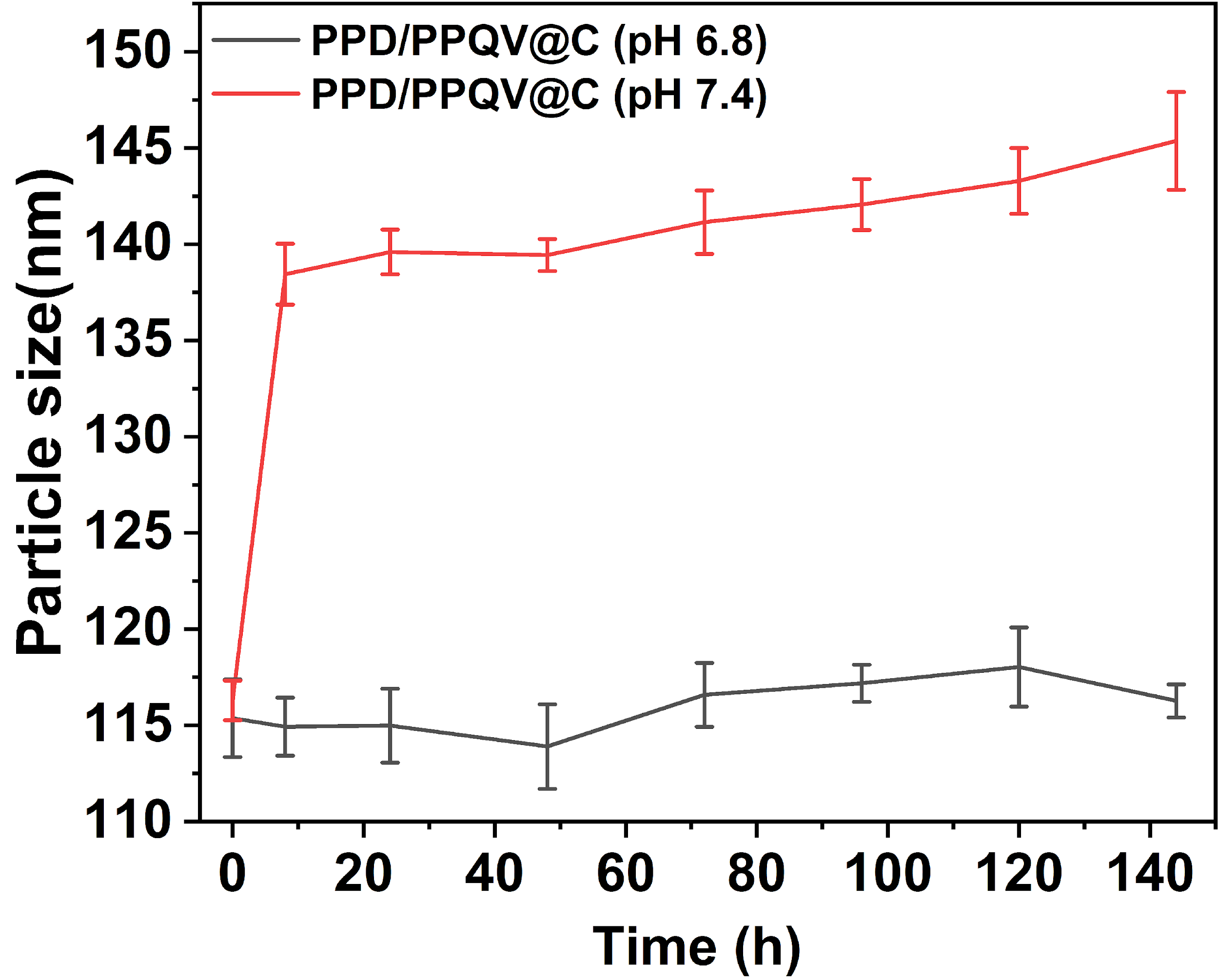


**Figure S8** Particle sizes of PPD/PPQV@C nanosystem incubated in 10% BSA at pH 7.4 and 6.8 for 6 days, detected by DLS. Error bars represent mean ± standard deviation (n = 3 independent samples).


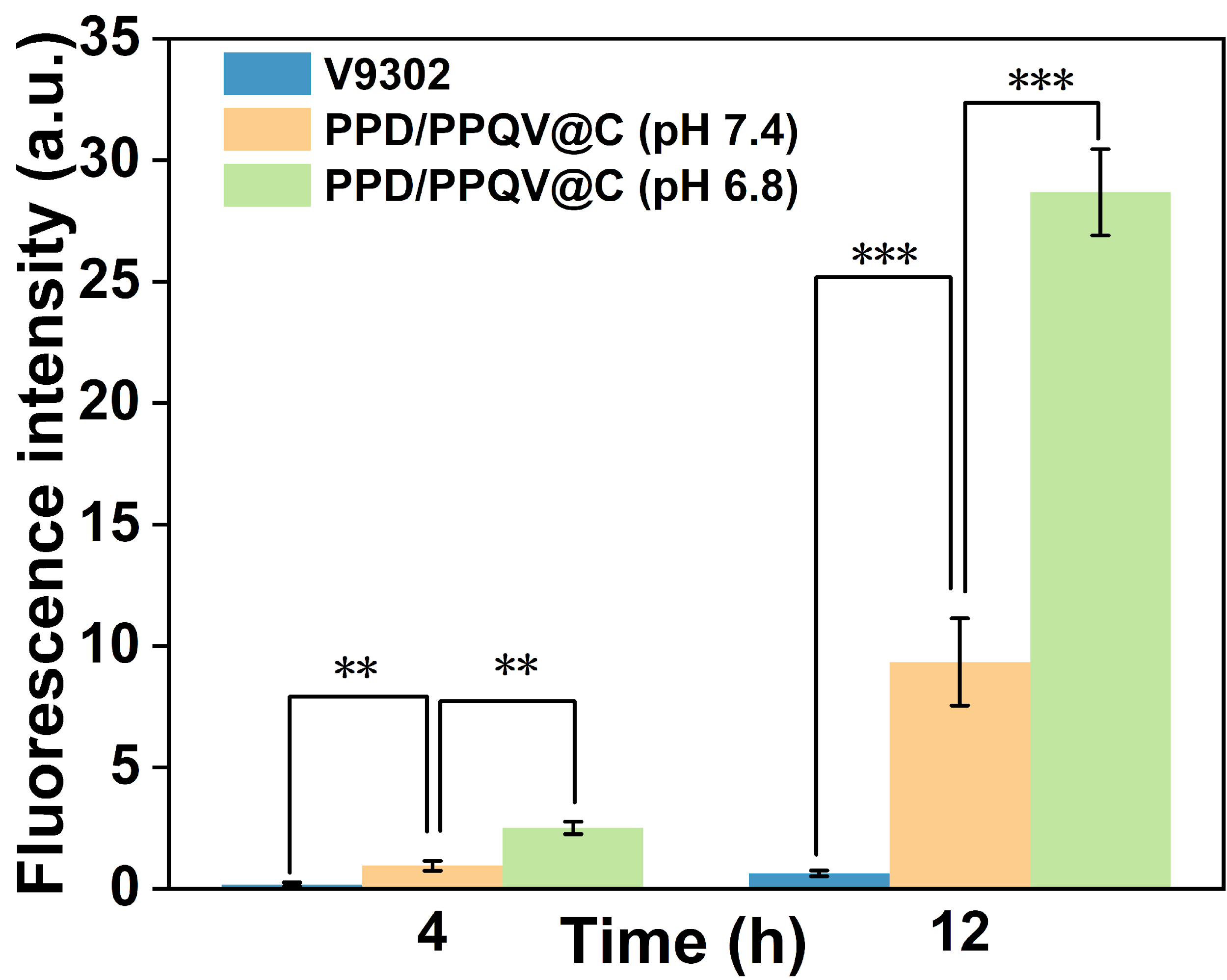


**Figure S9** Quantitative statistical analysis of fluorescence intensity of PPD/PPQV@C uptake by MDA-MB-231 cells at pH 7.4 and 6.8 for 4 h and 12 h. Error bars represent mean ± standard deviation (n = 4 biologically independent samples). The *P* values were determined by one-way ANOVA. ***p* < 0.01, ****p* < 0.001.


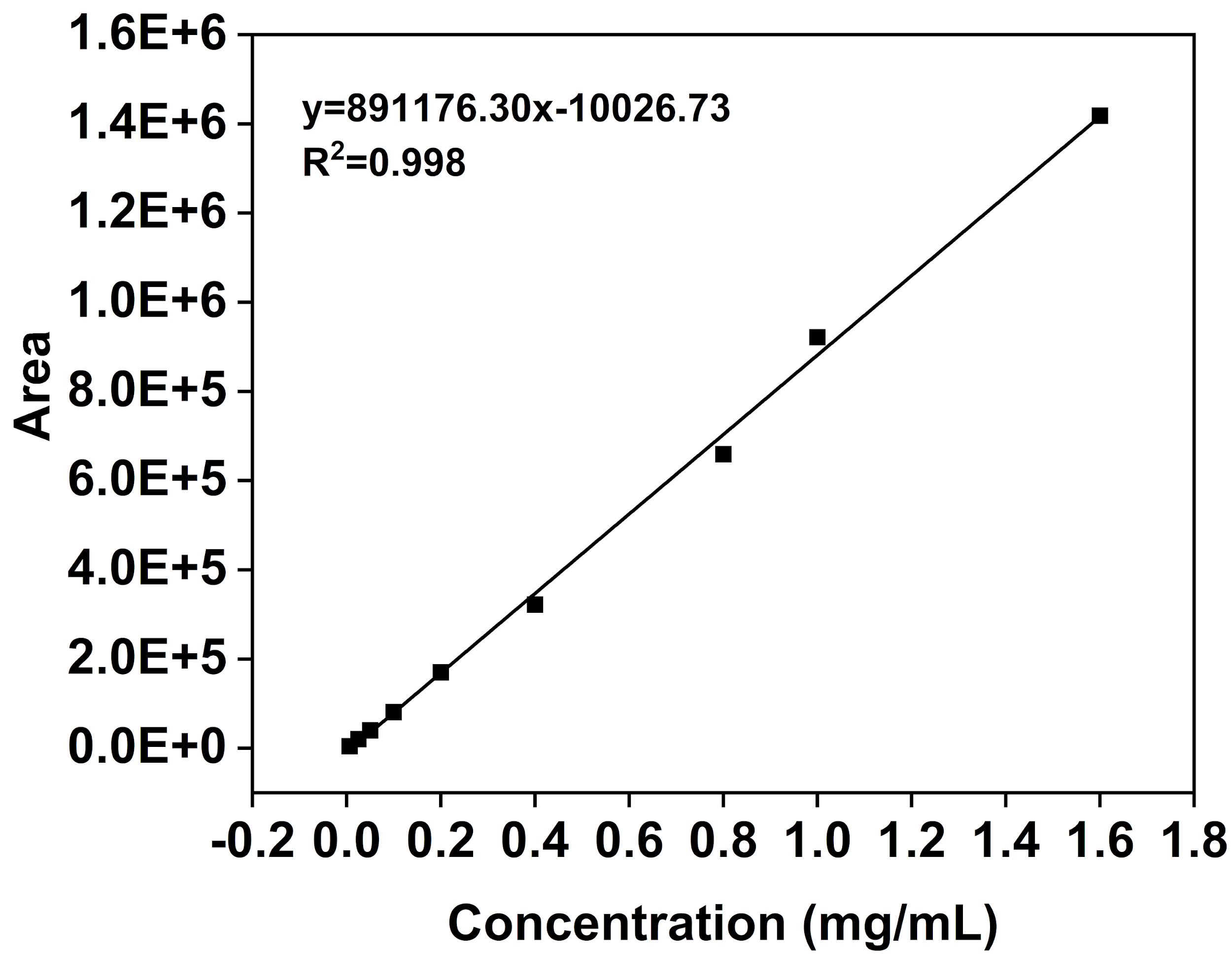


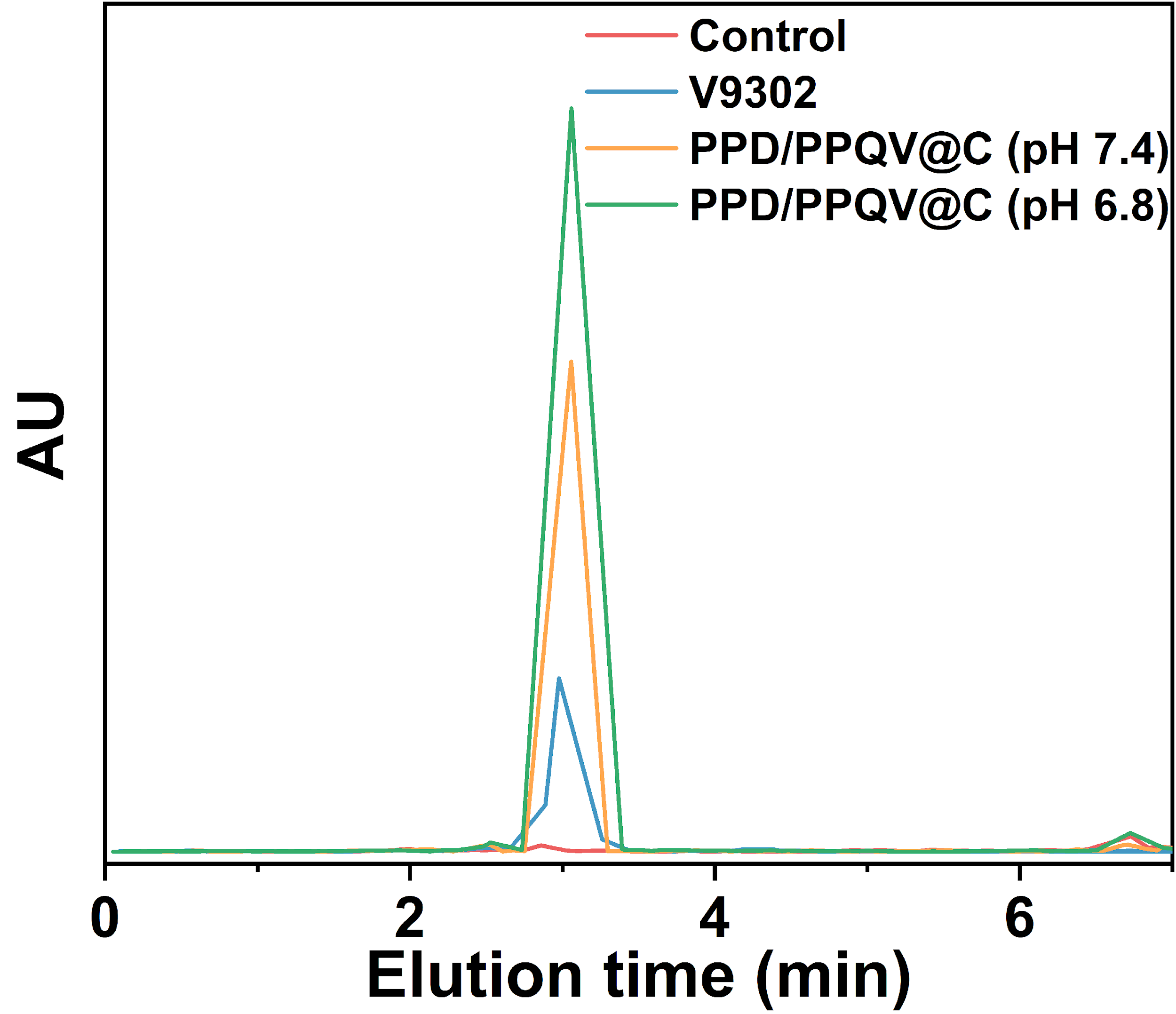


**Figure S10** Standard curve of V9302 and content of intracellular V9302 from MDA-MB-231 cells after incubation with various treatment groups for 12 h, detected by HPLC.


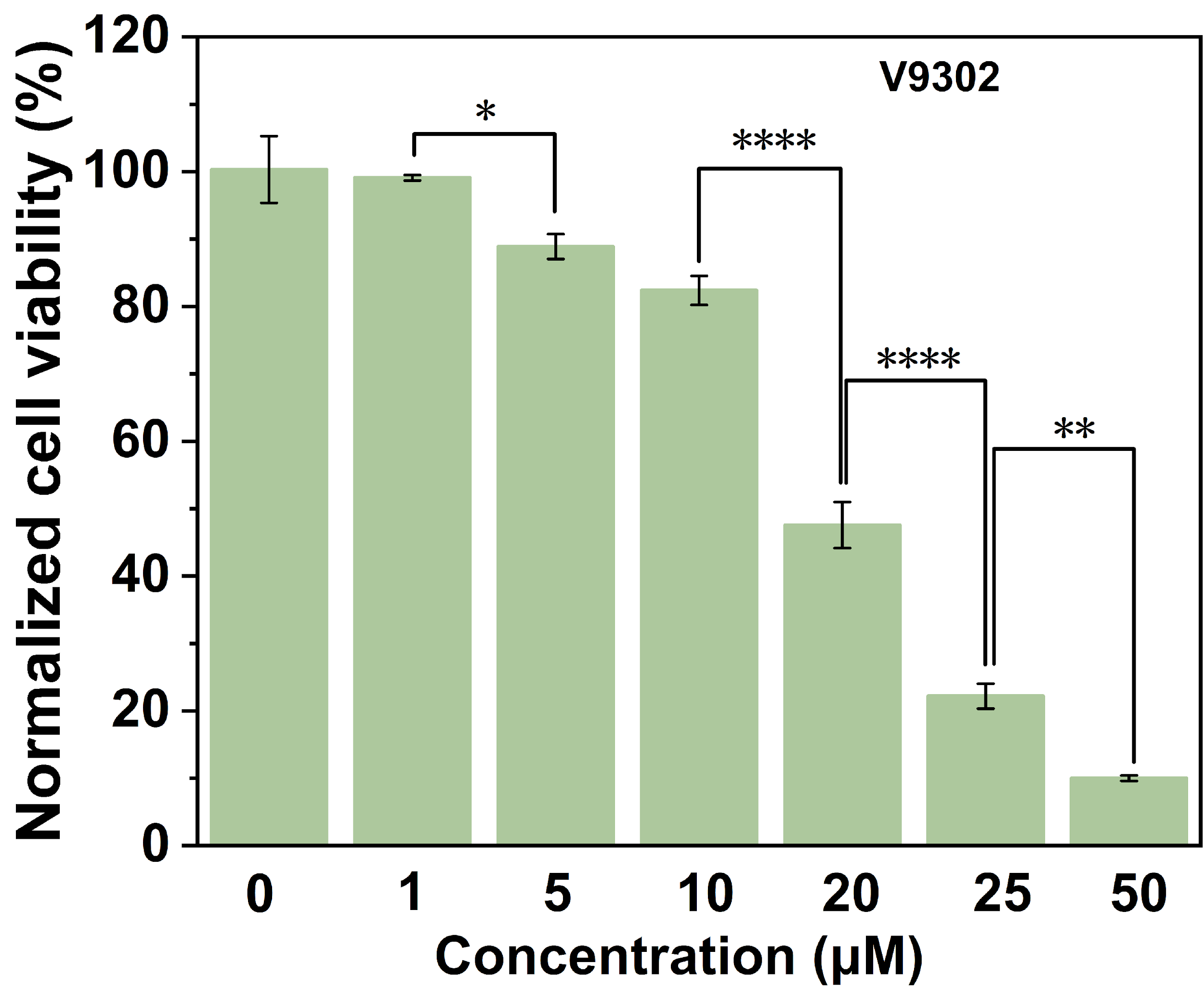


**Figure S11** Cytotoxicity of MDA-MB-231 cells co-incubated with different concentrations of V9302 for 24 h. Error bars represent mean ± standard deviation (n = 4 biologically independent samples). The *P* values were determined by one-way ANOVA. **p* < 0.05, ***p* < 0.01, *****p* < 0.0001.


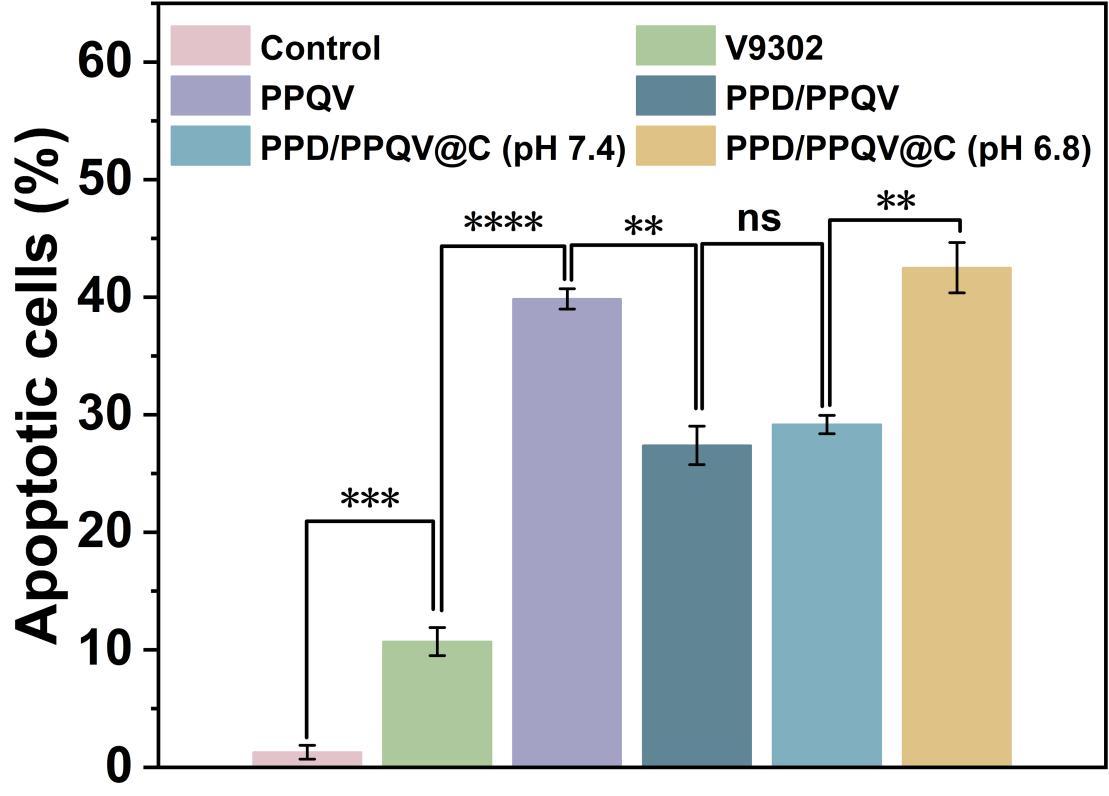


**Figure S12** Quantitative statistics of MDA-MB-231 cells apoptosis after different treatments for 24 h. Error bars represent mean ± standard deviation (n = 4 biologically independent samples). The *P* values were determined by one-way ANOVA. ***p* < 0.01, ****p* < 0.001, *****p* < 0.0001.


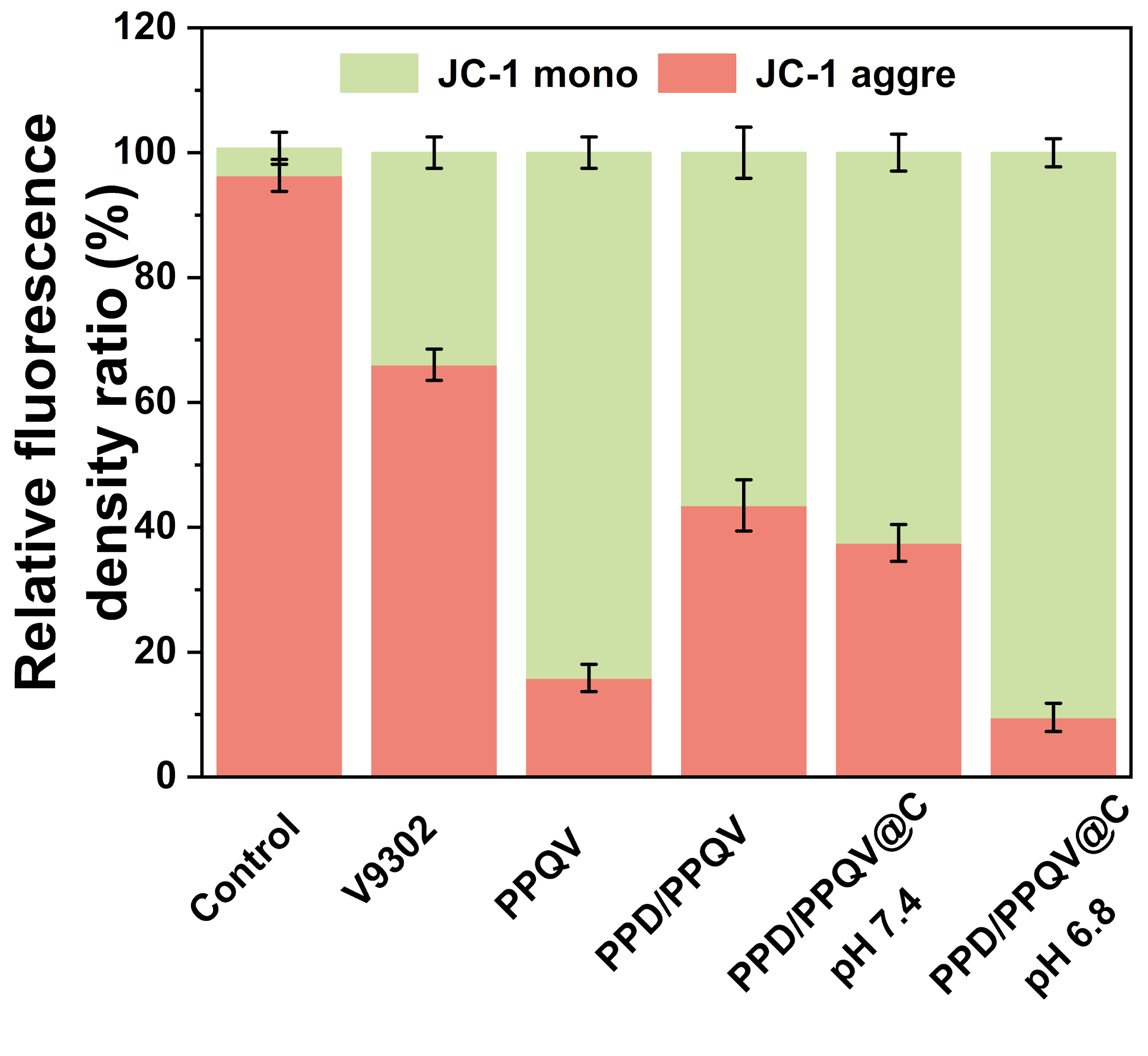


**Figure S13** Quantitative fluorescence density statistics of mitochondrial membrane potential after 12 h of administration, detected by JC-1 probe. Error bars present as mean ± SD (n = 4 biologically independent samples).

**
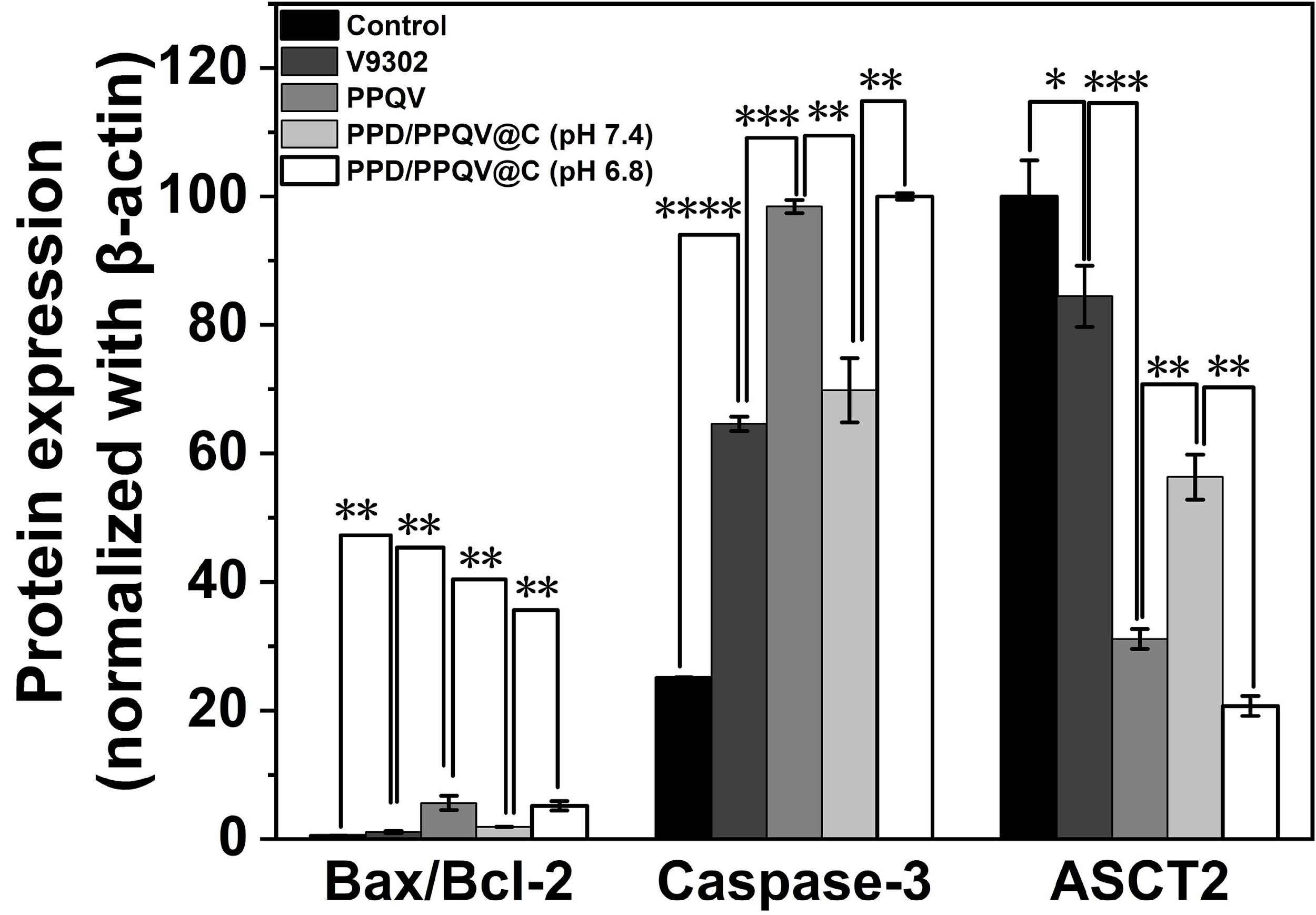
**

**Figure S14** Quantitative statistics of proteins expressions, based on western blotting data. Error bars present as mean ± SD (n = 3 biologically independent samples). The *P* values were determined by one-way ANOVA. **p* < 0.05, ***p* < 0.01, ****p* < 0.001, *****p* < 0.0001.


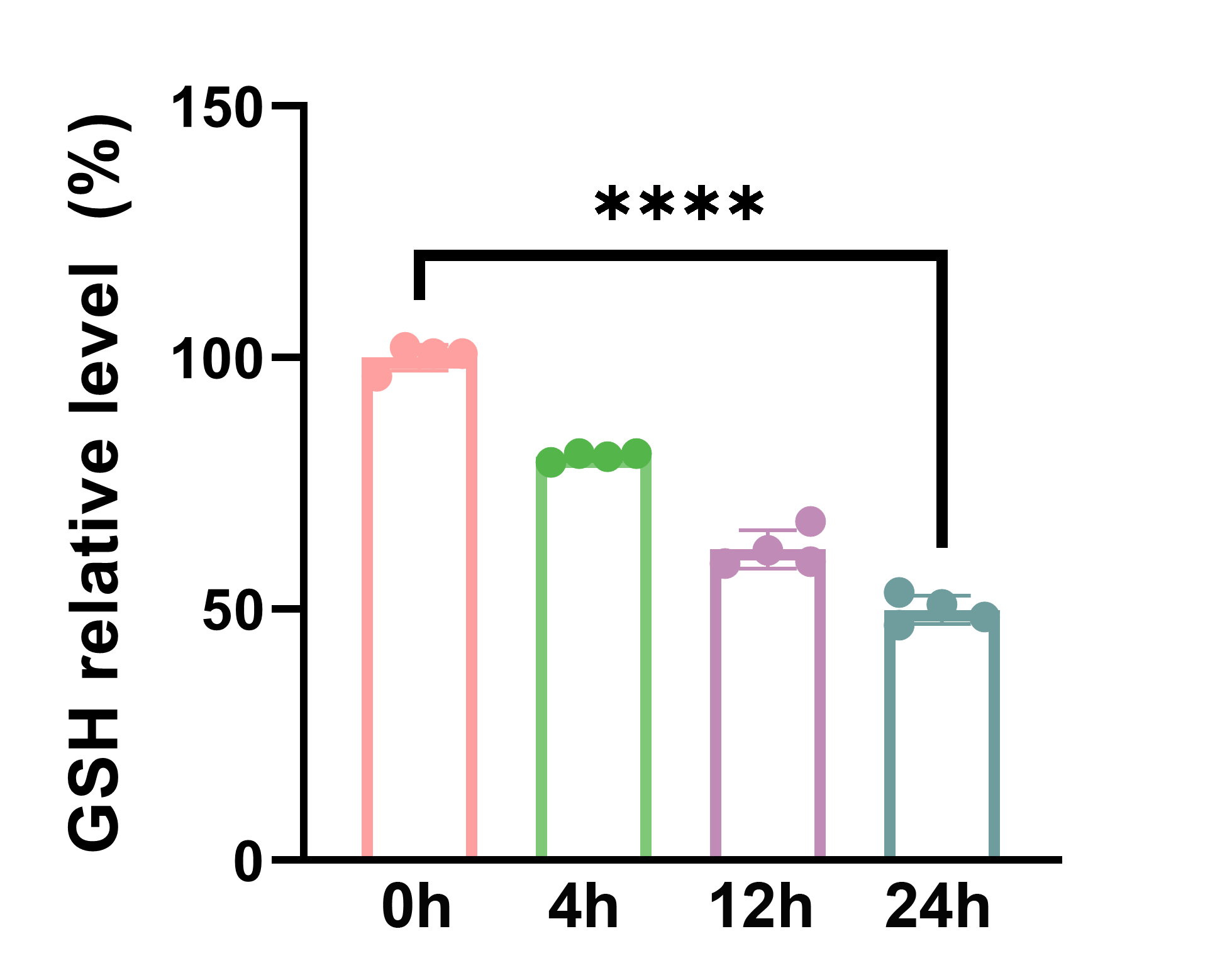


**Figure S15** Intracellular glutathione content at different administration times. Error bars present as mean ± SD (n = 4 biologically independent samples). The *P* values were determined by one-way ANOVA. *****p* < 0.0001.


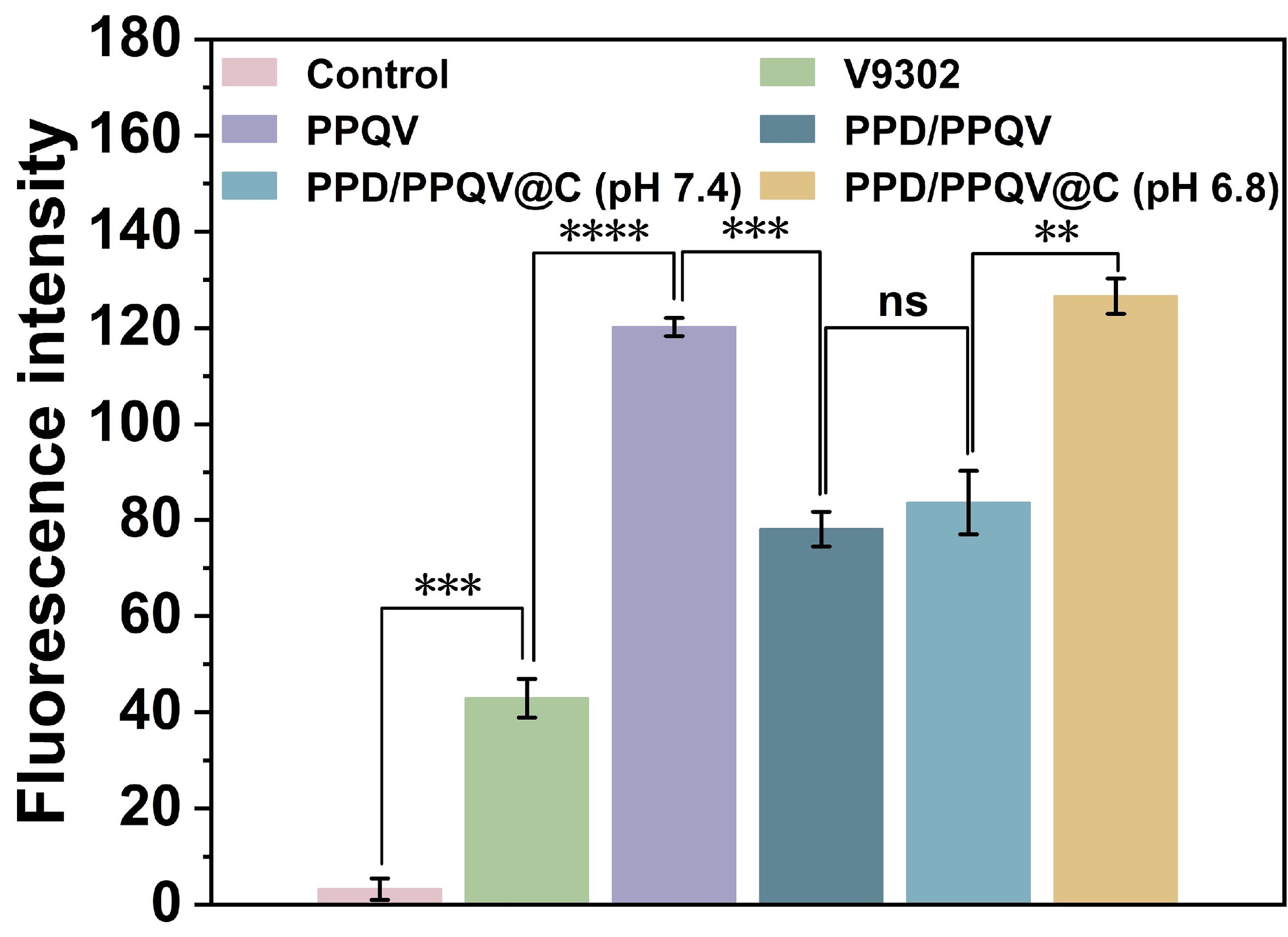


**Figure S16** Quantitative statistics of ROS after various treatments for 24 h *in vitro.* Error bars present as mean ± SD (n = 4 biologically independent samples). The *P* values were determined by one-way ANOVA. ***p* < 0.01, ****p* < 0.001, *****p* < 0.0001.


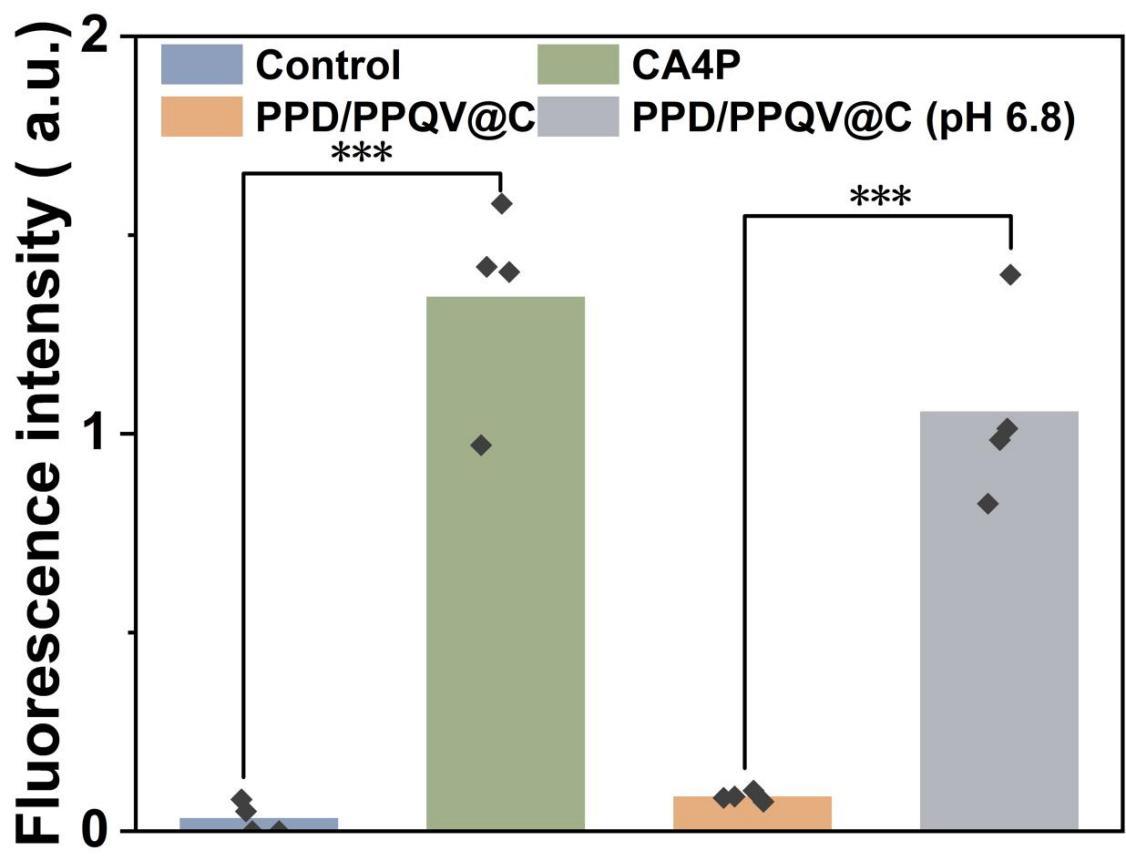


**Figure S17** Quantitative statistical analysis of fluorescence intensity of PPD/PPQV@C labelled with FITC uptake by HUVECs at pH 7.4 and pH 6.8 for 8 h. Error bars present as mean ± SD (n = 4 biologically independent samples). The *P* values were determined by one-way ANOVA. ****p* < 0.001.


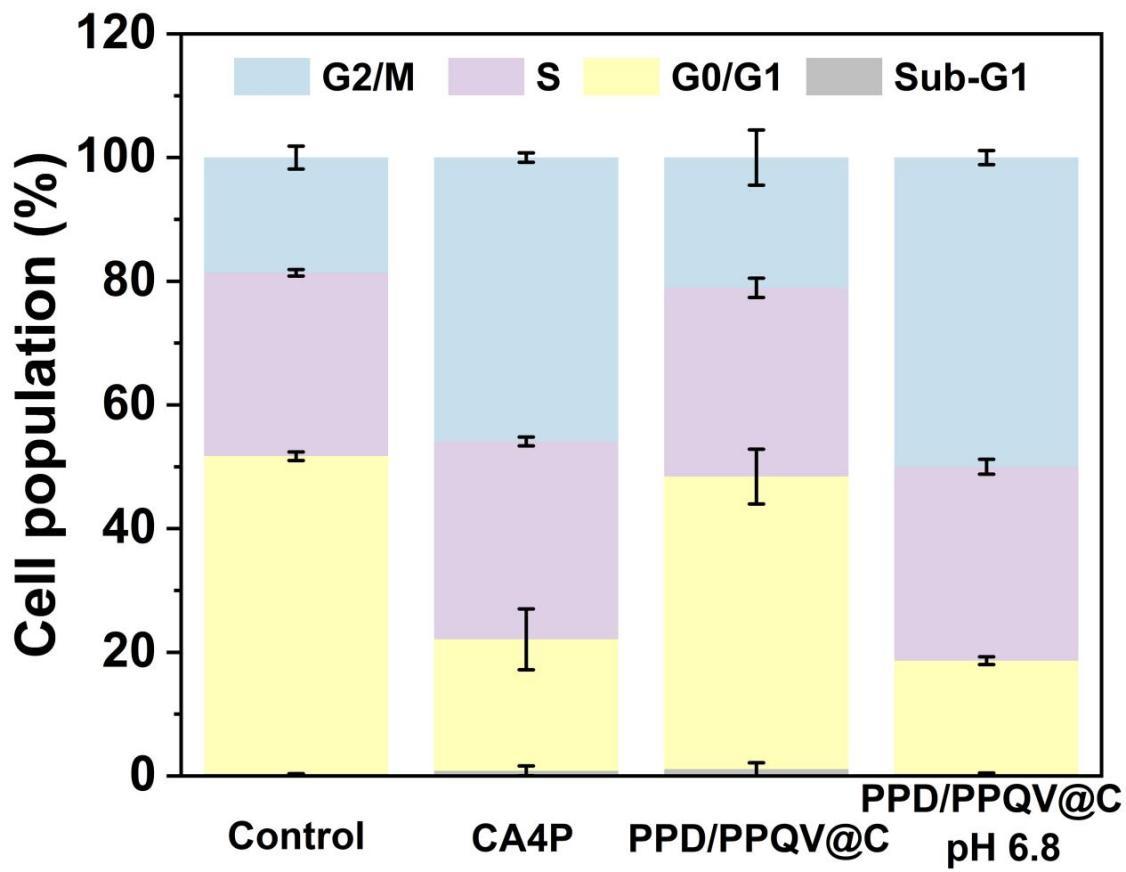


**Figure S18** Quantitative cell cycle analysis of HUVECs cells after administration for 24 h. Error bars present as mean ± SD (n = 4 biologically independent samples).

.


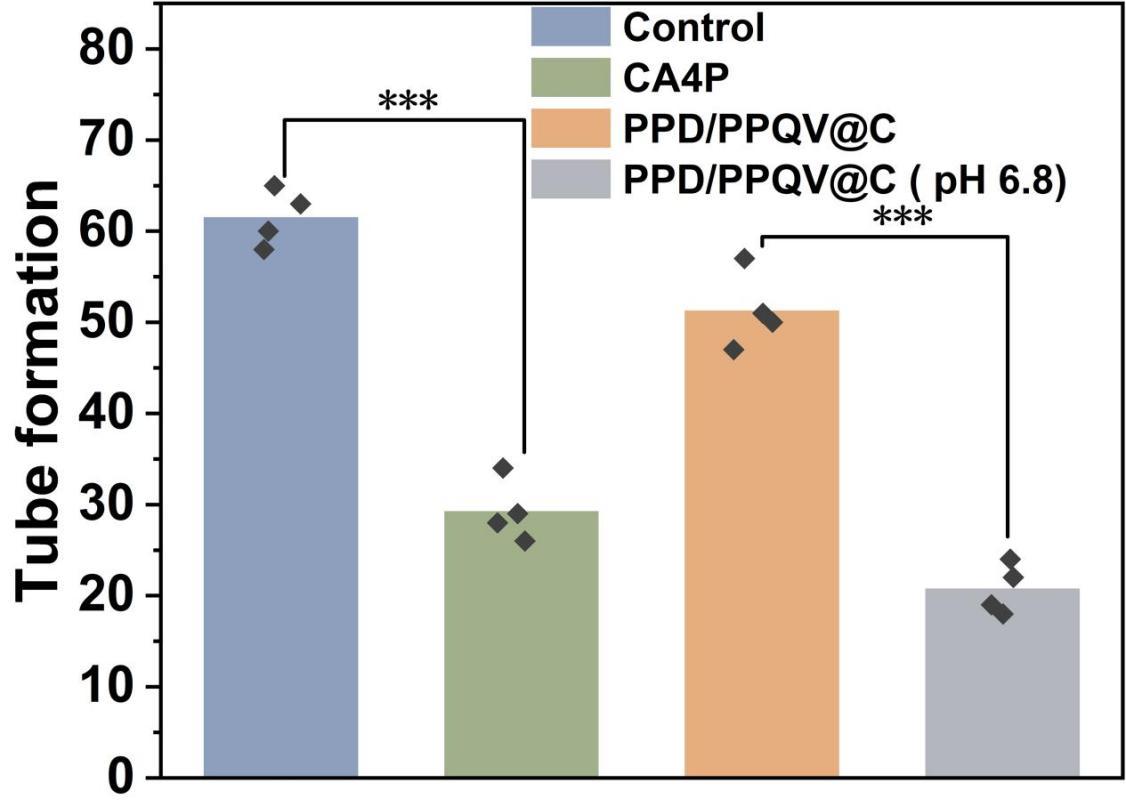


**Figure S19** Quantitative statistics of Tube formation after different administration for 6 h. Error bars present as mean ± SD (n = 4 biologically independent samples). The *P* values were determined by one-way ANOVA. ****p* < 0.001.


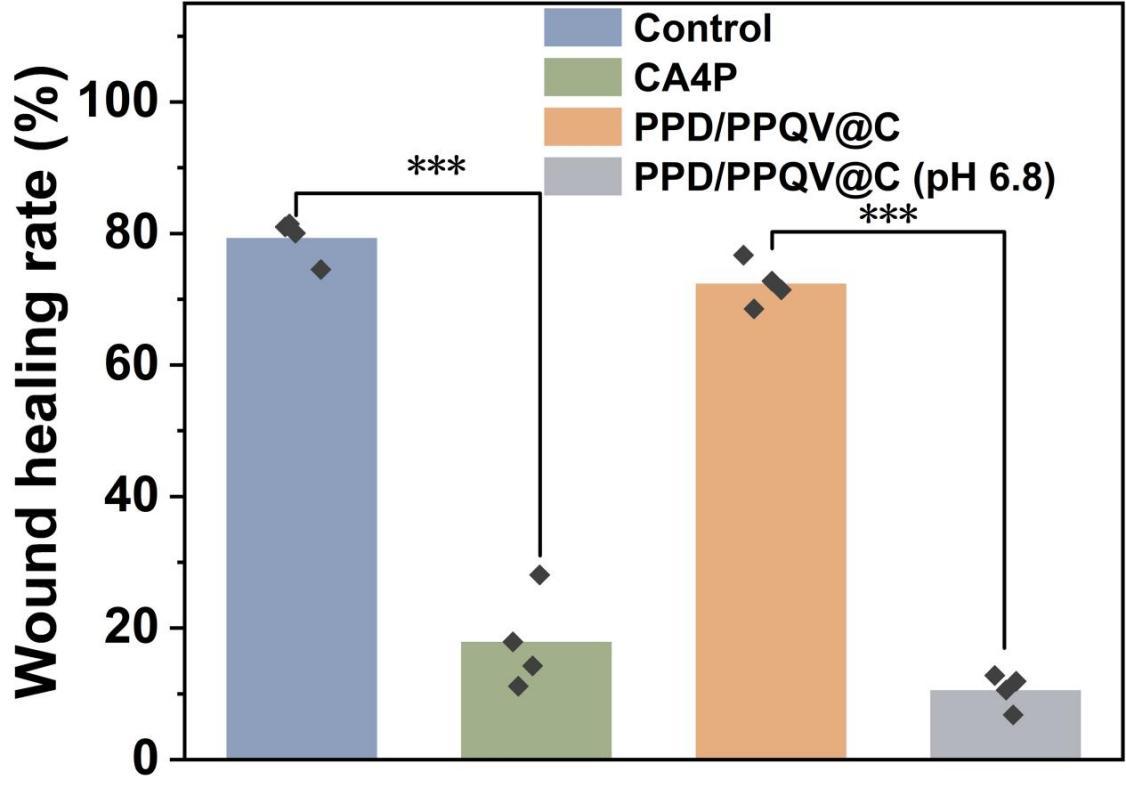


**Figure S20** Quantitative statistics of the wound healing ratio of HUVECs cells after different administration for 24 h. Error bars present as mean ± SD (n = 4 biologically independent samples). The *P* values were determined by one-way ANOVA. ****p* < 0.001.


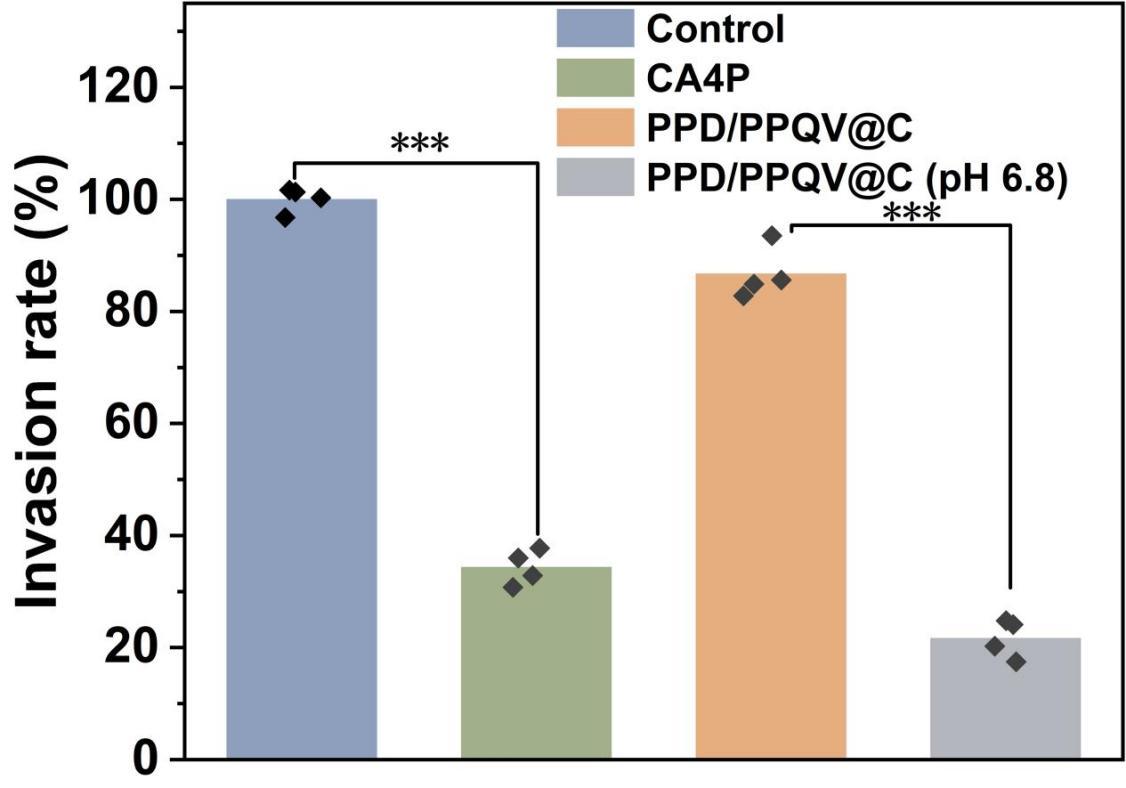


**Figure S21** Quantitative statistics of invasion rate of HUVECs cells after different administration for 12 h. Error bars present as mean ± SD (n = 4 biologically independent samples). The *P* values were determined by one-way ANOVA. ****p* < 0.001.


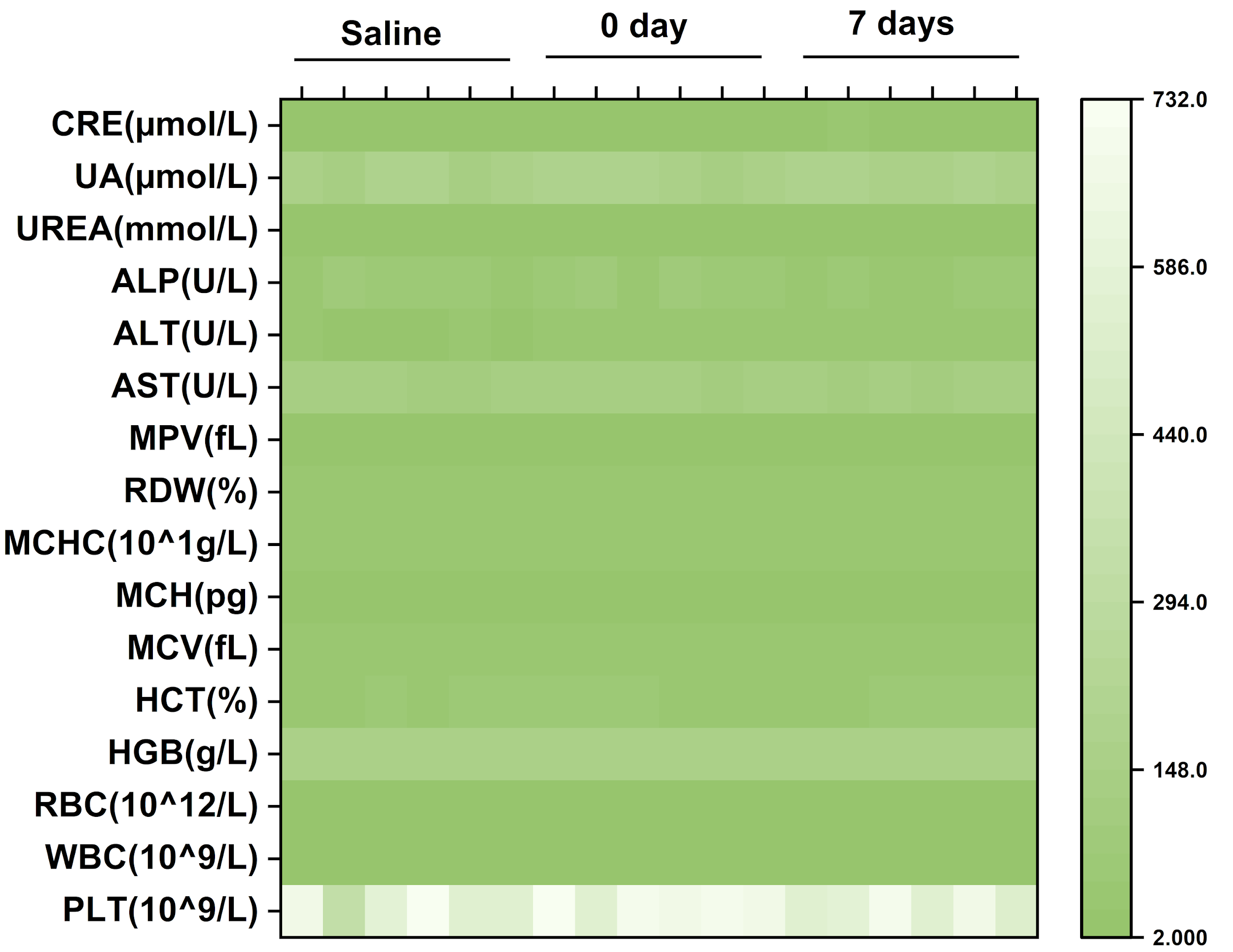


**Figure S22** Heat map of mouse blood routine of MDA-MB-231 tumor bearing Balb/c nude mice after treatments with various systems for 7 days. Error bars present as mean ± SD (n=6).


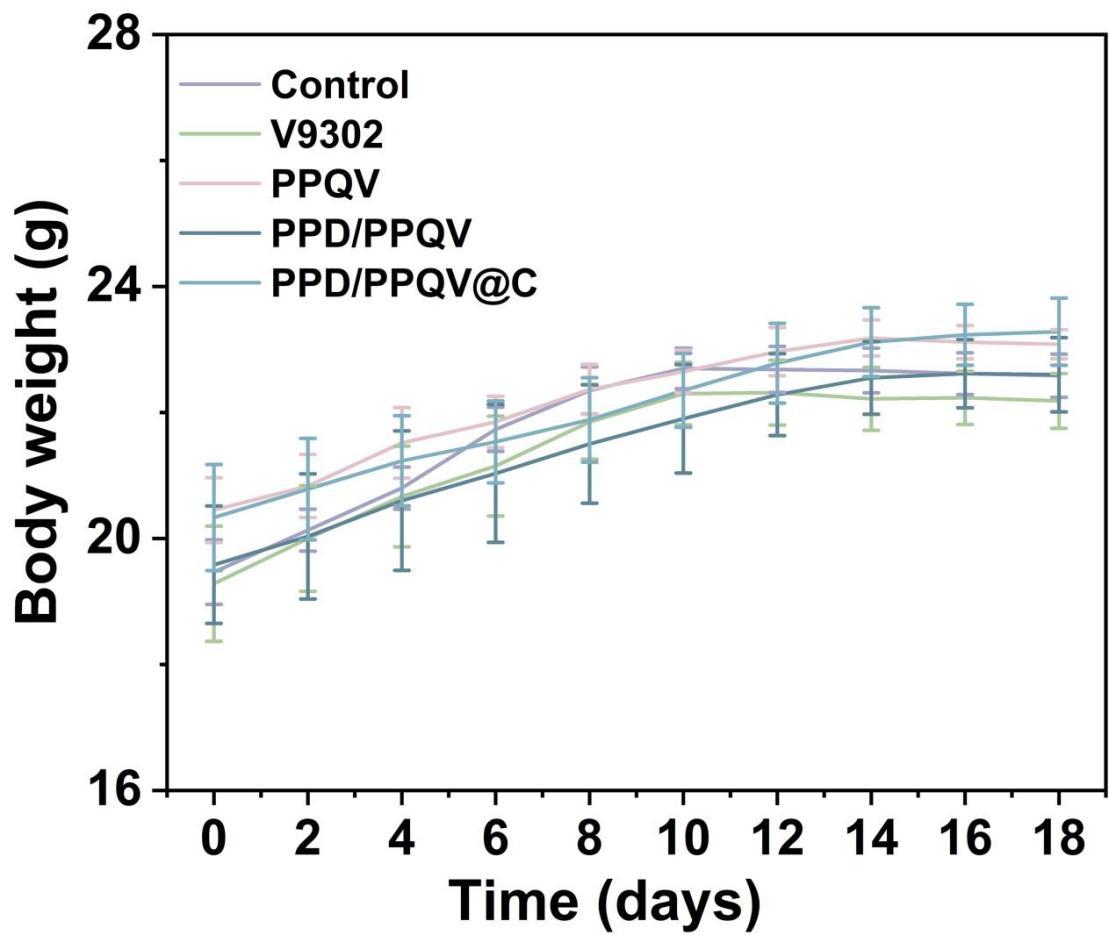


**Figure S23** Weight change curves of MDA-MB-231-bearing Balb/c nude after different treatments for 18 days. Error bars present as mean ± SD (n=6).


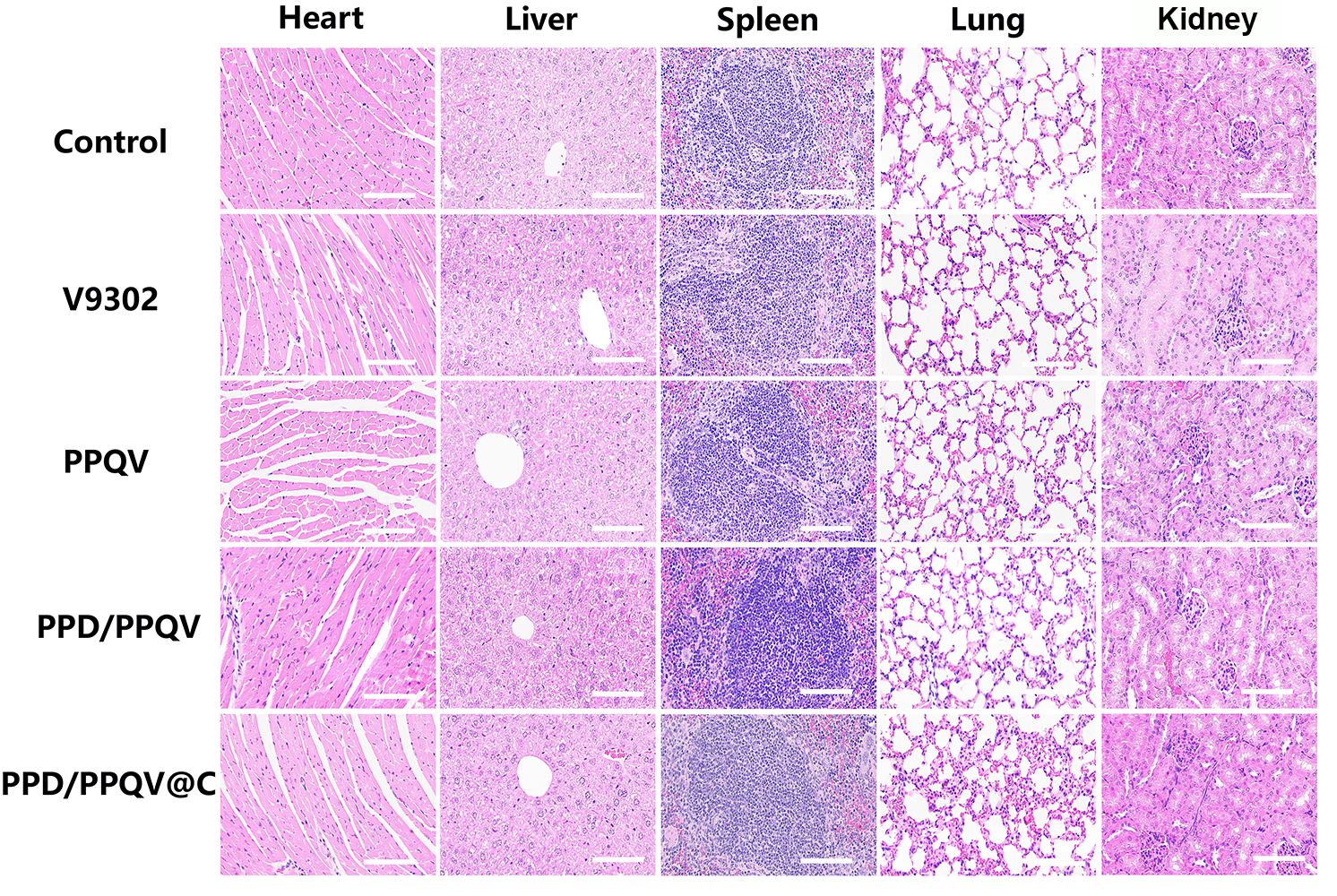


**Figure S24** H&E staining analysis of major tissues (heart, liver, spleen, lung and kidney) of MDA-MB-231 tumor bearing Balb/c nude mice after different treatments for 18 days. Scale bar: 50 μm.


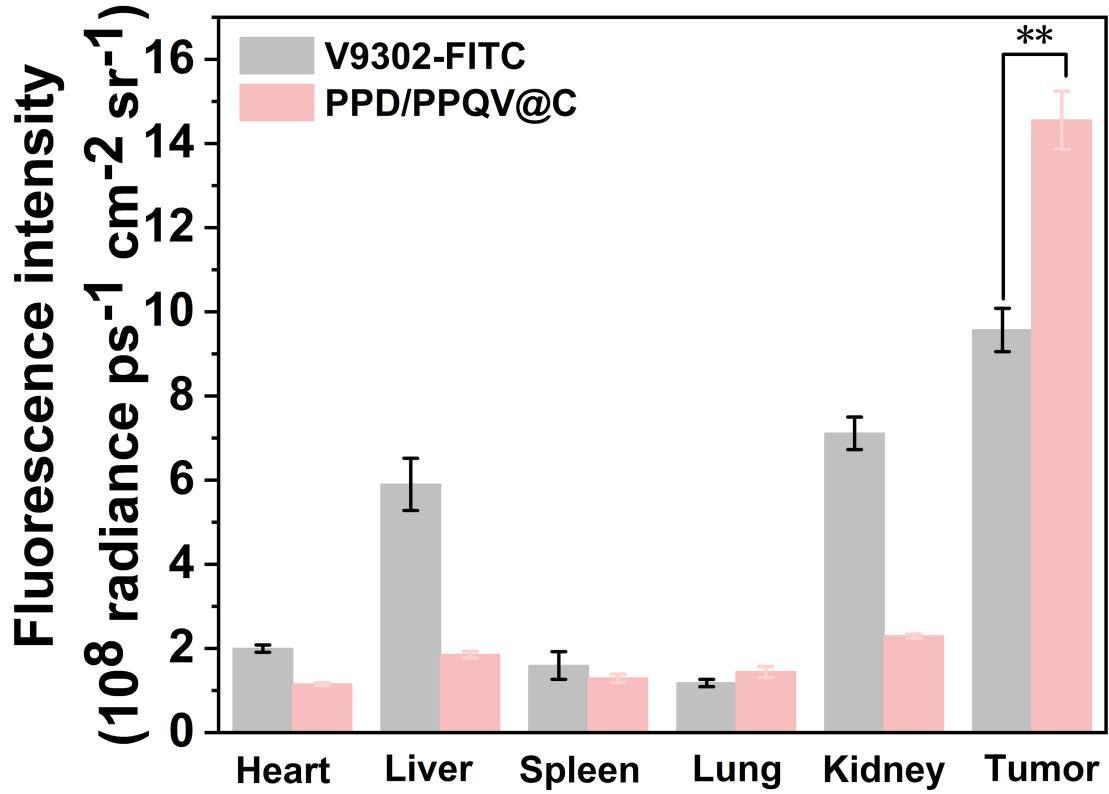


**Figure S25** Quantitative fluorescence analysis of biodistribution in major organs of MDA-MB-231-bearing mice after 6 h of injection V9302 labelled with FTIC and PPD/PPQC@C. Error bars present as mean ± SD (n = 6 biologically independent samples). The *P* values were determined by one-way ANOVA. ***p* < 0.01.


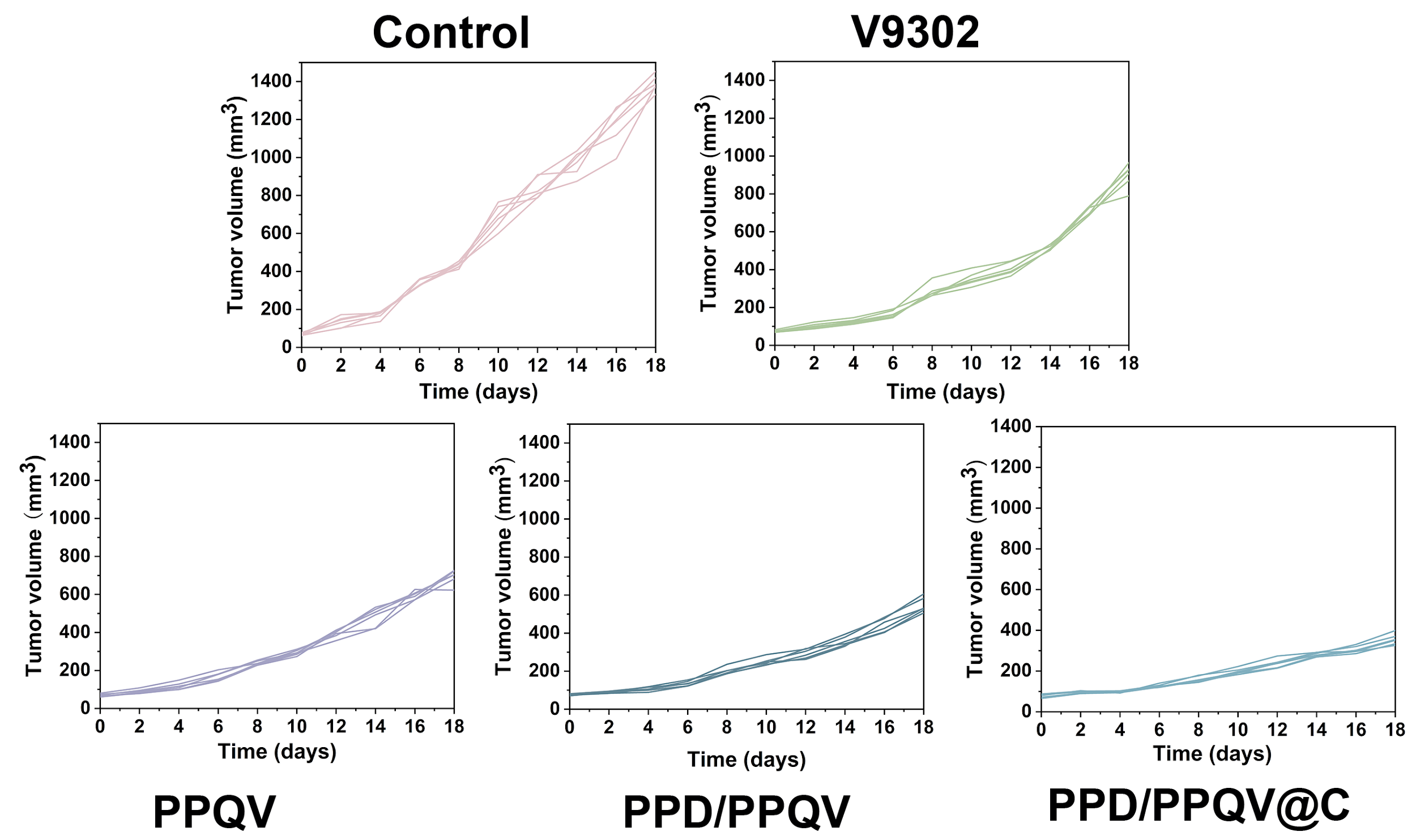


**Figure S26** Tumor volume change curves of the mice in all administrations upon treatment period. (n = 6 biologically independent samples).


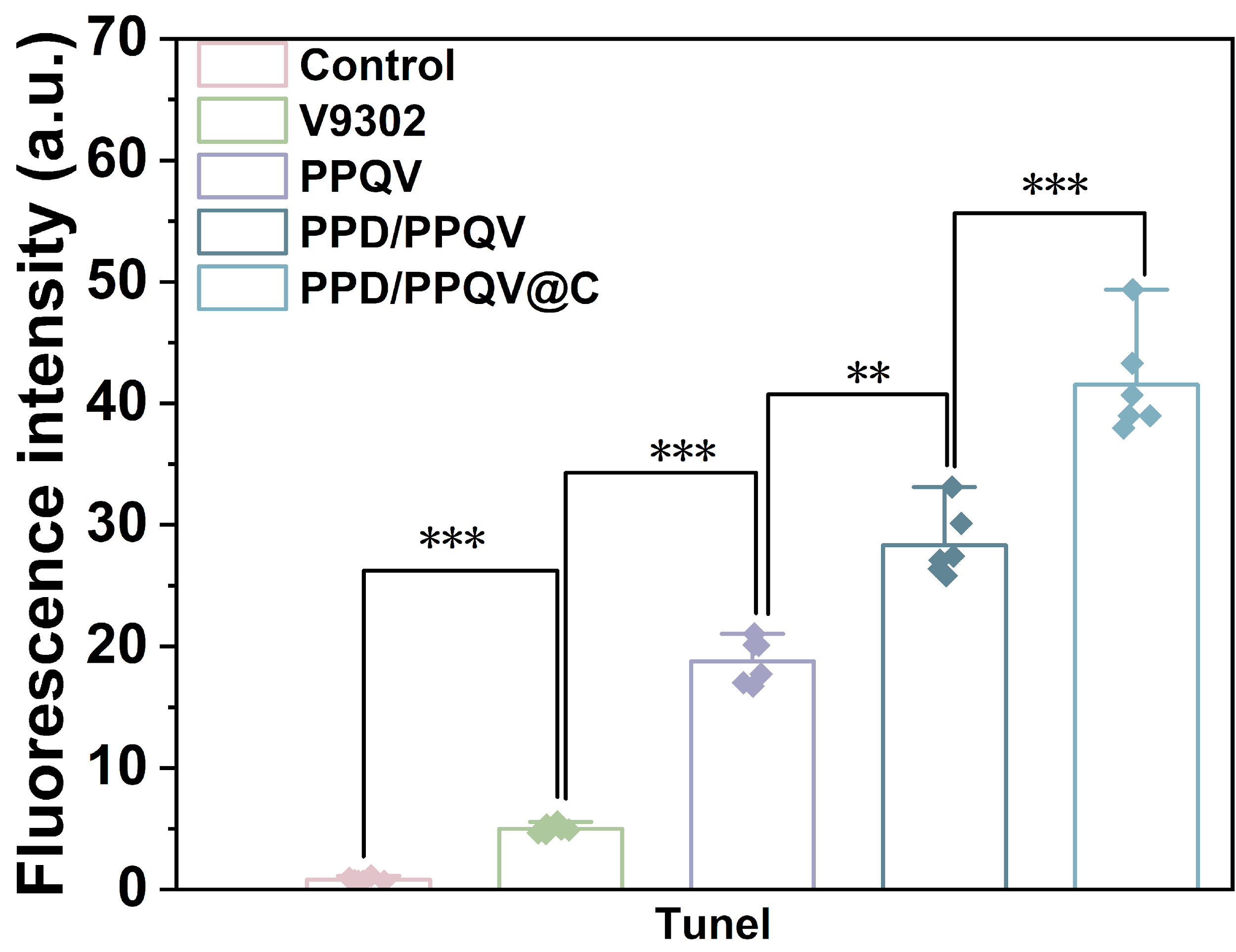


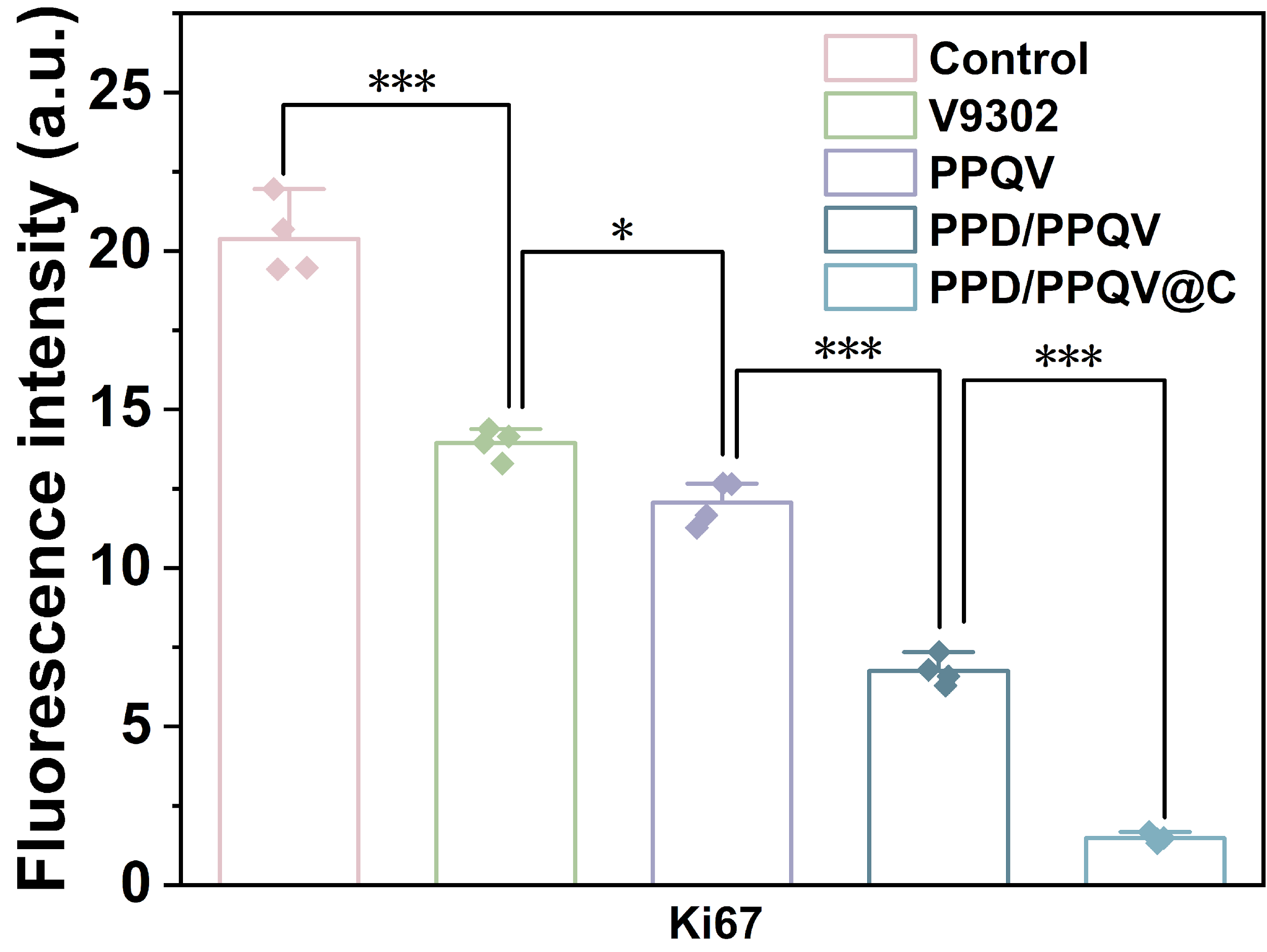

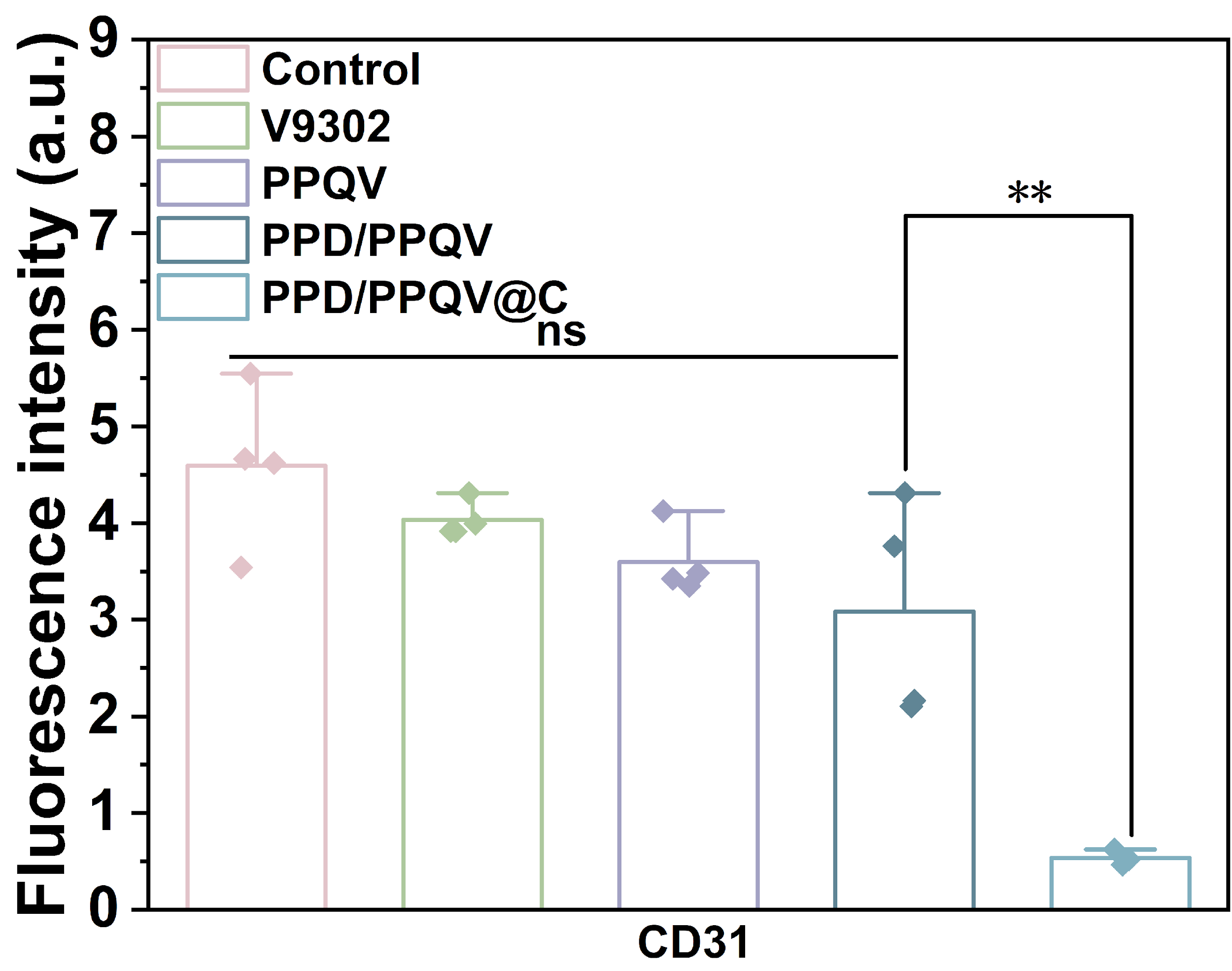


**Figure** **S27** Quantitative statistics of TUNEL, Ki67 and CD31. Error bars present as mean ± SD (n = 6 biologically independent samples). The *P* values were determined by one-way ANOVA. **p* < 0.05, ***p* < 0.01, ****p* < 0.001.


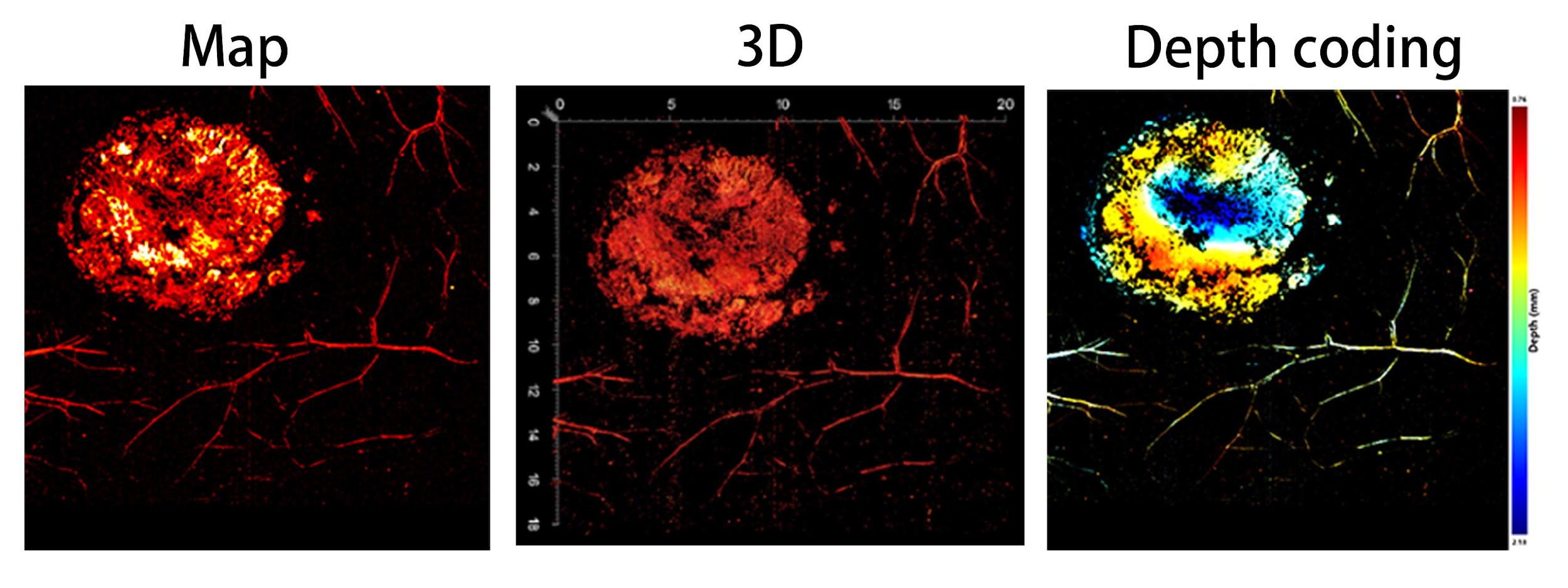


**Figure S28** Images of blood vessels in mice after free CA4P administration.


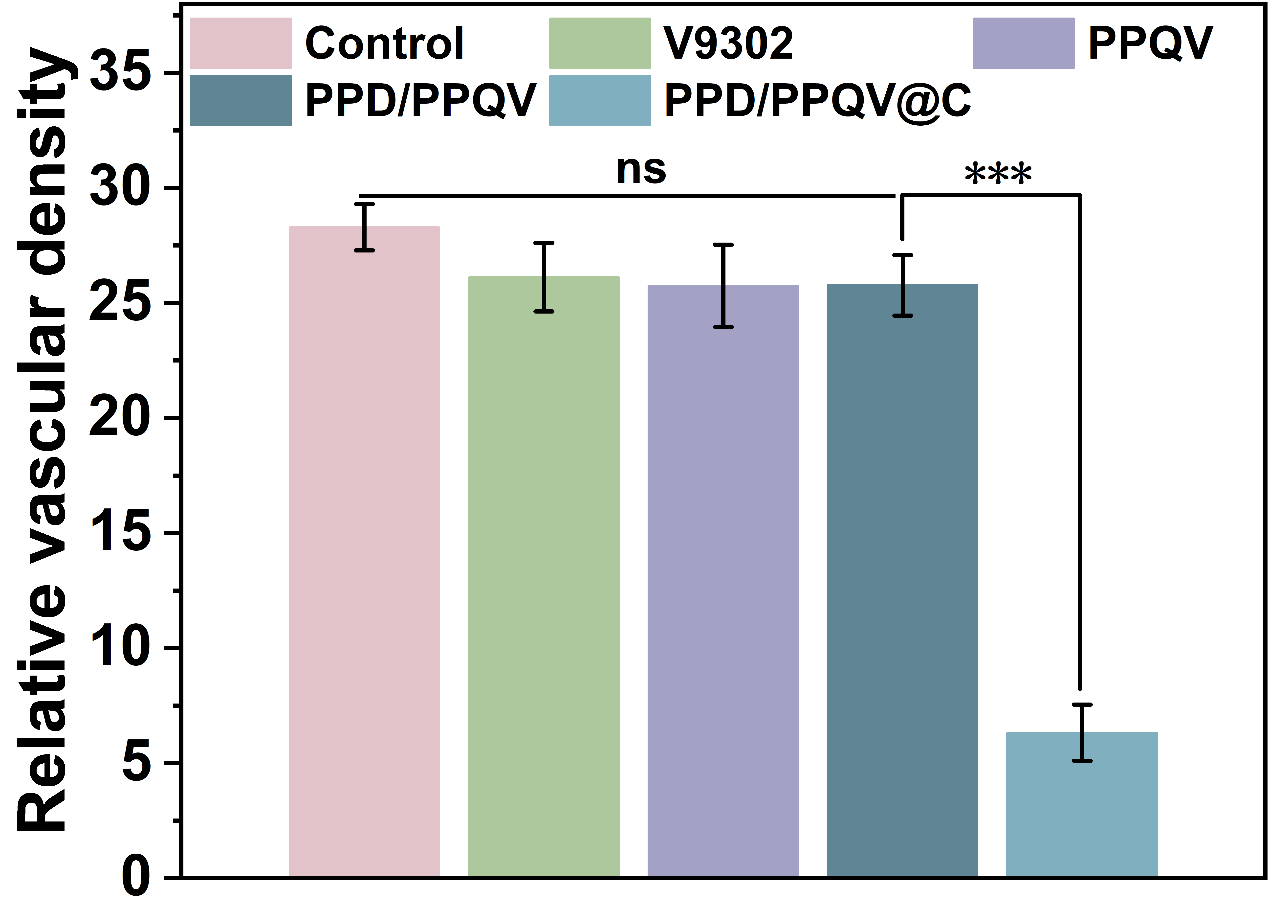


**Figure S29** Quantitative statistics of tumor vascular density. The *P* values were determined by one-way ANOVA. ****p* < 0.001
